# Predicting implementation of active learning by tenure-track teaching faculty using robust cluster analysis

**DOI:** 10.1186/s40594-022-00365-9

**Published:** 2022-07-28

**Authors:** Kameryn Denaro, Petra Kranzfelder, Melinda T. Owens, Brian Sato, Austin L. Zuckerman, Rebecca A. Hardesty, Adriana Signorini, Andrea Aebersold, Mayank Verma, Stanley M. Lo

**Affiliations:** 1 Division of Teaching Excellence and Innovation, University of California Irvine, 653 E Peltason Drive, Irvine, CA 92697 USA; 2 Molecular and Cellular Biology, University of California Merced, 5200 North Lake Road, Merced, CA 95343 USA; 3 Section of Neurobiology, Division of Biological Sciences, University of California San Diego, 9500 Gilman Drive, La Jolla, CA 92093 USA; 4 Program in Mathematics and Science Education, University of California San Diego, 9500 Gilman Drive, La Jolla, CA 92093 USA; 5 Molecular Biology and Biochemistry, University of California Irvine, 2238 McGaugh Hall, Irvine, CA 92697 USA; 6 Section of Cell and Developmental Biology, Division of Biological Sciences, University of California San Diego, 9500 Gilman Drive, La Jolla, CA 92093 USA; 7 Division of Biological Sciences, University of California San Diego, 9500 Gilman Drive, La Jolla, CA 92093 USA; 8 Students Assessing Teaching and Learning (SATAL) Program, Center for Engaged Teaching and Learning, University of California Merced, 5200 North Lake Road, Merced, CA 95343 USA

**Keywords:** Active learning, COPUS, Higher education, STEM, Lecturer with security of employment, Professor of Teaching, Teaching Professor, Teaching focused faculty, Ensemble methods, Robust clustering

## Abstract

**Background:**

The University of California system has a novel tenure-track education-focused faculty position called Lecturer with Security of Employment (working titles: Teaching Professor or Professor of Teaching). We focus on the potential difference in implementation of active-learning strategies by faculty type, including tenure-track education-focused faculty, tenure-track research-focused faculty, and non-tenure-track lecturers. In addition, we consider other instructor characteristics (faculty rank, years of teaching, and gender) and classroom characteristics (campus, discipline, and class size). We use a robust clustering algorithm to determine the number of clusters, identify instructors using active learning, and to understand the instructor and classroom characteristics in relation to the adoption of active-learning strategies.

**Results:**

We observed 125 science, technology, engineering, and mathematics (STEM) undergraduate courses at three University of California campuses using the Classroom Observation Protocol for Undergraduate STEM to examine active-learning strategies implemented in the classroom. Tenure-track education-focused faculty are more likely to teach with active-learning strategies compared to tenure-track research-focused faculty. Instructor and classroom characteristics that are also related to active learning include campus, discipline, and class size. The campus with initiatives and programs to support undergraduate STEM education is more likely to have instructors who adopt active-learning strategies. There is no difference in instructors in the Biological Sciences, Engineering, or Information and Computer Sciences disciplines who teach actively. However, instructors in the Physical Sciences are less likely to teach actively. Smaller class sizes also tend to have instructors who teach more actively.

**Conclusions:**

The novel tenure-track education-focused faculty position within the University of California system represents a formal structure that results in higher adoption of active-learning strategies in undergraduate STEM education. Campus context and evolving expectations of the position (faculty rank) contribute to the symbols related to learning and teaching that correlate with differential implementation of active learning.

**Supplementary Information:**

The online version contains supplementary material available at 10.1186/s40594-022-00365-9.

## Introduction

Evidence-based instructional practices (Landrum et al. 2017), including various active-learning strategies (Driessen 2020; Lombardi et al., 2021), improve cognitive outcomes (Péerez-Sabater et al., 2011; Schwartz et al., 2011; Styers et al., 2018; Vanags et al., 2013) and persistence of students (Brax-ton et al., 2008; Kuh et al., 2008) in science, technology, engineering, and mathematics (STEM) majors compared with traditional lecture-based instruction (President’s Council of Advisors on Science and Technology, 2012). Especially of significance, active-learning strategies disproportionately support students from racially or ethnically minoritized backgrounds on average; thus reducing equity gaps in academic achievement (Haak et al., 2011, Maries et al., 2020, Theobald et al., 2020). Even though widespread and immediate implementation of active-learning strategies should be a high priority in undergraduate STEM education (Theobald et al., 2020), adoption remains low, and most courses are still taught using traditional, lecture-based instruction (Stains et al., 2018). For this study, we used the Classroom Observation Protocol for Undergraduate STEM (COPUS) (Smith et al., 2013) to obtain a quantitative measure of the amount of active learning occurring in the classroom, a commonly used protocol for measuring active learning at department-wide (Cotner et al., 2017; Kranzfelder et al., 2019), institution-wide (Akiha et al., 2018; Lewin et al., 2016; Lund et al., 2015; Lund & Stains, 2015; Meaders et al., 2019; Smith et al., 2014 ; Tomkin et al., 2019), and multi-institution-wide scales (Borda et al., 2020 ; Lane et al., 2021; Stains et al., 2018). Rather than focus on a particular definition of active learning, we use COPUS to focus our work on instructor and student behaviors and how those are related to instructor and classroom characteristics.

In this paper, we examine the potential difference in implementation of active-learning strategies by faculty type, including tenure-track education-focused faculty, tenure-track research-focused faculty, and non-tenure-track lecturers. The University of California (UC) system has a novel tenure-track education-focused faculty position called the Lecturer with Security of Employment (Harlow et al., 2020; Xu & Solanki 2020), to which we will refer using its working title across different UC campuses: Teaching Professor or Professor of Teaching (TP/PoT). Similar to tenure-track research-focused faculty, TP/PoTs are evaluated for promotion and tenure based on their activities in scholarship, teaching, and service, but unlike tenure-track research-focused faculty, there is an increased emphasis on teaching (University of California Office of the President, 2018). For scholarship, many TP/PoTs engage in discipline-based education research (DBER), evidence-based curriculum development, outreach, and student mentorship (Harlow et al., 2020). In contrast to non-tenure-track lecturers hired on a fixed-term contract (American Association of University Professors, 2014, 2018; Carvalho & Diogo 2018), the TP/PoT position has the protection of tenure and are voting members of the Academic Senate (University of California Office of the President, 2018). Research-focused universities often prioritize and incentivize research productivity over teaching (Diamond & Adam, 1998; Savkar & Lokere, 2010; Schimanski & Alperin, 2018), and TP/PoTs may be institutionally (with tenure) and professionally (with expertise) situated to make changes in undergraduate STEM education by implementing active-learning strategies in their courses.

Because COPUS makes use of 25 distinct codes, COPUS results can be difficult to analyze. Most research studies use COPUS data in descriptive form and highlight particular codes of interest if they vary across study groups (Akiha et al., 2018; Jiang & Li, 2018; Kranzfelder et al., 2019; Lewin et al., 2016; Liu et al., 2018; McVey et al., 2017; Reisner et al., 2020; Smith et al., 2013; Solomon et al., 2018; Weaver & Burgess, 2015). For example, Tomkin et al. (Tomkin et al., 2019). identified differences in the frequency of various COPUS codes between faculty who did and did not participate in professional development. Since there are many COPUS codes to explore, these aforementioned studies are prone to the “winner’s curse” (the difficulty in reproducing significant findings, where large number of tests are conducted) (Forstmeier & Schielzeth, 2011) and issues with multiple testing (Hsu, 1996; Tukey, 1991). In addition, by only considering one code at a time (for example, percent of time spent lecturing), the researchers, maybe unintentionally, have operationally defined “active learning” more narrowly than may be appropriate (for example, as anything antithetical to lecture).

Another approach to explore COPUS data is cluster analysis (Denaro et al., 2021; Lund et al., 2015, Stains et al., 2018), which enables the characterization of a course by identifying distinct patterns of instructor and student behaviors in the classroom. Cluster analysis avoids issues with testing multiple single codes by considering overall patterns of many codes together. In addition, by using multiple methods of cluster analysis and pooling the results with ensemble methods, we avoid prescribing what patterns of teaching may be characteristic of an active learning classroom through examining many different ways to group such patterns. Our goal is to leverage cluster analysis to consider a variety of ways in which an instructor could implement active-learning strategies, consolidate that information, and then identify instructor and classroom characteristics that correlate with greater implementation of active learning.

In this paper, we explore instructional practices across three different UC campuses through using COPUS. With these data, we identify the extent to which implementation of active-learning strategies is related to instructor and classroom characteristics. Specifically, we will address the following research questions (RQs) about data collected in the UC system:

1. To what extent are TP/PoTs more likely to implement active-learning strategies compared to non-tenure track lecturers and tenure-track research faculty?
2. What instructor and classroom characteristics correlate with active-learning?

## Literature review

### Tenure-track teaching faculty position

The TP/PoT position represents a formal institutional structure in the UC system, existing as a specific academic title code with its own definitions and promotion criteria (University of California Office of the President, 2018). TP/PoTs are viewed by administrators as education experts to take on substantial teaching responsibilities, coordinate assessment efforts, and provide professional development within departments Harlow et al. (2021). However, it is an open question whether this perceived pedagogical expertise is actually reflected in their instructional practices, for example in their implementation of more active-learning strategies as compared to tenure-track research-focused faculty and non-tenure-track lecturers.

Indeed, Xu and Solanki (2020) found no difference in student outcomes within first-quarter courses taught by TP/PoTs, tenure-track research-focused faculty, and non-tenure-track lecturers when comparing grades and enrollment in subsequent STEM courses. Individuals, regardless of structural roles and positions, can have the agency to implement specific instructional practices in their classrooms (Reinholz & Apkarian, 2018). Even within the TP/PoT position, individuals have a variety of training related to teaching and education, and they also pursue different forms of scholarly activity in STEM education (Harlow et al., 2020), suggesting a certain level of heterogeneity.

The variations in the number of TP/PoTs across departments and campuses (Harlow et al., 2020) suggest different values in hiring these individuals and utilizing the position as a structural element in undergraduate STEM education. Furthermore, the campuses in this study have a variety of initiatives related to the implementation of active learning. Together, these differences in resources represent different combinations of artifacts, knowledge, and values at the institutional level.

### Instructor and classroom characteristics

Individual agency may manifest as variations in individuals within the same structural element implementing more or less active-learning strategies, which we will examine through various instructor and classroom characteristics. For example, rank and years of teaching contribute to power dynamics within a department (Reinholz & Apkarian, 2018), which may result in different teaching assignments (e.g., smaller class size, courses more directly related to an individual’s expertise, etc.) that could facilitate the implementation of active-learning strategies in the classroom. We examine instructor characteristics, (faculty rank, years of teaching experience, and gender) and course characteristics (campus, discipline, and class size), that may influence the implementation of active-learning strategies in our STEM classrooms. Out of all of these factors, years of teaching experience (Alkhouri et al., 2021; Apkarian et al., 2021; Ebert-May et al., 2011; Emery et al., 2020; Lund et al., 2015) and class size (Alkhouri et al., 2021; Apkarian et al., 2021; Budd et al., 2013; Ebert-May et al., 2011; Emery et al., 2020; Henderson & Dancy, 2007; Smith et al., 2014; Stains et al., 2018) have both been shown to be the most significant and consistent predictors of implementation of active-learning strategies. Previous work has shown that the more teaching experience an instructor has with active learning, the more likely they are to implement it (Ebert-May et al., 2011). And that large class sizes can hinder the use of active learning with very large classes (100 or more students) self-reporting significantly more lecturing than instructors in other classes (Apkarian et al., 2021).

In contrast, there is evidence of differences in implementation of active learning across faculty rank (Emery et al., 2020; Lane et al., 2019), gender (Budd et al., 2013; Lane et al., 2019), campus or institution (Budd et al., 2013), and department or discipline (Alkhouri et al., 2021; Eagan, 2016; Ebert-May et al., 2011; Henderson & Dancy, 2007; Lund et al., 2015; Stains et al., 2018), but it is less well understood and/or results are inconsistent across studies. For example, when looking at usage of active-learning strategies by faculty rank and gender, faculty rank did not make a difference, but gender did make a difference (Lane et al., 2019). However, others found differences due to instructor’s gender with respect to teaching approaches over time (Emery et al., 2020). When considering campuses and departments, there were differences in teaching practices between instructors at research versus non-research universities (Budd et al., 2013). As a result, the impacts of these characteristics are worth further consideration in relation to implementation of active-learning strategies.

### COPUS

COPUS is a segmented observation protocol (Smith et al., 2013), where the class session is divided into short periods (e.g., 2-min time intervals) and the observer rates each item as it occurred in that time period. The COPUS instrument consists of 25 distinct codes that classify student and instructor behaviors (Tables 1 and 2) recorded in 2-min intervals by observers (Smith et al., 2013). There are many different ways that researchers choose to group the COPUS codes: (1) the 25 “original” COPUS codes (Smith et al., 2013), (2) the subset of eight “analyzer” codes out of the original 25 (Smith et al., 2018), (3) the eight “collapsed” categories consisting of all 25 original codes (Smith et al., 2014). In addition, we will consider a “novel” grouping of codes that we developed to differentiate learning activities. The description of the codes are displayed in Tables 1, 2. For the student COPUS codes, we distinguish between individual COPUS codes (“original” and “analyzer” codes) and combined codes (“collapsed” and “novel” codes) by using “Student.code” versus “S. code”. Similarly for the instructor COPUS codes we designate the individual codes using “Instructor.code” (“original” and “analyzer” codes), whereas combined codes are designated with “I. code” (“collapsed” and “novel” codes). The percent of class time spent on a particular code is found by taking the percent of 2-min intervals that contained the particular code. For the combined codes, we check to see if any code in the group occurred within a 2-min interval and then calculate the percent of 2-min intervals that contained any code in the group.

The 25 “original” COPUS codes focus on what the students are doing and what the instructor is doing. The eight “analyzer” codes have been used to characterize three groups of instructional styles (Stains et al., 2018): (1) didactic, classes with more than 80% of the class period including Instructor.Lec; (2) interactive lecture, classes in which instructors supplemented lecturing with other group activities or clicker questions with group work; and (3) student-centered, classes in which even larger portions of the class period were dedicated to group activities relative to the interactive style. The “collapsed” codes including both instructor and student behaviors (Smith et al., 2014).

The “collapsed” codes that are considered more teacher-centered and traditional are instructor lecturing, instructor writing on the board, instructor performing a demonstration or simulation, and students listening to the instructor (i.e., I.Presenting and S.Receiving). The more student-centered and active codes represented in the “collapsed” codes are student talking (S.Talking) and working (S.Working) as well as instructor guiding (I.Guiding). S.Talking includes students asking and answering questions, students engaged in a whole class discussion, and students presenting or watching student presentations. S.Working is used for individual thinking and problem solving, discussing clicker questions, working on a worksheet, making a prediction, or doing other assigned group activities. I.Guiding includes instructors posing or following up on clicker questions, listening and answering student questions, and moving through the class. The additional “collapsed” codes are less student-centered; students listening to instructor/taking notes (S.Receiving), students waiting or student other (S.Other) as well as instructors presenting, administration, and other (I.Presenting, I.Administration, I.Other). The “novel” codes are based on the level of interactions and presumed cognitive engagement in the classroom: facilitating interactive dialogues among students (S.Interactive or I.Interactive), promoting individual thinking in all students (S.Thinking or I.Thinking), attending to one or few students (S.Few or I.Few), providing information with minimal interactions (S.Minimal or I.Minimal), and other (S.Other or I.Miscellaneous). S.Other in the “novel” codes is the same as S.Other in the “collapsed” codes, whereas I.Miscellaneous in the “novel” codes combines I.Other and I.Administration from the “collapsed” codes (same as combining Instructor.Adm, Instructor.W, and Instructor.Other from the “original” codes).

**Table 1:** Student COPUS codes

Student COPUS code description	Student.Codes		S.Codes	
	Original	Analyzer	Collapsed	Novel
Listening: Listening to instructor/taking notes, etc.	L	–	Receiving	Minimal
Answer Question: Student answering a question posed by the instructor with rest of class listening	AnQ	–	Talking	Few
Asking: Student asks question	SQ	SQ	Talking	Few
Whole Class: Engaged in whole class discussion by offering explanations, opinion, judgment, etc. to whole class, often facilitated by instructor	WC	–	Talking	Interactive
Presentation: Presentation by student(s)	SP	–	Talking	Few
Thinking: Individual thinking/problem solving. Only mark when an instructor explicitly asks students to think about a clicker question or another question/problem on their own.	Ind	–	Working	Thinking
Clicker: Discuss clicker question in groups of 2 or more students	CG	CG	Working	Interactive
Worksheet: Working in groups on worksheet activity	WG	WG	Working	Interactive
Other Group: Other assigned group activity, such as responding to instructor question	OG	OG	Working	Interactive
Prediction: Making a prediction about the outcome of demo or experiment	Prd	–	Working	Thinking
Test/Quiz: Test or quiz	TQ	–	Working	Thinking
Waiting: Waiting (instructor late, working on fixing AV problems, instructor otherwise occupied, etc.)	W	–	Other	Other
Other: Other—explain in comments	Other	–	Other	Other
Total number of codes:	13	4	4	5

Descriptions of the “original” codes in Smith et al. (Smith et al., 2013), “analyzer” codes in Stains et al. (Smith et al. 2018), “collapsed” codes in Smith et al. (Smith et al. 2014), and “novel” codes. There are 19 unique student COPUS codes

**Table 2:** Instructor COPUS codes

Instructor COPUS code description	Instructor.Codes		I.Codes	
	Original	Analyzer	Collapsed	Novel
Lecturing: Lecturing (presenting content, deriving mathematical results, presenting a problem solution, etc.)	Lec	Lec	Presenting	Minimal
Writing: Real-time writing on board, doc. projector, etc. (often checked off along with Lec)	RtW	–	Presenting	Minimal
Demo/Video: Showing or conducting a demo, experiment, simulation, video, or animation	DV	–	Presenting	Minimal
Follow Up: Follow-up/feedback on clicker question or activity to entire class	FUp	–	Guiding	Few
Pose Question: Posing non-clicker question to students (non-rhetorical)	PQ	PQ	Guiding	Thinking
Clicker Question: Asking a clicker question (mark the entire time the instructor is using a clicker question, not just when first asked)	CQ	CQ	Guiding	Thinking
Answer Question: Listening to and answering student questions with entire class listening	AnQ	–	Guiding	Few
Moving/Guiding: Moving through class guiding ongoing student work during active learning task	MG	–	Guiding	Interactive
One on One: One-on-one extended discussion with one or a few individuals, not paying attention to the rest of the class (can be along with MG or AnQ)	1o1	1o1	Guiding	Interactive
Administration: Administration (assign homework, return tests, etc.)	Adm	–	Administration	Miscellaneous
Waiting: Waiting when there is an opportunity for an instructor to be interacting with or observing/listening to student or group activities and the instructor is not doing so	W	–	Other	Miscellaneous
Other: Other—explain in comments	Other	–	Other	Miscellaneous
Total number of codes	12	4	4	5

Descriptions of the “original” codes in Smith et al. (Smith et al. 2013), “analyzer” codes in Stains et al. (Smith et al. 2018), “collapsed” codes in Smith et al. (Smith et al. 2014), and “novel” codes. There are 19 unique instructor COPUS codes

## Methods

This study was approved by the Institutional Review Board at each of the three study campuses within the UC system (UC Irvine 2018-4211, UC Merced 2020-3, and UC San Diego 191318XX).

### Study context

UC is a research-intensive university system that enrolls over 285,500 full-time undergraduate students annually. The student body in the UC system is highly diverse, with most campuses designated as Hispanic-Serving Institutions. As a research-intensive public university system, UC exhibits many of the hallmarks of their peer institutions, including rising course enrollment and faculty promotion relying primarily on research productivity and external grant funding for tenure-track research-focused faculty (Brownell & Tanner, 2012). At the same time, the UC system has the novel TP/PoT position with a stronger emphasis on teaching as well as more the traditional non-tenure-track lecturer position. Each UC campus also has its own local culture and initiatives related to undergraduate STEM education. Thus, campuses within the UC system provide a unique and informative venue for examining the implementation of active learning in STEM courses in the context of faculty type and other instructor and classroom characteristics.

Campuses 1, 2, and 3 are similar, in that they are research-intensive institutions, have large student populations (roughly 10,000 undergraduates or greater), and all serve significant populations (25% +) of racially or ethnically minoritized students. All three campuses also have dedicated teaching and learning centers that offer professional development opportunities for instructors to implement evidence-based teaching practices. Nonetheless, Campus 3 is distinct in that it is home to an 8-session professional development series specifically aimed at the implementation of active learning pedagogies, which while voluntary has been completed by roughly 10% of the campus’ faculty. It also has the most number of initiatives to support evidence-based instructional practices, including a campus-wide education research initiative focused on undergraduate education, along with a newly completed active-learning building that exclusively contains classrooms designed to facilitate active learning.

### Data collection

Live COPUS observations were conducted in 125 STEM undergraduate courses across the three study campuses (Table 4). We observed each participating course at least twice for the entire duration of each class period, and at least two observers were present for each live observation. COPUS does not require observers to make judgments regarding teaching quality, but rather categorizes classroom activities by “what the students are doing” and “what the instructor is doing” (Smith et al., 2013). COPUS allows observers, after 1.5 hours of training (Smith et al., 2013), to reliably characterize behaviors in STEM classrooms by documenting 13 student behaviors (such as listening or answering questions) and 12 instructor behaviors (such as lecturing or posing questions) over 2-min time intervals (Denaro et al., 2021; Smith et al., 2013).

COPUS data collection and training was performed as established (Smith et al., 2013). All observers were trained at their home campus by faculty, postdoctoral scholars, and/or staff. Each campus had 5-15 trained observers conducting live COPUS observations. Observers were trained for a minimum of three hours; training included the description of the COPUS codes, presentation of classroom videos that observers used to practice coding with COPUS, and post observation mentoring and discussions. At Campus 3, training also included hands-on time with the Generalized Observation and Reflection Platform (GORP) (Martinez, 2018). Trained observers had initial reliability between the two-raters of at least 90% at two campuses and 66% at the remaining campus. At the campus with the lower initial reliability, at least two coders were present in the classroom for live observations to ensure trustworthiness in the data collection. In addition, any differences in coding were resolved through discussion to resolve any coding disagreements until reaching 100% consensus.

Instructors agreed at the beginning of each academic term to be observed during two class periods. Dates were assigned based on observer availability without any prior knowledge of the planned class activities. At Campus 2 and 3, observations were rescheduled if the originally selected date was an exam day; at Campus 1, exam dates were avoided based on syllabi provided by instructors. Observers coded classroom activities using COPUS for each class period and then summarized the data as percent of 2-min intervals during which a given code was occurring. For each class session observed, we used five datasets that are comprised of different subsets or combinations of codes. Dataset 1 includes the 25 “original” COPUS codes, dataset 2 includes the 8 “analyzer” codes, dataset 3 includes the 8 “collapsed” codes, and dataset 4 includes 10 “novel” codes. Dataset 5 includes all of the 38 “unique” codes from the first 4 datasets. Data for each course were averaged prior to data analysis.

We collected data on other instructor characteristics (faculty rank, years of teaching, and gender) and classroom characteristics (campus, discipline, and class size). For non-tenure-track lecturers, we assigned the rank of “associate” to continuing lecturers, who achieved that status after the equivalent of six years of full-time service with excellence in teaching based on performance review, and “assistant” to other lecturers. While the continuing lecturer status is not tenure, it is most equivalent to the promotion from assistant to associate rank in terms of time of service for TP/PoTs and tenure-track research-focused faculty. There is no equivalent promotion to the full professor rank for non-tenure-track lecturers in the UC system.

### Statistical analyses

#### Algorithms for clustering

Cluster analysis is an unsupervised learning technique which identifies groups of observations when there is no response variable of interest (Fisher, 1958; Hartigan & Wong, 1979; Hastie et al., 2009; Kaufman & Rousseeuw, 1987; MacQueen, 1967; Pollard, 1981). The choice of clustering algorithm or addition of new data can result in different clusters (Ben-David et al., 2006; Fisher, 1958; Hartigan, 1975; Hartigan & Wong, 1979; Hastie et al., 2001; James et al., 2013; Tibshirani & Walther, 2005). While Stains et al. (Stains et al., 2018) generated a COPUS Analyzer tool (http://www.copusprofiles.org/) to “automatically classif[y] classroom observations into specific instructional styles, called COPUS Profiles”, we previously showed that the cluster assignments vary when utilizing the COPUS Analyzer versus a de novo cluster analysis guided by the parameters established by the Analyzer (Denaro et al., 2021). Since clustering techniques are meant to be descriptive, rather than predictive, when new data are gathered a new clustering algorithm should be employed (Ben-David et al., 2006; Fisher, 1958; Hartigan, 1975; Hartigan & Wong, 1979; Hastie et al., 2001; James et al., 2013).

There are many choices of clustering algorithms that one can use to cluster heterogeneous data into homogeneous groups (Kaufman & Rousseeuw, 2008, 2009; Ng & Han, 1994). Rather than choose a single algorithm, we considered 11 different types of cluster analyses (*k*-means, partitioning around medoids [PAM], non-negative matrix factorization using euclidean distance, hierarchical clustering, divisive analysis clustering, affinity propagation, spectral clustering using radial-basis kernel function, Gaussian mixture model, self-organizing map with hierarchical clustering, fuzzy *C*-means clustering, and hierarchical density-based spatial clustering of applications with noise) and evaluated which one fit our data best. To specify the desired number of clusters, *k*, the *diceR* package in R was used (Chiu & Talhouk, 2018). For each algorithm and every value of *k*, a random subsampling of 80% of the original observations is carried out 5 times. Therefore not every sample is included in each clustering. The clustering for each of the 11 algorithms is completed using *k*-nearest neighbor and majority voting. The relevant number of clusters was found by evaluating 15 different internal indices (see the supplemental materials for a complete list, Table S1) while varying the cluster size (from $k = 2, \dots , 9$). For further discussion of the indices, see Charrad et al. (2014) and Chiu and Talhouk (2018). The internal clustering criteria consist of measures of compactness (how similar are objects within the same cluster), separation (how distinct are objects from different clusters), and robustness (how reproducible are the clusters in other datasets). Index citations and whether or not the specific index should be maximized or minimized are included in the supplemental materials (Additional file 1: Table S1).

#### Ensemble of algorithms

Furthermore, instead of relying on a single “best” clustering, we use an ensemble of algorithms applied to our data. To create the ensemble, we run multiple clusterings using different subsets of the COPUS codes (“original”, “analyzer”, “collapsed”, “novel”, and “unique”) and then combine the information of the respective individual algorithms. Use of the ensemble of algorithms gives us a robust cluster assignment, as our cluster assignment does not rely on a single choice of variables, nor does it rely on a single choice for determining the best number of clusters, nor does it rely on a single choice of consensus function. It has been shown that for classification an ensemble average will perform better than a single classifier (Moon et al., 2007). A few applications of ensemble algorithms can be found in the educational literature (Beemer et al., 2018; Kotsiantis et al., 2010; Pardos et al., 2012).

**Fig. 1:**
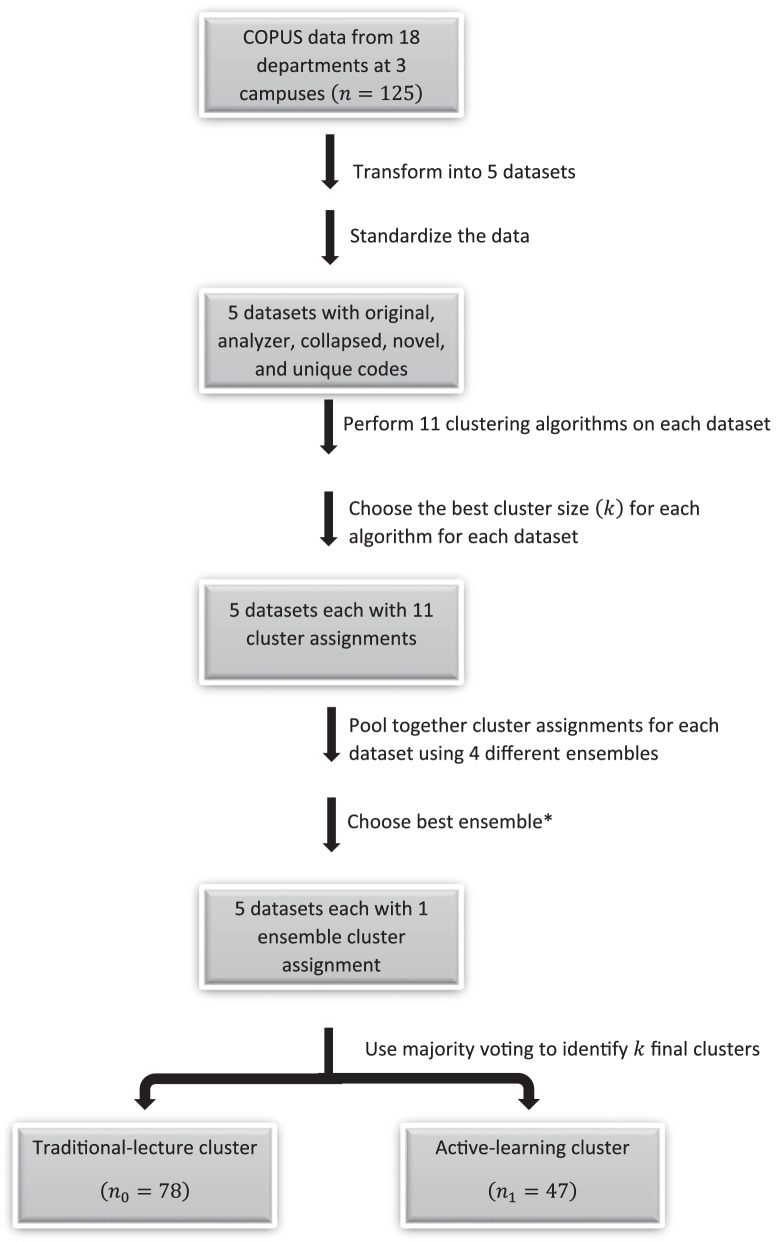
Robust ensemble clustering process

Figure 1 displays the algorithm that we used to obtain our final clusters. We have COPUS data from $n = 125$ undergraduate courses across 18 STEM departments at 3 campuses. We then transformed our original COPUS data into 5 datasets (original, analyzer, collapsed, novel, and unique). All COPUS codes were standardized to have a mean of 0 and a standard deviation of 1 prior to clustering. We combined the results of the individual clustering algorithms (*k*-means, PAM, etc.) using a consensus function. The consensus function is used to combine the clustering results of the algorithms to create an ensemble. Next, we considered 4 different ways to combine the clustering results: *k*-modes (Huang, 1997), majority voting (Ayad & Kamel, 2010), Cluster-based Similarity Partitioning Algorithm (CSPA) (Strehl & Ghosh, 2002; Ghosh & Acharya, 2011), and Linkage Clustering Ensemble (LCE) (Iam-On et al., 2010; Iam-on & Garrett, 2010). After creating the cluster ensembles, we evaluated whether or not the individual algorithms or the ensembles created the best clusters using the internal indices previously described and by having well balanced cluster sizes. Using majority voting, the robust ensemble clustering process identifies the final clusters. We note that the number of final clusters was not predetermined.

#### Logistic regression

To present evidence of instructor (faculty type, faculty rank, years of teaching, and gender) and classroom (campus, discipline, and class size) characteristics that correlate with classes within the active-learning cluster(s), logistic regression was used. We modeled the odds of a course falling into one of two groups (in this case being classified as low- or high-active learning based on cluster assignment) to address our specific research questions. More specifically, we want to know if there is an increase in the odds of teaching an active-learning course for certain course or instructor characteristics compared to teaching a traditional lecture (where the instructor is doing most of the talking while the students are primarily listening). To accomplish this, we fit a logistic regression model utilizing the *stats* package in R (R Core Team, 2019). Assuming we have a sample of *n* independent observations, ($x_i$ , $y_i$), we obtain estimates for $\beta ^t =(\beta _0, \beta _1, \dots , \beta _k)$. Let $x^t =(x_1, x_2, \dots , x_k)$ be the *k* predictors: tenure-track research faculty, tenure-track teaching faculty, or non-tenure track lecturers; assistant, associate, or full rank professor; small (fewer than 100 students), medium (100-199), or large (200 or greater students) class size; Biological Sciences, Physical Sciences, Information and Computer Sciences (I &C Sciences), or Engineering; study campus; and gender of the instructor. Let *Y* be whether or not the classroom observation falls under the active-learning cluster(s) and the probability of the classroom observation being part of the active-learning cluster(s) be $p= P(Y = 1)$. We assume a linear relationship between the predictor variables and the log-odds of the event that the classroom observation falls into the active-learning cluster(s). The model is given by:

1 $$\begin{aligned} log\left( \dfrac{p}{1-p}\right) = \beta _0 + \beta _1x_1 + \dots + \beta _kx_k \end{aligned}.$$

First, we built a full model where we include instructor (faculty type, faculty rank, years of teaching, and gender) and classroom (campus, discipline, and class size) characteristics. We performed best subsets logistic regression using the *bestglm* package in R McLeod and Xu (2018) to choose the best fitting model to the data. The best subsets procedure entails building a model of the log odds of active-learning cluster(s) for each of the possible subsets of covariates and calculating the respective Akaike Information Criteria (AIC) of the model. The final model is chosen by minimizing the AIC. The AIC balances model fit with generalizability Chakrabarti and Ghosh (2011); Sakamoto et al. (1986). We checked for significant 2-way interactions between faculty type and the remaining predictors of the active-learning cluster(s).

## Results

Summary statistics of the raw percentage of time spent on each code split by faculty type can be found in Table 3. The corresponding standardized percentage of time spent on each code can be found in the supplemental materials. The most common codes are student listening (Student.L) and instructor lecturing (Instructor.Lec). Students spent less than 5% of class time on each of the following activities: engaging in a whole class discussion (Student.WC), giving or watching student presentations (Student.SP), making predictions about an outcome of a demonstration or experiment (Student.Prd), taking a test or quiz (Student.TQ), waiting for the instructor (Student.W), discussing clicker questions (Student.CG), working in groups (Student.WG), and other activities (Student.O). Instructors spent less than 5% of class time on each of the following activities: showing or conducting a demo, experiment, or simulation (Instructor.DV), one-on-one extended discussion with one or a few students (Instructor.1o1), waiting to interact with student when given the opportunity (Instructor.W), and other activities (Instructor.O). 5 of the 8 “analyzer” codes were rarely seen for faculty members (Student.CG, Student.WG, and the instructor asking a clicker question [Instructor.CQ]). In addition, 2 of the remaining 5 “analyzer” codes were rare for tenure-track research faculty and non-tenure track lecturers (Student.OG and Instructor.1o1), but were used more often by the tenure-track teaching faculty.

**Table 3:** Summary statistics of the percentage of time spent on each of the COPUS codes by faculty type

Dataset	Code	Tenure-track			*F*	*p*-value
		Yes	Yes	No		
		Teaching faculty	Research faculty	Lecturers		
1,3,4,5	Student.L	80.85 (16.37)	95.37 (6.59)	93.38 (11.29)	5.04	0.01*
	S.Receiving/					
	S.Minimal					
1,5	Student.AnQ	18.45 (18.31)	6.52 (12.65)	14.41 (22.27)	2.14	0.12
1,5	Student.WC	0.00 (0.97)	0.00 (0.49)	0.00 (1.03)	1.00	0.37
1,5	Student.SP	0.00 (0.00)	0.00 (0.00)	0.00 (0.00)	–	–
1,5	Student.Ind	12.42 (15.69)	1.96 (5.99)	4.00 (11.52)	9.79	$<0.001$*
1,5	Student.Prd	0.00 (0.00)	0.00 (0.00)	0.00 (0.00)	–	–
1,5	Student.TQ	0.00 (0.00)	0.00 (0.00)	0.00 (0.00)	–	–
1,5	Student.W	0.00 (1.57)	1.92 (3.10)	0.64 (3.10)	2.20	0.12
1,5	Student.O	0.00 (1.58)	0.00 (1.68)	0.00 (1.06)	1.00	0.37
1,2,5	Student.SQ	10.16 (11.58)	6.74 (8.15)	6.33 (7.86)	1.55	0.22
1,2,5	Student.CG	1.93 (11.26)	0.00 (1.87)	0.00 (9.86)	1.75	0.18
1,2,5	Student.WG	1.28 (7.10)	0.00 (1.54)	0.00 (0.00)	1.96	0.14
1,2,5	Student.OG	5.85 (12.60)	0.00 (1.01)	0.00 (6.45)	5.87	< 0.001*
3,5	S.Working	39.48 (24.26)	7.11 (22.62)	20.30 (23.95)	14.50	< 0.001*
3,5	S.Talking	27.77 (24.66)	16.27 (21.31)	23.77 (19.48)	2.47	0.09
3,4,5	S.Other	1.92 (3.61)	2.96 (3.02)	1.96 (4.32)	0.17	0.85
4,5	S.Interactive	26.32 (27.41)	2.94 (16.46)	14.23 (20.96)	10.92	< 0.001*
4,5	S.Thinking	15.89 (18.61)	3.25 (8.55)	4.56 (12.25)	9.46	< 0.001*
4,5	S.Few	27.11 (21.83)	16.07 (21.72)	22.39 (19.48)	2.07	0.13
1,5	Instructor.RtW	12.34 (27.91)	11.13 (59.29)	25.85 (36.48)	1.30	0.28
1,5	Instructor.DV	1.28 (5.12)	1.25 (6.75)	2.44 (6.76)	0.15	0.86
1,5	Instructor.FUp	21.32 (20.55)	10.58 (13.92)	17.88 (20.09)	5.89	< 0.001*
1,5	Instructor.AnQ	10.53 (12.11)	6.64 (8.69)	6.92 (7.78)	1.66	0.20
1,5	Instructor.MG	11.84 (24.43)	0.00 (5.76)	0.00 (13.72)	11.32	< 0.001*
1,3,5	Instructor.Adm/	8.81 (7.77)	6.68 (8.24)	7.69 (7.33)	1.92	0.15
	I.Administration					
1,5	Instructor.W	0.64 (3.29)	0.61 (3.10)	0.64 (3.22)	0.02	0.98
1,5	Instructor.O	0.81 (4.39)	0.00 (2.96)	0.00 (1.83)	0.58	0.56
1,2,5	Instructor.Lec	54.70 (28.58)	84.46 (21.09)	70.54 (25.90)	7.96	< 0.001*
1,2,5	Instructor.PQ	20.86 (18.09)	8.18 (17.77)	18.71 (20.96)	1.83	0.16
1,2,5	Instructor.CQ	6.00 (15.92)	0.00 (7.53)	0.00 (12.86)	2.33	0.10
1,2,5	Instructor.1o1	2.56 (6.32)	0.00 (0.00)	0.00 (3.27)	5.78	< 0.001*
3,4,5	I.Presenting/	67.00 (24.96)	87.75 (14.53)	80.57 (18.22)	6.24	< 0.001*
	I.Minimal					
3,5	I.Guiding	70.67 (23.82)	36.41 (37.26)	50.65 (23.71)	14.26	< 0.001*
3,5	I.Other	3.66 (7.81)	2.04 (6.31)	3.28 (6.49)	0.17	0.84
4,5	I.Interactive	12.89 (23.67)	0.00 (6.63)	0.67 (13.79)	11.68	< 0.001*
4,5	I.Thinking	36.87 (22.73)	16.8 (24.73)	31.3 (24.19)	4.27	0.02
4,5	I.Few	33.75 (18.54)	20.83 (20.09)	23.72 (19.95)	7.46	< 0.001*
4,5	I.Miscellaneous	14.00 (15.00)	8.21 (11.79)	11.80 (13.59)	0.81	0.45

Mean and standard deviation (in parentheses) are given as well as the* F* statistic and* p*-value for testing if there is a difference in the amount of time spent on a code across the three faculty types. Significance is denoted for codes using a Bonferroni correction of $\alpha ^* = 0.05/38 = 0.0013$

We found that TP/PoTs, tenure-track research-focused faculty, and non-tenure-track lecturers differ in what they do in the classroom and how often they implement active-learning strategies. Significance is denoted for codes using a Bonferroni correction of $\alpha ^* = 0.05/38 = 0.0013$. There are different amounts of instructor lecturing (Instructor.Lec), presenting (I.Presenting), follow-up (Instructor.FUp), moving and guiding (Instructor.MG and I.Guiding), one-on-one extended discussion (Instructor.1o1), interactive (I.Interactive), active (I.Active), and passive (I.Passive) for TP/PoTs, tenure-track research-focused faculty, and non-tenure-track lecturers. Correspondingly, in student behaviors, there are different amounts of student thinking (Student.Ind), group activities (Student.OG), working (S.Working), interactive (S.Interactive), constructive (S.Constructive), and passive (S.Passive).

### RQ1: to what extent are TP/PoTs more likely to implement active-learning strategies compared to non-tenure track lecturers and tenure-track research faculty?

The COPUS data separated into two clusters representing traditional lecture and active learning (based on majority voting and the robust ensemble clustering process displayed in Fig. 1). Details of the clustering algorithm can be found in the supplemental materials (Additional file 1: Figs. S1–S53, Tables S3–S19). We note that the number of clusters was not predetermined, however our data resulted in two final cluster assignments. As a reference, the instructor and classroom characteristics for the individual clustering ensembles of the five datasets (original, analyzer, collapsed, novel, and unique codes) can be found in the supplemental materials (Additional file 1: Tables S20–S26). The instructor and classroom characteristics vary across the traditional-lecture cluster ($n_0 = 78$) and the active-learning cluster ($n_1 = 47$) (Table 4). For example, in the traditional-lecture cluster, tenure-track research-focused faculty represent the largest proportion at 50%, followed by non-tenure-track lecturers at 28% and TP/PoTs at 22%. In contrast, in the active-learning cluster, TP/PoTs represent the largest proportion at 47%, followed by tenure-track research-focused faculty at 28% and non-tenure-track lecturers at 26%.

**Table 4:** Summary statistics for the final clustering

Variable	Traditional cluster	Active cluster	All classes
Faculty type			
Teaching faculty	17 (22%)	22 (47%)	39 (31%)
Research faculty	39 (50%)	13 (28%)	52 (42%)
Lecturers	22 (28%)	12 (26%)	34 (27%)
Faculty rank			
Assistant	32 (41%)	28 (60%)	60 (48%)
Associate	17 (22%)	10 (21%)	27 (22%)
Full	29 (37%)	9 (19%)	38 (30%)
Years of teaching	9 (6)	9 (7)	9 (6)
Gender			
Female	33 (42%)	26 (55%)	59 (47%)
Non-female	45 (58%)	21 (45%)	66 (53%)
Campus			
1	4 (5%)	11 (23%)	15 (12%)
2	18 (23%)	3 (6%)	21 (17%)
3	56 (72%)	33 (70%)	89 (71%)
Discipline			
Biological Sciences	15 (19%)	24 (51%)	39 (31%)
Physical Sciences	30 (38%)	6 (13%)	36 (29%)
I &C Sciences	15 (19%)	11 (23%)	26 (21%)
Engineering	18 (23%)	6 (13%)	24 (19%)
Class size			
Small (0–99)	16 (21%)	17 (36%)	33 (26%)
Medium (100–199)	34 (44%)	9 (19%)	43 (34%)
Large (200 +)	28 (36%)	21 (45%)	49 (39%)
	$n_0$ = 78	$n_1$ = 47	*n* = 125

Summary of instructor and classroom demographics for the traditional-lecture and active-learning clusters. The number and the conditional percent (given cluster) are presented in parentheses for categorical variables. The mean and standard deviation (in parentheses) are presented for the quantitative variable

The summary statistics of each of the COPUS codes by final cluster assignment (Table 5) reveal that there is a significant difference in what the students and instructors are doing for those in the traditional-lecture cluster and those in the active-learning cluster for the majority of codes. For example, instructors in the traditional-lecture cluster spend more time lecturing (Instructor.Lec in original and analyzer codes) compared to faculty in the active-learning cluster (87% versus 47% of the 2-min intervals). Instructors in the traditional-lecture cluster also spend less time moving through class guiding ongoing student work during active-learning tasks (Instructor.MG in original codes, 0% versus 17%). Correspondingly, students in the traditional-lecture classrooms spend more time listening (Student.L in original codes, 96% versus 78%) and less time engaging in group work (Student.OG in original and analyzer codes, 0% versus 12%). For the collapsed and novel codes, almost all codes show significant differences between the traditional-lecture cluster and active-learning cluster (Table 5). The boxplots for each of the codes split by final cluster assignment are included in the supplemental materials (Additional file 1: Fig. S1–S38).

**Table 5:** Summary statistics of the percentage of time for each of the COPUS codes by cluster

Dataset	Code	Traditional	Active	*F*	*p*-value
		Cluster	Cluster		
1,3,4,5	Student.L	96.45 (5.60)	77.9 (19.22)	97.94	$<0.001$*
	S.Receiving/				
	S.Minimal				
1,5	Student.AnQ	7.32 (14.56)	14.62 (15.99)	1.17	0.28
1,5	Student.WC	0.00 (0.00)	0.00 (1.06)	4.31	0.04
1,5	Student.SP	0.00 (0.00)	0.00 (0.00)	–	–
1,5	Student.Ind	2.00 (8.06)	7.45 (14.86)	17.11	$<0.001$*
1,5	Student.Prd	0.00 (0.00)	0.00 (0.00)	–	–
1,5	Student.TQ	0.00 (0.00)	0.00 (0.00)	–	–
1,5	Student.W	0.00 (1.83)	1.96 (3.25)	9.26	$<0.001$*
1,5	Student.O	0.00 (1.55)	0.00 (2.00)	0.02	0.88
1,2,5	Student.SQ	8.53 (11.67)	7.70 (6.54)	0.41	0.52
1,2,5	Student.CG	0.00 (1.55)	2.40 (12.68)	19.52	$<0.001$*
1,2,5	Student.WG	0.00 (0.00)	1.93 (6.82)	6.45	0.01
1,2,5	Student.OG	0.00 (0.00)	12.00 (16.02)	65.69	$<0.001$*
3,5	S.Working	5.88 (14.97)	43.48 (18.76)	153.94	$<0.001$*
3,5	S.Talking	18.62 (23.94)	26.8 (20.17)	1.18	0.28
3,4,5	S.Other	1.96 (3.31)	3.85 (5.10)	1.77	0.19
4,5	S.Interactive	2.32 (10.16)	34.62 (17.32)	150.15	$<0.001$*
4,5	S.Thinking	2.39 (10.01)	16.59 (18.63)	29.36	$<0.001$*
4,5	S.Few	18.4 (23.94)	26.09 (18.22)	0.63	0.43
1,5	Instructor.RtW	29.57 (57.35)	5.5 (13.43)	21.12	$<0.001$*
1,5	Instructor.DV	1.52 (6.39)	1.92 (7.33)	0.13	0.72
1,5	Instructor.FUp	9.27 (14.57)	30.91 (20.42)	58.34	$<0.001$*
1,5	Instructor.AnQ	8.55 (12.06)	7.42 (8.11)	0.22	0.64
1,5	Instructor.MG	0.00 (1.61)	17.07 (18.83)	55.99	$<0.001$*
1,3,5	Instructor.Adm/	4.74 (5.17)	13.69 (9.47)	61.38	$<0.001$*
	I.Administration				
1,5	Instructor.W	0.00 (1.68)	3.92 (7.47)	22.79	$<0.001$*
1,5	Instructor.O	0.00 (1.55)	1.66 (5.98)	13.95	$<0.001$*
1,2,5	Instructor.Lec	87.73 (13.57)	47.08 (17.44)	189.11	$<0.001$*
1,2,5	Instructor.PQ	10.79 (19.04)	25.00 (17.12)	2.12	0.15
1,2,5	Instructor.CQ	0.00 (7.46)	9.49 (19.22)	17.63	$<0.001$*
1,2,5	Instructor.1o1	0.00 (0.00)	5.44 (8.56)	31.26	$<0.001$
3,4,5	I.Presenting/	90.35 (11.04)	55.85 (23.96)	181.05	$<0.001$*
	I.Minimal				
3,5	I.Guiding	37.39 (32.7)	72.75 (16.82)	50.66	$<0.001$*
3,5	I.Other	1.54 (3.85)	8.93 (14.03)	36.57	$<0.001$*
4,5	I.Interactive	0.00 (3.02)	20.00 (17.88)	61.10	$<0.001$*
4,5	I.Thinking	17.97 (24.26)	37.95 (14.53)	14.45	$<0.001$*
4,5	I.Few	19.04 (17.00)	38.47 (19.13)	37.46	$<0.001$
4,5	I.Miscellaneous	6.72 (7.71)	23.64 (13.11)	91.42	$<0.001$*

Mean and standard deviation (in parentheses) are given as well as the* F* statistic and* p*-value for testing if there is a difference in the amount of time spent on a code for the traditional and active cluster. Significance is denoted for codes using a Bonferroni correction of $\alpha ^* = 0.05/38 = 0.0013$

While examining the individual codes helps us consider the impact of an individual code, many of the COPUS codes overlap and are not independent of one another. For this reason, we used robust cluster ensemble methods to obtain a cluster assignment for each course (active-learning and traditional-lecture cluster). Rather than conducting analyses on the individual codes, we modeled the likelihood of an instructor falling within a certain cluster, i.e., being classified as traditional lecture or active learning, after accounting for other instructor and classroom variables. The odds of being in the active-learning cluster compared to the traditional-lecture cluster are presented in (Tables 6, 7, 8). In the context of interpreting the odds ratios of the logistic regression model, all other variables in the model are assumed to be held constant. Table 6 presents the results of the logistic regression models with all of our instructor variables (faculty type, faculty rank, years of teaching, gender) and classroom variables (campus, discipline, and class size) as inputs and the odds of being in the active-learning cluster (based on the final cluster assignment) as the response (see Additional file 1 for alternative models, Tables S27–S31). The logistic regression model with all of our instructor (faculty type, faculty rank, years of teaching, and gender) and classroom (campus, discipline, and class size) characteristics as well as the 2-way interactions between faculty type and faculty rank, years of teaching, gender, discipline, and class size did not yield an improved model and can be found in Table 7. Table 8 displays the final model after using best subsets logistic regression (choosing the best model based on the AIC criterion) with the response as the odds of being in the active-learning cluster (based on the final cluster assignment) and all possible combinations and subsets of instructor and classroom characteristics as the inputs. There is no difference in the odds of an instructor falling in the active-learning cluster when comparing teaching faculty and non-tenure track lectures. However, we we see that TP/PoTs are more likely to be in the active-learning cluster compared to tenure-track research-focused faculty, with the odds being significantly less than one.

**Table 6:** Logistic regression model for active-learning cluster

	Estimated	95% confidence	Test	*p*-value
	Odds	Interval	Statistic	
Intercept	7.27	(1.15, 45.95)	2.11	0.04*
Faculty type				
RG: Teaching faculty				
Research faculty	0.28	(0.08, 0.93)	− 2.08	0.04*
Lecturers	0.46	(0.13, 1.60)	− 1.22	0.22
Faculty rank				
RG: Assistant				
Associate	0.53	(0.13, 2.12)	− 0.90	0.37
Full	0.72	(0.14, 3.78)	− 0.39	0.69
Years of teaching	1.03	(0.95, 1.13)	0.74	0.46
Gender				
RG: non-female				
Female	1.67	(0.60, 4.61)	0.98	0.33
Campus				
RG: Campus 3				
Campus 2	0.19	(0.04, 0.98)	− 1.98	0.05*
Campus 1	2.21	(0.39, 12.60)	0.89	0.37
Discipline				
RG: Biological Sciences				
Engineering	0.33	(0.07, 1.54)	− 1.41	0.16
I &C Sciences	0.59	(0.14, 2.50)	− 0.72	0.47
Physical sciences	0.12	(0.03, 0.52)	− 2.86	$<0.001$*
Class size				
RG: Small (0–99)				
Medium (100–199)	0.15	(0.04, 0.57)	− 2.80	0.01*
Large (200 +)	0.25	(0.08, 0.80)	− 2.33	0.02*
AIC $=$ 149.58				

The coefficients represent the increase/decrease in the odds of being in the active-learning cluster (based on the final cluster assignment) for each of the variables of interest (while holding the other variables in the model constant). The reference group (RG) are labeled for each of the categorical variables

**Table 7:** Logistic regression model for active-learning cluster with 2-way interactions

	Estimated	95% confidence	Test	*p*-value
	Odds	Interval	Statistic	
Intercept	12.86	(0.32, 520.67)	1.35	0.18
Faculty type				
RG: Teaching faculty				
Research faculty	0.65	(0.01, 75.30)	$-$ 0.18	0.86
Lecturers	0.06	(0.00, 6.19)	$-$ 1.18	0.24
Faculty rank				
RG: Assistant				
Associate	0.96	(0.10, 9.29)	$-$ 0.03	0.97
Full	1.66	(0.09, 31.54)	0.34	0.74
Years of teaching	1.03	(0.88, 1.21)	0.42	0.68
Gender				
RG: non-female				
Female	1.67	(0.60, 4.61)	0.98	0.33
Campus				
RG: Campus 3				
Campus 2	0.31	(0.05, 1.97)	$-$ 1.24	0.22
Campus 1	2.74	(0.28, 27.08)	0.86	0.39
Discipline				
RG: Biological Sciences				
Engineering	0.13	(0.00, 3.48)	$-$ 1.22	0.22
I &C Sciences	0.09	(0.01, 1.48)	$-$ 1.69	0.09
Physical sciences	0.07	(0.01, 0.90)	$-$ 2.05	0.04*
Class size				
RG: Small (0–99)				
Medium (100–199)	0.14	(0.01, 1.68)	$-$ 1.55	0.12
Large (200 +)	0.17	(0.02, 1.40)	$-$ 1.65	0.10
Interactions: Research Faculty and				
Associate	2.11	(0.03, 148.10)	0.34	0.73
Full	1.43	(0.02, 88.38)	0.17	0.86
Female	0.34	(0.02, 6.93)	$-$ 0.70	0.48
Years of teaching	0.87	(0.68, 1.11)	$-$ 1.13	0.26
Engineering	4.19	(0.08, 207.84)	0.72	0.47
I &C Sciences	18.40	(0.51, 658.40)	1.60	0.11
Physical sciences	0.98	(0.02, 49.87)	$-$ 0.01	0.99
Medium (100–199)	0.29	(0.01, 13.66)	$-$ 0.63	0.53
Large (200 +)	0.82	(0.04, 18.57)	$-$ 0.13	0.90
Interactions: Lecturers and				
Associate	0.04	(0.00, 8.82)	$-$ 1.16	0.25
Full	–	–	–	–
Female	1.93	(0.06, 58.23)	0.38	0.71
Years of teaching	1.17	(0.83, 1.64)	0.90	0.37
Engineering	2.73	(0.03, 274.25)	0.43	0.67
I &C Sciences	12.54	(0.34, 462.56)	1.37	0.17
Physical sciences	0.77	(0.01, 54.78)	$-$ 0.12	0.90
Medium (100–199)	2.03	(0.05, 80.35)	0.38	0.71
Large (200 +)	1.77	(0.07, 46.38)	0.34	0.73
AIC $=$ 172.59				

The coefficients represent the increase/decrease in the odds of being in the active-learning cluster (based on the final cluster assignment) for each of the variables of interest (while holding the other variables in the model constant). The reference group (RG) are labeled for each of the categorical variables. The 2-way interactions between faculty type and instructor characteristics as well as faculty type and classroom characteristics are included in the model

**Table 8:** Final logistic regression model for active-learning cluster

	Estimated	95% confidence	Test	*p*-value
	Odds	Interval	Statistic	
Intercept	9.78	(2.19, 43.69)	2.99	$<0.001$*
Faculty type				
RG: Teaching faculty				
Research faculty	0.28	(0.10, 0.79)	$-$ 2.39	0.02*
Lecturers	0.47	(0.15, 1.50)	$-$ 1.28	0.20
Campus				
RG: Campus 3				
Campus 2	0.19	(0.04, 0.92)	$-$ 2.06	0.04*
Campus 1	2.56	(0.53, 12.42)	1.17	0.24
Discipline				
RG: Biological Sciences				
Engineering	0.29	(0.07, 1.23)	$-$ 1.67	0.09
I &C Sciences	0.56	(0.14, 2.24)	$-$ 0.81	0.42
Physical sciences	0.13	(0.03, 0.54)	$-$ 2.84	$<0.001$*
Class Size				
RG: Small (0–99)				
Medium (100–199)	0.14	(0.04, 0.51)	$-$ 2.98	$<0.001$*
Large (200 +)	0.26	(0.08, 0.82)	$-$ 2.30	0.02*
AIC $=$ 143.47				

The final model was found by using best subsets logistic regression to model the log odds of the active-learning cluster (based on the final cluster assignment) and all possible subsets of the instructor (faculty type, faculty rank, years of teaching, gender) and classroom (campus, discipline, and class size) characteristics. The coefficients represent the increase/decrease in the odds of being in the active-learning cluster for each of the variables of interest (while holding the other variables in the model constant). The reference group (RG) are labeled for each of the categorical variables

### RQ2: what instructor and classroom characteristics correlate with active-learning?

Not all of the instructor and classroom characteristics are significant in predicting whether or not a faculty member ended up in the active-learning cluster (Table 6). By minimizing the AIC, we obtained the final logistic regression model (Table 8). In the final model, campus, discipline, and class size are also associated with changes in the odds of being in the active-learning cluster compared to the traditional-lecture cluster in addition to faculty type. Campus 3 was more likely to have instructors who adopt active-learning strategies relative to Campus 2. Physical Sciences classes tend to have instructors who teach less actively compared to Biological Sciences. Smaller class sizes also tend to have instructors who teach more actively. These results potentially relate to how people and power are interconnected and are further elaborated on in the Discussion section.

## Discussion

Our findings show that TP/PoTs are more likely to be in the active-learning cluster (i.e., teach with more active-learning strategies) compared to tenure-track research-focused faculty. These findings are based on leveraging a robust clustering methodology of COPUS observations across 3 campuses and strongly support the hypothesis that the structure of the TP/PoT position makes a difference in the instructional practices being implemented in the classroom. In particular, TP/PoTs are more likely to spend class time moving and guiding students in active-learning tasks and have more one-on-one extended discussion with students. Consistent with the existing literature (Smith et al., 2013), these instructor behaviors correlate with students spending more time engaging in individual thinking and group activities.

This finding is unlikely to be merely the result of the TP/PoT position being teaching-intensive. Previous studies found that the proportion of an instructor’s academic appointment devoted to teaching positively correlates with the implementation of active-learning strategies (Ebert-May et al., 2011), whereas the level of research activity negatively correlates with the implementation of active-learning strategies (Apkarian et al., 2021). Such a direct correlation would imply that non-tenure-track lecturers should be most likely to implement active-learning strategies because 100% of their academic appointment is devoted to teaching. Instead, we found that TP/PoTs, who do less teaching and more research, are no more or less likely than non-tenure-track lecturers to be classified in the active-learning cluster.

It remains unclear what other factors contribute to TP/PoTs teaching more actively. Within the UC system, TP/PoTs as a structure differ from non-tenure-track lecturers by a number of important features. While we are not able to disentangle how these different factors may contribute to the implementation of active learning in our study context, our findings combined with previous research suggest which features may be most relevant. One feature is that TP/PoTs are tenure-track faculty and voting members of the Academic Senate (University of California Office of the President, 2018). While some might argue that the security of employment that comes with tenure could potentially allow TP/PoTs to use newer pedagogical methods such as active learning, neither previous research nor our results support that. A recent large-scale survey study found that security of employment (defined as “promotion that comes with increased security of employment”, which does not necessarily equal tenure) does not show a correlation with percentage of class time spent on lecturing (Apkarian et al., 2021). Another feature of TP/PoTs is that they are charged to engage in scholarship (e.g., DBER and curriculum development) and service that is often related to the educational mission of their department and campus (Harlow et al., 2020, 2021). The same survey study also found that exposure to education projects and active learning decreases self-reported time spent on lecturing in undergraduate STEM courses (Apkarian et al., 2021). Our results are consistent with a model in which TP/PoTs engage in DBER and evidence-based curriculum development, which exposes them to education projects and active learning through these professional activities, which influences them to use active-learning strategies. While our work suggests that TP/PoTs represent a potential means to increase implementation of active-learning strategies in undergraduate STEM education, more research is needed to identify which features of this position correlate best with teaching style.

Our results imply that individuals have the agency to implement active-learning strategies regardless of the structure of their position. Despite the result that TP/PoTs are more likely to be in the active-learning cluster, not all TP/PoTs are in the active-learning cluster. Similarly, not all tenure-track research-focused faculty and non-tenure-track lecturers are in the traditional-lecture cluster. Furthermore, consistent with existing literature (Stains et al., 2018), our findings suggest that most undergraduate STEM instructors are still teaching using traditional lecture-based instruction, and adoption of active-learning strategies remains low. Therefore, the structure of TP/PoT alone—or even coupled with the agency of individual people—is not sufficient for widespread implementation of evidence-based instructional practices.

In addition, we found that discipline, campus and class size increased the likelihood of an instructor being classified in the active-learning cluster, whereas faculty rank, years of teaching experience, and gender did not have such an impact. In contrast to our results, a previous study using the Reformed Teaching Observation Protocol (RTOP) found that years of teaching experience negatively correlated with the implementation of active-learning strategies (Ebert-May et al., 2011). Faculty rank and years of teaching experience can both indirectly represent power, and one might expect that these two characteristics should be correlated, i.e., people with more years of teaching experience being promoted through the faculty ranks. One might expect that faculty rank and years of teaching experience should be correlated, i.e., people with more years of teaching experience being promoted through the faculty ranks. While years of experience was similar when comparing the traditional and active cluster, we note that the majority of the active-learning cluster consisted of faculty at the Assistant Professor rank. Therefore, faculty ranks may represent changing expectations of the TP/PoT position in our study context.

Previous studies have found differing results on whether class size matters for implementation of active-learning strategies (Ebert-May et al., 2011; Stains et al., 2018). Our study contributes to this existing literature, as we found that smaller class sizes positively correlates with the implementation of active-learning strategies in our study context. Together, our results and the existing literature may suggest that class size alone is not sufficient to predict or support the implementation of active-learning strategies.

Classrooms are situated in larger contexts such as campuses, and our results suggest that campus can potentially influence the implementation of active-learning strategies. While all study campuses have professional development opportunities for instructors, Campus 3 has additional unique contexts with initiatives related to active learning described in the Methods section which may have resulted in more teaching pedagogy training compared to Campus 2. The initiatives at Campus 3 could potentially serve as a model for other campuses for improving their courses through increased implementation of active learning and evidence-based instructional practices.

### Limitations and future directions

We acknowledge that this work contains certain limitations. First, because of the labor-intensive nature of COPUS and our desire to observe a large number of courses, we could only sample a small proportion of the class sessions of each course. At the time of data collection, it was typical in the literature to only collect a week’s worth of observations (2–3 class sessions) to characterize instructional practice (e.g., in Stains et al., 2018). However, several studies since then have shown that to characterize the teaching styles of individual instructors, it is necessary to observe them as many as 9–11 times because instructors display a lot of variability session-to-session in how they teach (Sbeglia et al., 2021; Weston et al., 2021). Thus, we cannot make claims about the styles of individual instructors, only about the likelihood of general classes of instructors (TP/PoTs, etc.) to teach in certain ways. However, we recognize that more classroom observations could potentially demonstrate additional instructional variability and increase reliability (Goodridge et al., 2020; Stains et al., 2018). In future, we plan to complement COPUS with other classroom observation protocols that are easier to deploy for intensive sampling. For example, Decibel Analysis for Research in Teaching (DART) uses classroom recordings to determine the percentage of time spent with single voice (traditional lecture) or multiple or no voice (active learning) (Owens et al., 2017). While DART gives less detail about classroom activities, it is more automated so that we can more fully sample our courses.

Second, COPUS provides a limited lens for understanding instructional practices. While COPUS allows observers to quantify the time spent on various instructor and student behaviors occurring in the classroom, it does not examine the quality of these activities. COPUS also does not capture instructional practices that happen outside of the classroom, such as out-of-class assignments. A number of instruments have been developed over the years to document active learning in undergraduate STEM education, including reliable and validated self-report surveys, interviews, and classroom observation protocols (American Association for the Advancement of Science, 2013). The most direct approach to measure active learning is through classroom observations where trained observers document instructional practices in real time or via audio or video recordings (American Association for the Advancement of Science, 2013). There are several self-report instruments that are often used to measure active-learning strategies, including the Approaches to Teaching Inventory (ATI) (Trigwell & Prosser, 2004), the Teaching Practices Inventory (TPI) (Wieman & Gilbert, 2014), and the Postsecondary Instructional Practices Survey (PIPS) (Walter et al., 2016). However, there is a significant discrepancy between the degree to which faculty members report using active learning versus levels of active learning observable in video recordings of their classrooms (Ebert-May et al., 2011). Additionally, a multi-institutional study of introductory biology courses found that self-reports of active learning instruction were not associated with higher student learning gains (Andrews et al., 2011). Well-developed classroom observation protocols are often perceived as more objective than self-reported survey or interview data supplied by faculty members (American Association for the Advancement of Science, 2013). There are holistic observation protocols, like the Reformed Teaching Observation Protocol (RTOP) (Piburn et al., 2000), where the observer watches an entire class session and then rates each item with regard to the lesson as a whole. While holistic protocols, like RTOP, are widely used for detecting the degree to which classroom instruction uses student-centered, engaged learning practice, observers have to spend many hours to achieve high levels of inter-rater reliability (Piburn et al., 2000). The Classroom Discourse Observation Protocol (CDOP) could be used to evaluate the quality of instructional practices especially in relation to teacher discourse moves or the content-related conversations initiated by instructors (Kranzfelder et al., 2019). Also, content analysis of syllabus (Doolittle & Siudzinski, 2010) and survey instruments, such as the Teaching Practices Inventory (Wieman & Gilbert, 2014), could be used to examine instructional practices outside of the classroom.

Third, there are undoubtedly many instructor and demographic characteristics that we did not capture that are important for understanding the people and why particular individual instructors choose the teaching strategies they use. For demographic characteristics, we could only obtain gender of the instructors. Other instructor characteristics we would like to obtain for future research is, for example, pedagogical training (which may be a factor associated with active learning). Although only a small percentage of TP/PoTs have had formal training in education (nearly all have a PhD in their STEM discipline instead), the vast majority have participated in teaching-related professional development (Harlow et al., 2020). Such professional development may make them more likely to use active-learning pedagogical strategies. Similarly, we also have a limited understanding of instructor’s thoughts and beliefs about teaching and learning, which also are likely to influence their teaching practices. In our future work, we hope to capture a fuller picture of instructors and link their beliefs and training to their teaching practices.

Fourth, while understanding what instructor and classroom characteristics influence instructional practice is important, it is also important to link these practices to student outcomes (which were not collected for this study). While there is still much work to be done to associate particular active-learning strategies with specific student outcomes (Wieman, 2014), there have been no shortage of studies that associate active-learning strategies in general with better outcomes (Braxton et al., 2008; Freeman et al., 2014; Prince, 2004; Ruiz-Primo et al., 2011; Springer et al., 1999; Theobald et al., 2020). Our future work seeks to connect the instructor and classroom characteristics that influence instructional practices to student outcomes such as increased retention in STEM.

Finally, as with any study, our findings may not apply to other institutions, especially those that are substantially different from the ones analyzed here. Each university system, university, and department has its own history, politics, and culture around teaching, hiring, and evaluation. However, our study does include 18 departments across three universities, and many of the conclusions are consistent across those three universities. Although we cannot claim our findings are generalizable beyond the UC system, we demonstrate a possible outcome of having tenure-track education-focused faculty in hopes of inspiring more research about the impacts of this increasingly large group of instructors.

## Conclusion

Our study has broader implications for the use of education-focused academic positions as a structure for increasing the implementation of active-learning strategies in undergraduate STEM education. Even though our research focuses on TP/PoTs, there are other positions across different university systems that may have similar roles and thus potential impacts. For example, SFES (Bush et al., 2020), first described in the context of the California State University system, is a heterogeneous group of faculty in tenure-track and non-tenure track positions focusing on a variety of teaching-centered endeavors, including K-12 science education, DBER, the scholarship of teaching and learning, and undergraduate science education reform (Bush et al., 2006, 2011, 2013, 2015). Canadian universities employ permanent faculty called TFF who are involved in a combination of teaching, service, research, and other scholarly activities (Rawn & Fox, 2018).

While both SFES and TFF self-report knowledge of evidence-based instructional practices and/or engage in DBER (Bush et al., 2016; Bush et al., 2020; Rawn & Fox, 2018) our work is the first to identify through classroom observations that individuals within these education-focused academic positions who are more likely to implement active-learning strategies. These results serve as a baseline for further studies that can examine if TP/PoTs serve as change agents within their departments, not only by implementing active-learning strategies in their own classrooms but also by potentially influencing their departmental colleagues’ teaching through formal and informal interactions. In other existing studies, SFES self-report and consider departmental change as one of their important impacts (Bush et al., 2016, 2019). Therefore, adding similar studies on departmental change within the TP/PoTs context could further shed light on how education-focused academic positions more broadly may function in undergraduate STEM education.

This work highlights the use of a robust clustering methodology. As clusters can change with new data and new algorithms, using an ensemble improves the accuracy over a single classifier Moon et al. (2007). The methodology applied in this paper does not rely on a single set of COPUS codes, single clustering algorithm, single clustering ensemble, or single internal index. Instead we leverage the information from multiple COPUS datasets, carry out multiple clustering algorithms (with the cluster size varying), pool together cluster assignments using multiple ensembles, and use majority voting from each of the best ensembles to identify the final clusters that were used to address our research questions about the implementation of active learning by tenure-track teaching faculty.

### Supplementary Information

**Additional file 1:** Supplementary figures and tables.

## Data Availability

The datasets analyzed during the current study are not publicly available. Data are available upon reasonable request and with permission of local Institutional Review Board.

## Appendix A Abbreviations

COPUS Classroom Observation Protocol for Undergraduate STEM; CSPA Cluster-based Similarity Partitioning Algorithm; DART Decibel Analysis for Research in Teaching; DBER Discipline-Based Education Research; I &C Sciences Information and Computer Sciences; Instructor.Lec Instructor lecturing (presenting content, deriving mathematical results, presenting a problem solution, etc.); Instructor.RtW Instructor real-time writing on board, doc. projector, etc.; Instructor.DV Instructor showing or conducting a demo, experiment, simulation, video, or animation; Instructor.FUp Instructor follow-up/feedback on clicker question or activity to entire class; Instructor.PQ Instructor posing non-clicker question to students (non-rhetorical); Instructor.CQ Instructor asking a clicker question; Instructor.TAnQ Instructor listening to and answering student questions with entire class listening; Instructor.MG Instructor moving through class guiding ongoing student work during active learning task; Instructor.1o1 Instructor one-on-one extended discussion with one or a few individuals, not paying attention to the rest of the class; Instructor.Adm Instructor administration (assign homework, return tests, etc.); Instructor.W Instructor waiting when there is an opportunity for an instructor to be interacting with or observing/listening to student or group activities and the instructor is not doing so; Instructor.O Instructor other activities; I.Presenting Instructor presenting collapsed code (Instructor.Lec, Instructor.RtW, and Instructor.DV combined, same as I.Minimal); I.Guiding Instructor guiding collapsed code (Instructor.FUp, Instructor.PQ, Instructor.CQ, Instructor.AnQ, Instructor.MG, and Instructor.1o1 combined); I.Administration Instructor administration collapsed code (same as Instructor.Adm); I.Other Instructor other collapsed code (Instructor.W and Instructor.Other combined); I.Interactive Instructor interactive novel code (Instructor.MG and Instructor.1o1 combined); I.Thinking Instructor promoting individual thinking novel code (Instructor.PQ and Instructor.CQ combined); I.Few Instructor attending to one or few students novel code (Instructor.FUp and Instructor.AnQ combined); I.Minimal Instructor minimal interactions novel code (Instructor.Lec, Instructor.RtW and Instructor.DV combined, same as I.Presenting); I.Miscellaneous Instructor miscellaneous novel); LCE Linkage Clustering Ensemble; PAM Partitioning Around Medoids; RQ Research Questions; SATAL Students Assessing Teaching and Learning; SFES Science Faculty with Education Specialties; STEM science, Technology, Engineering, and Mathematics; Student.L Student listening to instructor/taking notes, etc.; Student.AnQ Student answering a question posed by the instructor with rest of class listening; Student.SQ Student asks question; Student.WC Students engaged in whole class discussion by offering explanations, opinion, judgment, etc., to whole class, often facilitated by instructor; Student.SP Student(s) presentation; Student.Ind Student individual thinking/problem solving when an instructor explicitly asks students to think about a clicker question or another question/problem on their own; Student.CG Students discuss clicker question in groups; Student.WG Students working in groups on worksheet activity; Student.OG Student assigned group activity, such as responding to instructor question; Student.Prd Student(s) make a prediction about the outcome of demo or experiment; Student.TQ Student take a test or quiz; Student.W Students waiting (instructor late, working on fixing AV problems, instructor otherwise occupied, etc.); Student.O Student other activities; S.Receiving Students receiving collapsed code (same as Student.L and S.Minimal); S.Working Student working collapsed code (Student.Ind, Student.CG, Student.WG, Student.OG, and Student.Prd combined); S.Talking Student talking collapsed code (Student.AnQ, Student.SQ, Student.WC, Student.SP combined); S.Other Student other collapsed code (Student.W and Student.Other combined, same as student other novel code); S.Interactive Student interactive novel code (Student.CG, Student.WG, and Student.OG combined); S.Thinking Student thinking novel code (Student.Ind, Student.Prd, and Student.CQ); S.Few Student one or a few students interacting with the instructor or class novel code (Student.AnQ, Student.SQ, and Student.SP combined); S.Minimal Student minimal interaction novel code (same as Student.L and S.Receiving); S.Other Student other collapsed and novel code (Student.W and Student.Other combined); TP/PoT Teaching Professor or Professor of Teaching; TFF Teaching Focused Faculty; UC University of California
