# Supplementary material for: Predicting implementation of active learning by tenure-track teaching faculty using robust cluster analysis
Source: Int J STEM Educ. 2022 Jul 28;9(1):49. doi: 10.1186/s40594-022-00365-9 (PMC9334417; doi:10.1186/s40594-022-00365-9)
Supplement: Supplementary file 1 — Additional file 1: Supplementary figures and tables. [file 40594_2022_365_MOESM1_ESM.pdf]

# Supplemental Materials

## Predicting Implementation of Active Learning by Tenure-Track Teaching Faculty Using Robust Cluster Analysis

Kameryn Denaro[1*], Petra Kranzfelder[2], Melinda T. Owens[3,4], Brian Sato[1,5], Austin L. Zuckerman[3,6†], Rebecca A. Hardesty[7†], Adriana Signorini[8†], Andrea Aebersold[1†], Mayank Verma[2†] and Stanley M. Lo[2,3*]

[1*]Division of Teaching Excellence and Innovation, University of California Irvine, 653 E Peltason Drive, Irvine, 92697, CA, US.
[2]Molecular & Cellular Biology, University of California Merced, 5200 North Lake Road, Merced, CA, 95343, CA, US.
[3]Section of Neurobiology, Division of Biological Sciences, University of California San Diego, 9500 Gilman Drive, La Jolla, 92093, CA, US.
[4]Program in Mathematics and Science Education, University of California San Diego, 9500 Gilman Drive, La Jolla, 92093, CA, US.
[5*]Molecular Biology and Biochemistry, University of California Irvine, 2238 McGaugh Hall, Irvine, 92697, CA, US.
[6]Section of Cell and Developmental Biology, Division of Biological Sciences, University of California San Diego, 9500 Gilman Drive, La Jolla, 92093, CA, US.
[7]Division of Biological Sciences, University of California San Diego, 9500 Gilman Drive, La Jolla, 92093, CA, US.
[8]Students Assessing Teaching and Learning (SATAL) Program, Center for Engaged Teaching and Learning, University of California Merced, 5200 North Lake Road, Merced, CA, 95343, CA, US.

*Corresponding author(s). E-mail(s): kdenaro@uci.edu; smlo@ucsd.edu;

Contributing authors: pkranzfelder@ucmerced.edu; mtowens@ucsd.edu; bsato@uci.edu; azuckerm@ucsd.edu; rebeccaahardesty@gmail.com; asignorini@ucmerced.edu; andrea.aebersold@uci.edu; mayankv@uci.edu;
†These authors contributed equally to this work.

# 1 COPUS Codes by Faculty Type

Summary statistics of the raw percentage of time spent on each code split by faculty type can be found in the main paper. The corresponding standardized percentage of time spent on each code can be found in Table S2. We see significant differences (adjusted for multiple testing) across faculty types (denoted with an asterisk) for many of the COPUS codes.

# 2 Cluster Analysis Details

For the five COPUS datasets (original codes, collapsed codes, analyzer codes, novel codes, and unique codes) and 11 clustering algorithms ($k$-means, partitioning around medoids, non-negative matrix factorization using euclidean distance, hierarchical clustering, divisive analysis clustering, affinity propagation, spectral clustering using radial-basis kernel function, Gaussian mixture model, self-organizing map with hierarchical clustering, fuzzy $C$-means clustering, and hierarchical density-based spatial clustering of applications with noise), the optimal number of clusters was two with only one exception (the original codes). The boxplots for each of the codes split by final cluster assignment are in Figures S1-S38. Table S1 gives the internal indices that were used to determine the best number of clusters ($k = 2, \ldots, 9$) for the 11 different clustering algorithms and the best choice of ensemble for each dataset. Figure S39-S43 displays the relative change in the area under the cumulative distribution function curve for cluster sizes $k = 2, \ldots, 9$ across the "original", "analyzer", "collapsed", "novel", and "unique" COPUS codes. Figure S44-S48 gives a heatmap of the values of the internal indices for the ranked clustering algorithms across the "original", "analyzer", "collapsed", "novel", and "unique" COPUS codes. Figure S49-S53 tracks the cluster assignments across $k$ ($k = 2, \ldots, 9$) for the different clustering algorithms across the "original", "analyzer", "collapsed", "novel", and "unique" COPUS codes. Table S3-S17 gives the values of the respective internal indices with cluster size $k = 2$ across the "original", "analyzer", "collapsed", "novel", and "unique" COPUS codes. The top choice of cluster ensemble for each of the five COPUS datasets is presented in Table S18 for combining the results of the individual clustering algorithms with $k = 2$. The cluster ensemble chosen for the original COPUS codes and all COPUS codes was the CSPA algorithm. For the collapsed COPUS codes, the best choice of ensemble clustering algorithm was

k-modes. The best ensemble was majority voting for the novel codes. For the analyzer COPUS codes, all of the ensembles yielded similar results. However, none of the ensemble algorithms performed well for the analyzer codes. In this case, we used the best individual clustering algorithm (this step is marked with an asterisks in Figure 1 of the paper). Table S19 gives the values of the internal indices for the best choice of the ensemble algorithm for each COPUS dataset. The instructor and classroom characteristics for the individual clustering ensembles of the five datasets (original, analyzer, collapsed, novel, and unique codes) can be found in Table S20-S26). The logistic regression models with the instructor and classroom characteristics and the odds of being in the active-learning cluster based on the cluster ensembles of the 5 different datasets (original, analyzer, collapsed, novel, and unique codes) in the Tables S27-S31).

# 3 Supplemental Figures

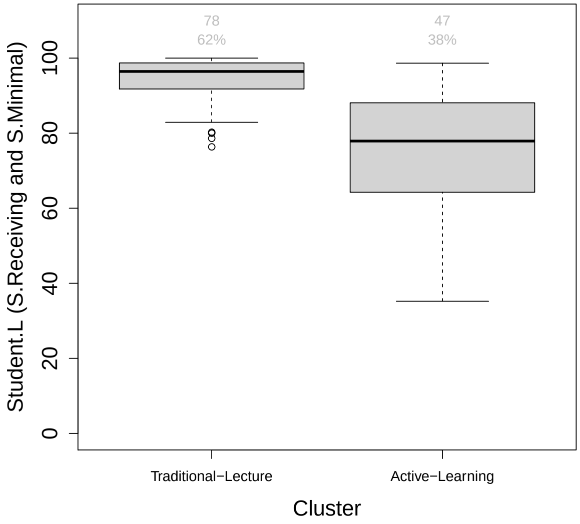


**Fig. S1** Boxplot of student Student.L (listening to instructor/taking notes etc.) split by final cluster assignment. Student.L is the same as S.Receiving and S.Minimal.

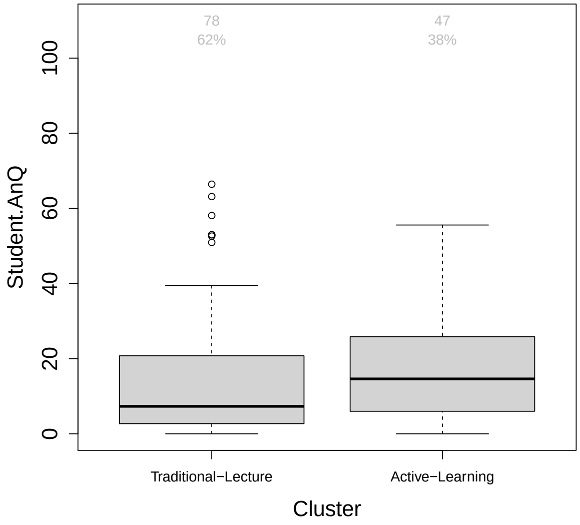


**Fig. S2** Boxplot of Student.AnQ (student answering a question posed by the instructor with rest of class listening) split by final cluster assignment.

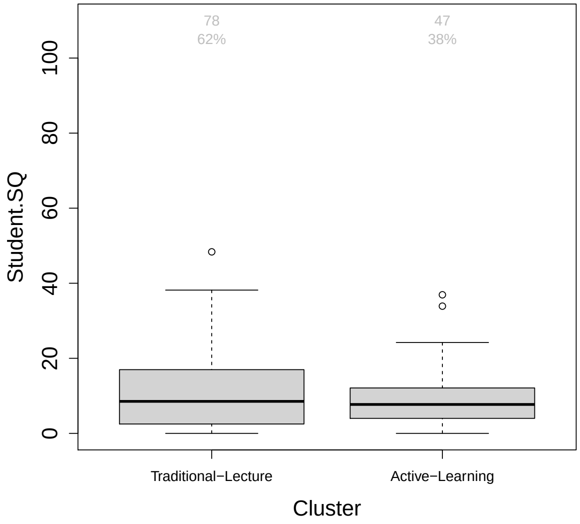


**Fig. S3** Boxplot of Student.SQ (student asks question) split by final cluster assignment.

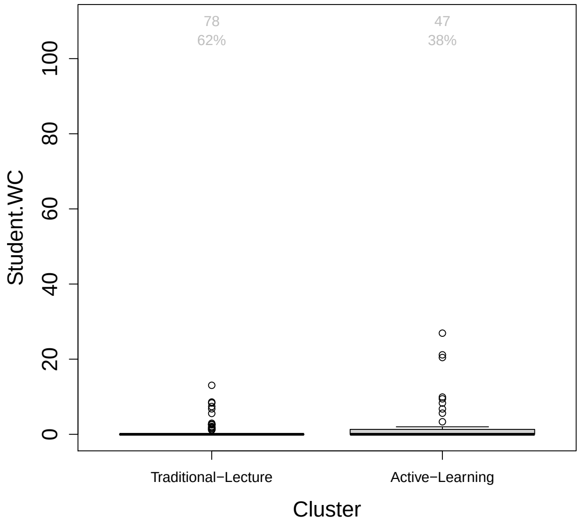


**Fig. S4** Boxplot of Student.WC (engaged in whole class discussion by offering explanations, opinion, judgment, etc. to whole class often facilitated by instructor) split by final cluster assignment.

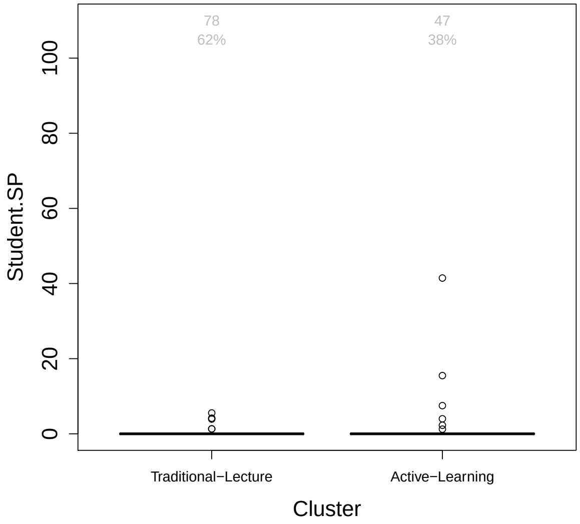


**Fig. S5** Boxplot of Student.SP (presentation by student[s]) split by final cluster assignment.

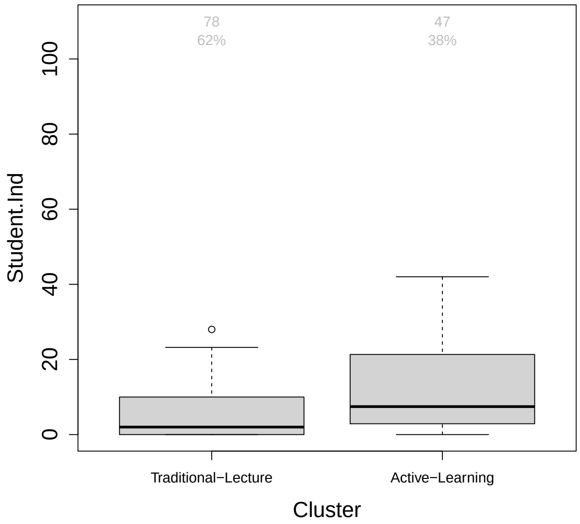


**Fig. S6** Boxplot of Student.Ind (individual thinking/problem solving) split by final cluster assignment.

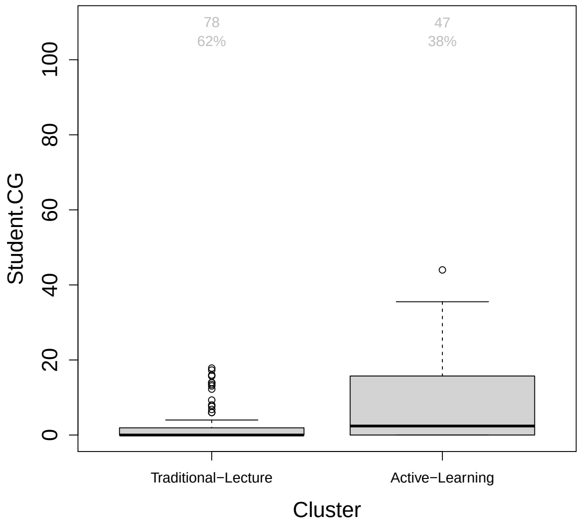


**Fig. S7** Boxplot of Student.CG (discuss clicker question in groups of 2 or more students) split by final cluster assignment.

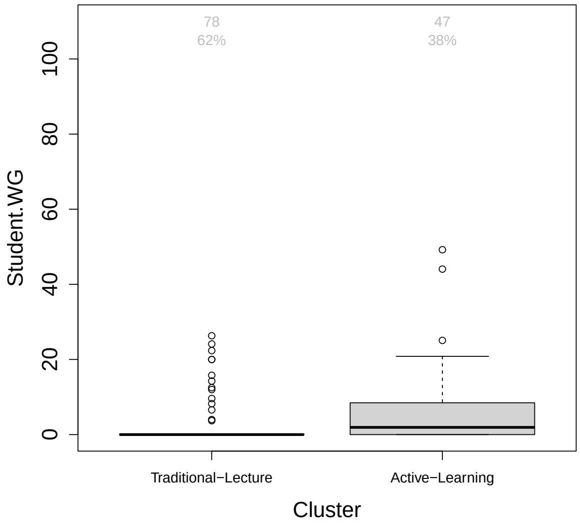


**Fig. S8** Boxplot of Student.WG (working in groups on worksheet activity) split by final cluster assignment.

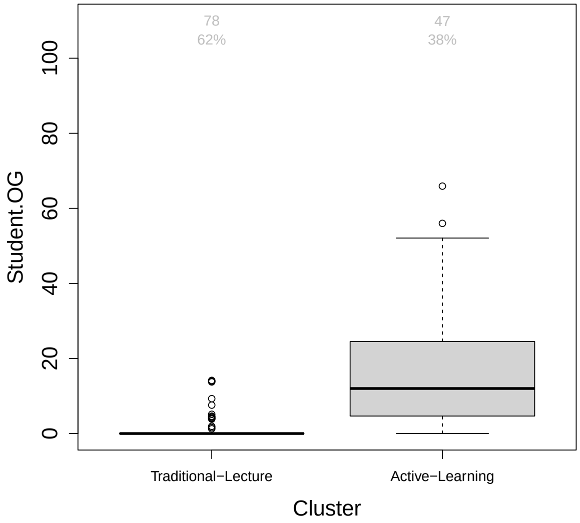


**Fig. S9** Boxplot of Student.OG (other assigned group activity) split by final cluster assignment.

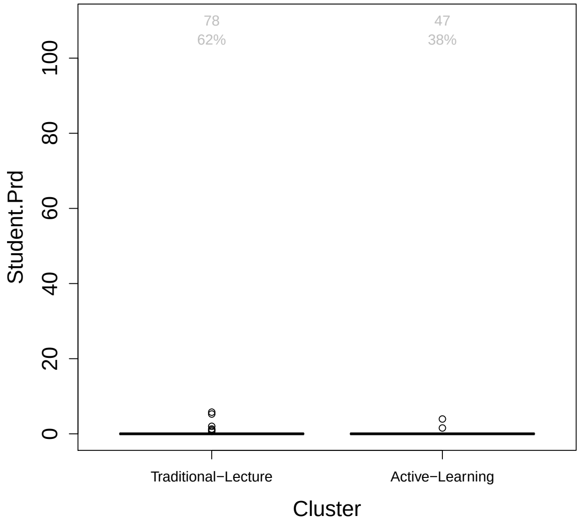


**Fig. S10** Boxplot of Student.Prd (making a prediction about the outcome of demo or experiment) split by final cluster assignment.

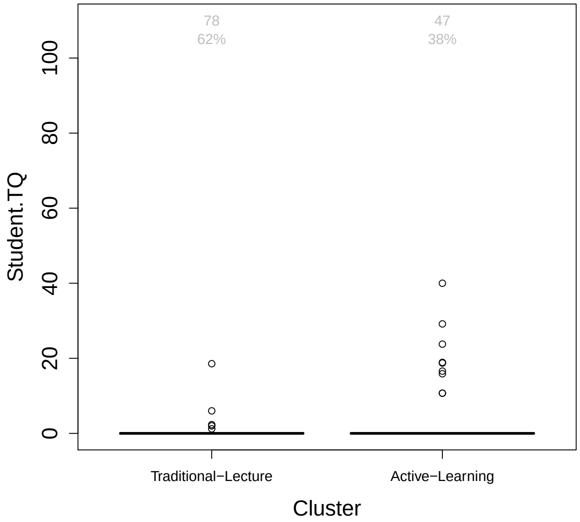


**Fig. S11** Boxplot of Student.TQ (test or quiz) split by final cluster assignment.

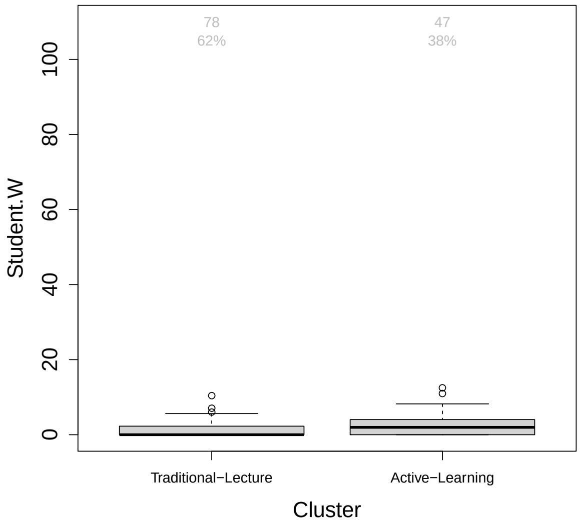


**Fig. S12** Boxplot of Student.W (waiting) split by final cluster assignment.

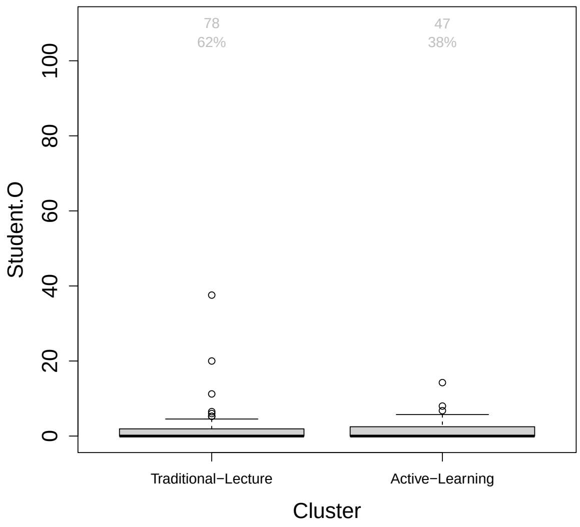


**Fig. S13** Boxplot of Student.Other (student other) split by final cluster assignment.

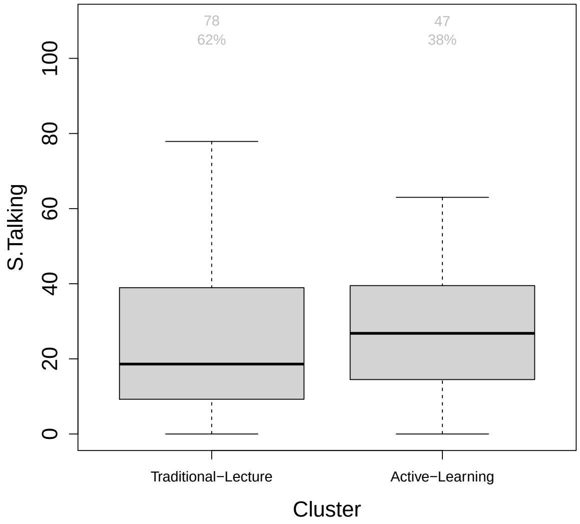


**Fig. S14** Boxplot of S.Talking (Student.AnQ, Student.SQ, Student.WC, Student.SP combined) split by final cluster assignment.

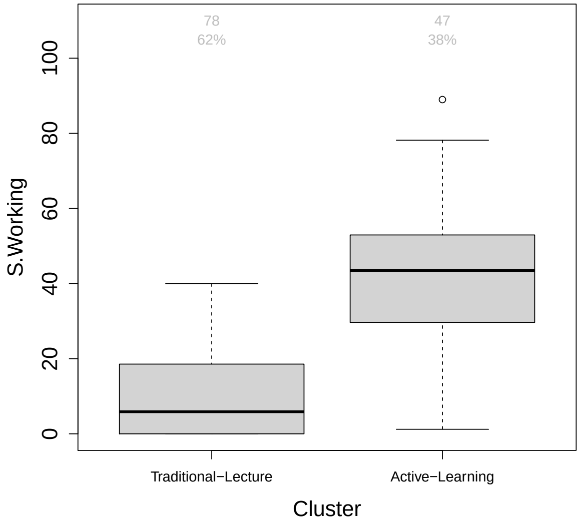


**Fig. S15** Boxplot of S.Working (Student.Ind, Student.CG, Student.WG, Student.OG, Student.Prd, Student.TQ combined) split by final cluster assignment.

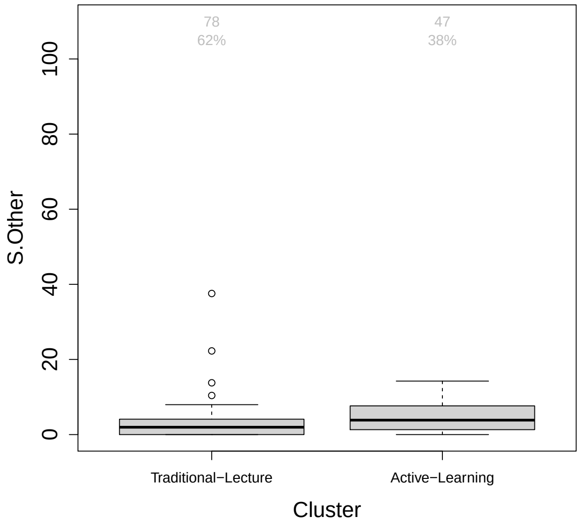


**Fig. S16** Boxplot of S.Other (Student.W and Student.Other combined) split by final cluster assignment.

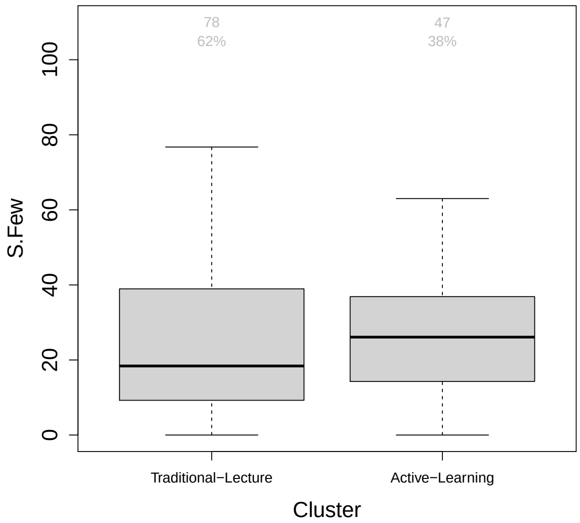


**Fig. S17** Boxplot of S.Few (Student.AnQ, Student.SQ, Student.SP combined) split by final cluster assignment.

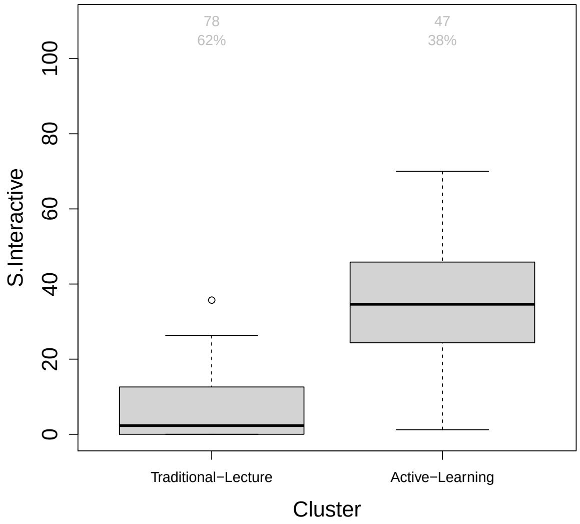


**Fig. S18** Boxplot of S.Interactive (Student.WC, Student.CG, Student.WG, Student.OG combined) split by final cluster assignment.

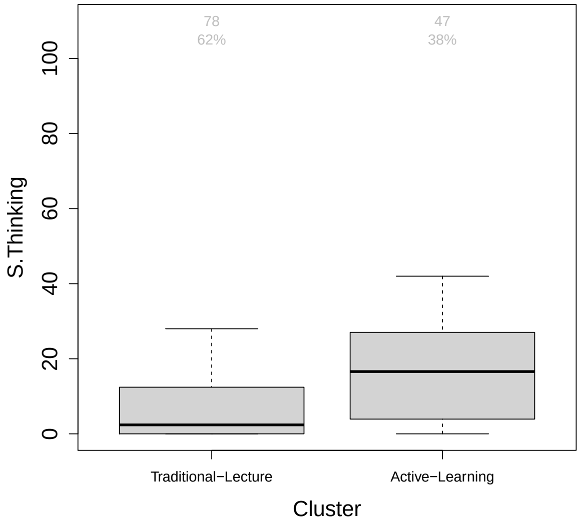


**Fig. S19** Boxplot of S.Thinking (Student.Ind, Student.Prd, Student.TQ combined) split by final cluster assignment.

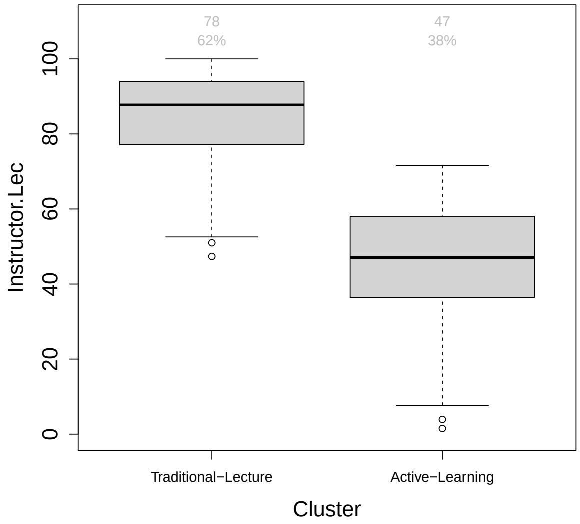


**Fig. S20** Boxplot of Instructor.Lec (lecturing, presenting content, deriving mathematical results, presenting a problem solution, etc.) split by final cluster assignment.

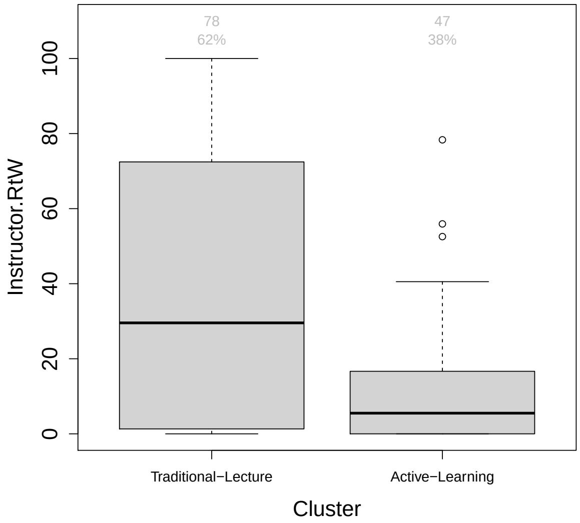


**Fig. S21** Boxplot of Instructor.RtW (real-time writing on board, doc. projector, etc.) split by final cluster assignment.

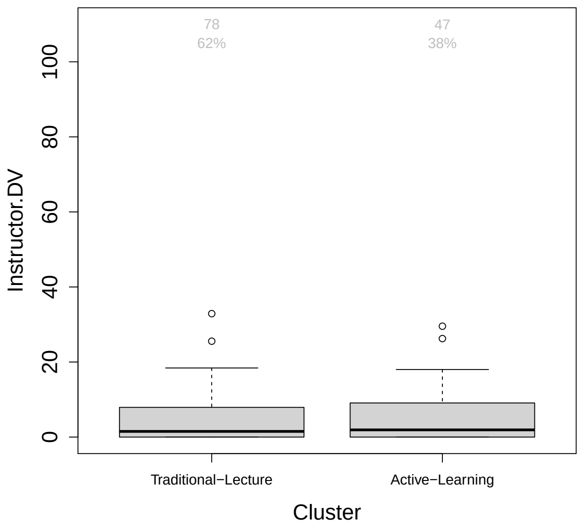


**Fig. S22** Boxplot of Instructor.DV (showing or conducting a demo, experiment, simulation, video, or animation) split by final cluster assignment.

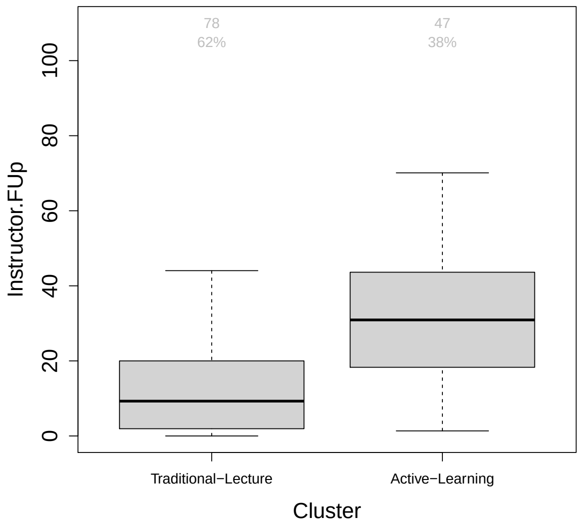


**Fig. S23** Boxplot of Instructor.FUp (follow-up/feedback on clicker question or activity to entire class) split by final cluster assignment.

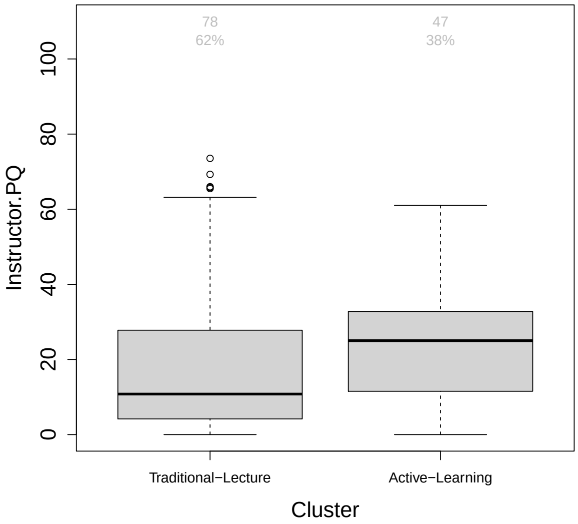


**Fig. S24** Boxplot of Instructor.PQ (posing non-clicker question to students) split by final cluster assignment.

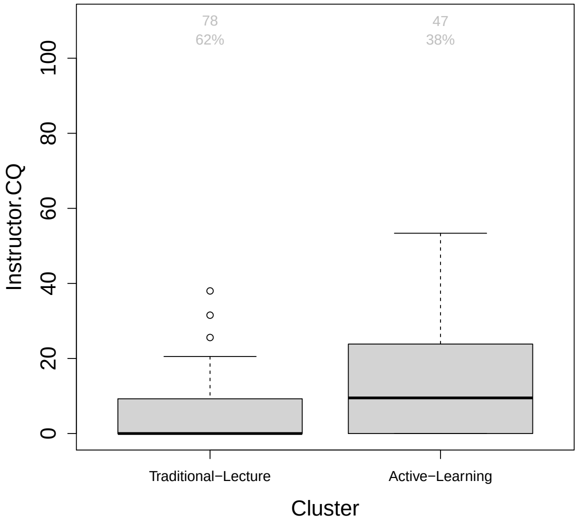


**Fig. S25** Boxplot of Instructor.CQ (asking a clicker question) split by final cluster assignment.

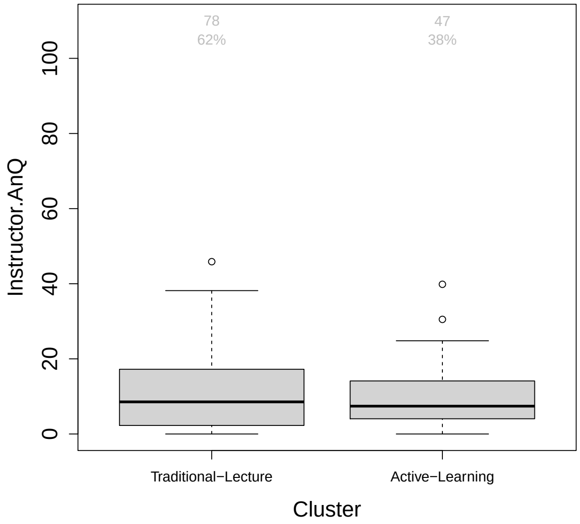


**Fig. S26** Boxplot of Instructor.AnQ (listening to and answering student questions with entire class listening) split by final cluster assignment.

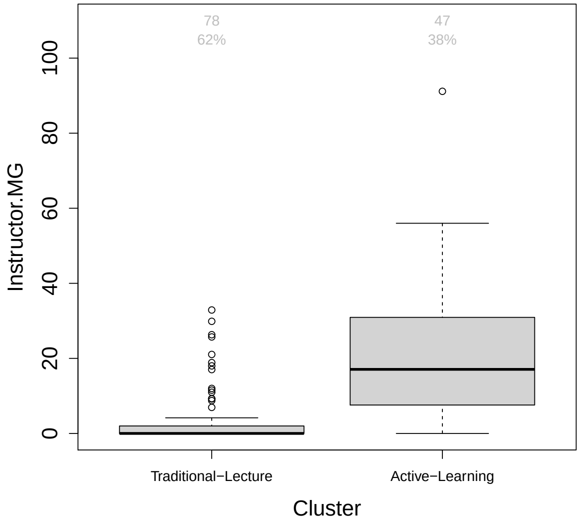


**Fig. S27** Boxplot of Instructor.MG (moving through class guiding ongoing student work during active learning task) split by final cluster assignment.

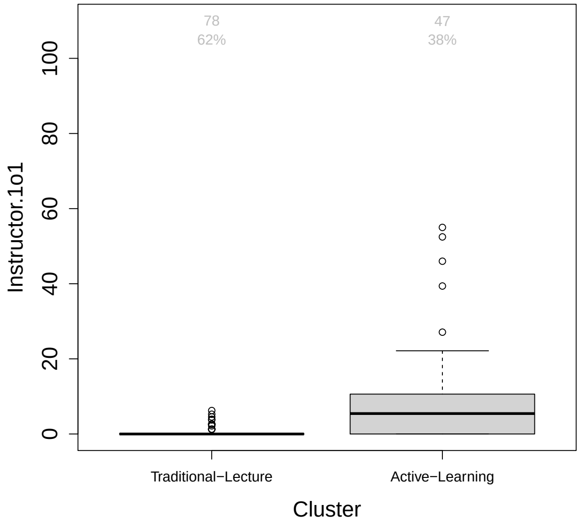


**Fig. S28** Boxplot of Instructor.1o1 (one-on-one extended discussion with on or a few individuals, not paying attention to teh rest of the class) split by final cluster assignment.

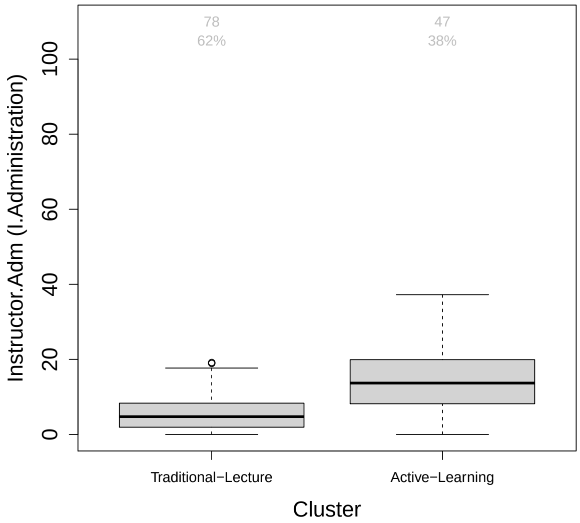


**Fig. S29** Boxplot of Instructor.Adm (administration such as assigning homework, returning tests, etc.) split by final cluster assignment. Same as I.Administration.

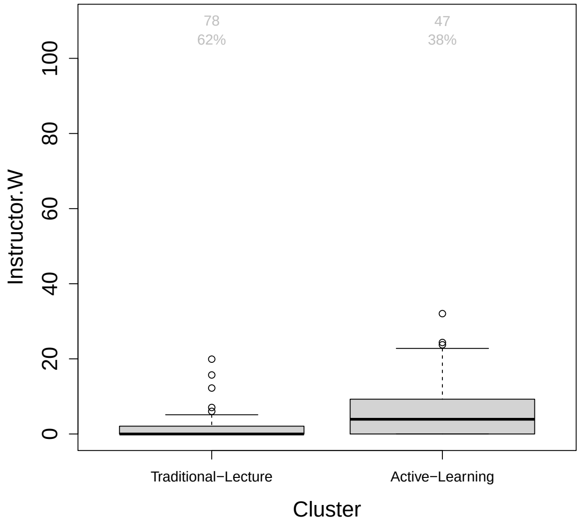


**Fig. S30** Boxplot of Instructor.W (waiting when there is an opportunity for an instructor to be interacting with or observing/listening to student or group activities and the instructor is not doing so) split by final cluster assignment.

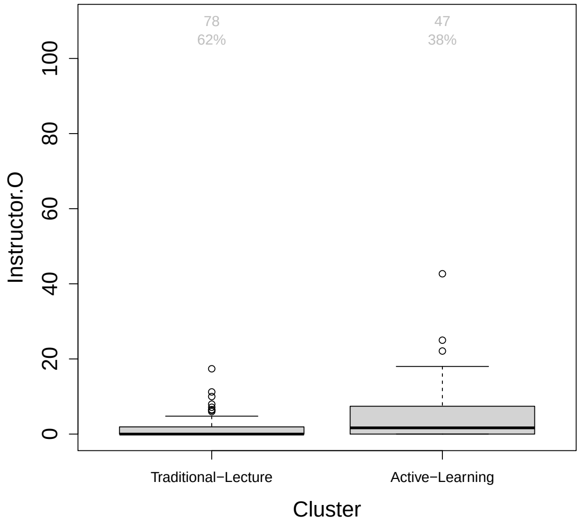


**Fig. S31** Boxplot of Instructor.O (instructor other) split by final cluster assignment.

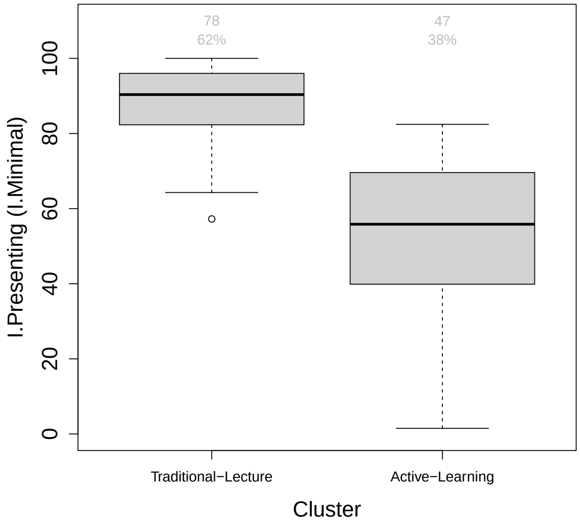


**Fig. S32** Boxplot of I.Presenting (Instructor.Lec, Instructor.RtW, Instructor.DV, combined) split by final cluster assignment. Same as I.Minimal.

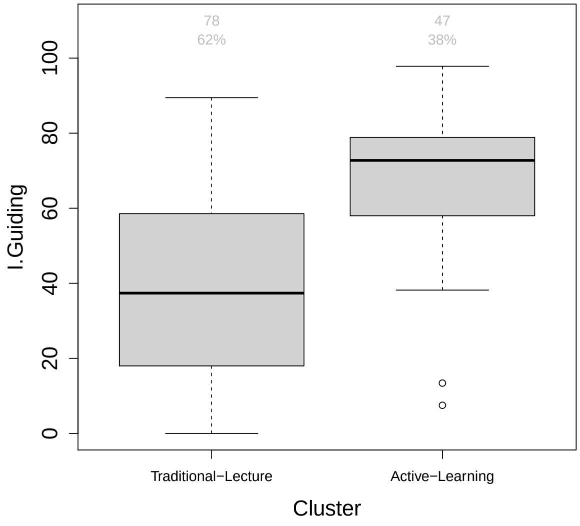


**Fig. S33** Boxplot of I.Guiding (Instructor.FUp, Instructor.PQ, Instructor.CQ, Instructor.AnQ, Instructor.MG, Instructor.1o1 combined) split by final cluster assignment.

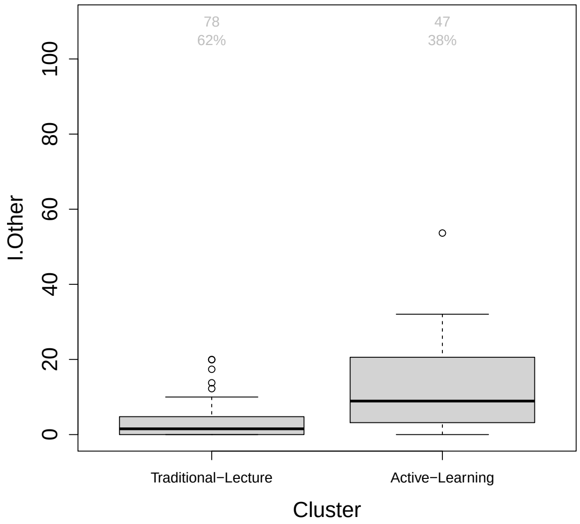


**Fig. S34** Boxplot of I.Other (Instructor.W, Instructor.Other combined) split by final cluster assignment.

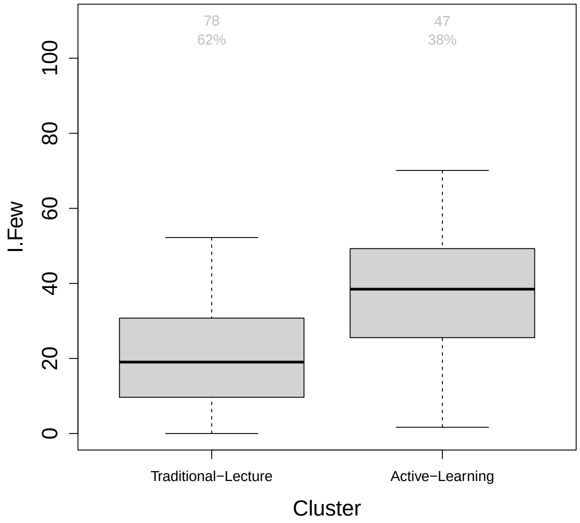


**Fig. S35** Boxplot of I.Few (Instructor.FUp, Instructor.AnQ combined) split by final cluster assignment.

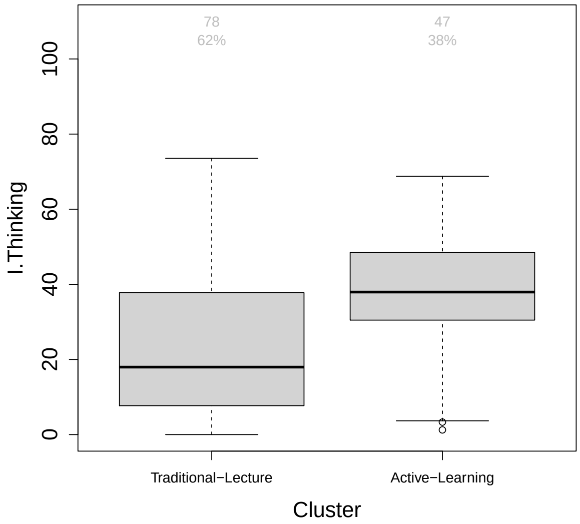


**Fig. S36** Boxplot of I.Thinking (Instructor.PQ, Instructor.CQ combined) split by final cluster assignment.

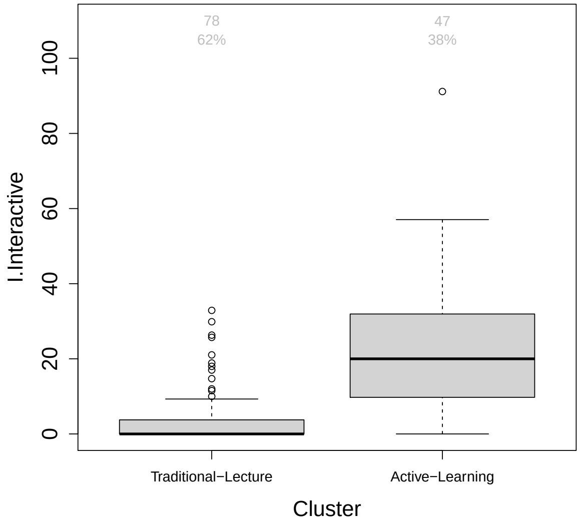


**Fig. S37** Boxplot of I.Interactive (Instructor.MG, Instructor.1o1 combined) split by final cluster assignment.

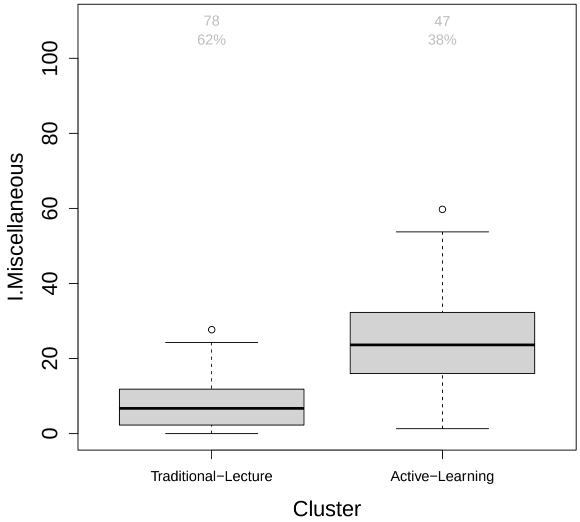


**Fig. S38** Boxplot of I.Miscellaneous (Instructor.Adm, Instructor.W, Instructor.Other combined) split by final cluster assignment.

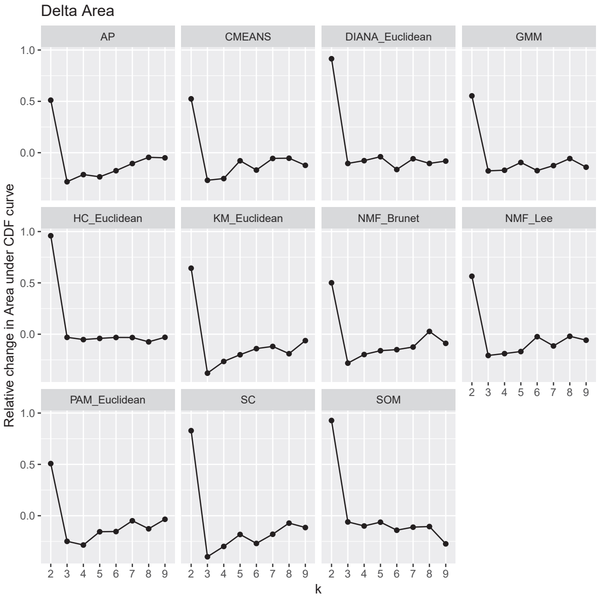


**Fig. S39** Relative change in the area under the cumulative distribution function (CDF) curve for cluster sizes of $k = 2, \ldots, 9$ for different clustering algorithms (affinity propagation [AP], fuzzy $C$-means clustering [CMEANS], divisive analysis clustering [DIANA], Gaussian mixture model [GMM], hierarchical clustering [HC Euclidean], $k$-means [KM Euclidean], non-negative matrix factorization [NMF Brunet and NMF Lee], partitioning around medoids [PAM Euclidean], spectral clustering using radial-basis kernel function [SC], and self-organizing map with hierarchical clustering [SOM]) of the "original" COPUS codes.

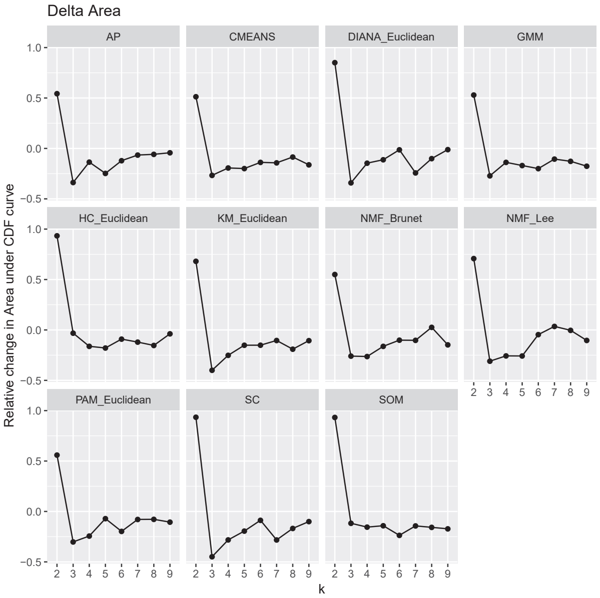


**Fig. S40** Relative change in the area under the cumulative distribution function (CDF) curve for cluster sizes of $k = 2, \ldots, 9$ for different clustering algorithms (affinity propagation [AP], fuzzy $C$-means clustering [CMEANS], divisive analysis clustering [DIANA], Gaussian mixture model [GMM], hierarchical clustering [HC Euclidean], $k$-means [KM Euclidean], non-negative matrix factorization [NMF Brunet and NMF Lee], partitioning around medoids [PAM Euclidean], spectral clustering using radial-basis kernel function [SC], and self-organizing map with hierarchical clustering [SOM]) of the "analyzer" COPUS codes.

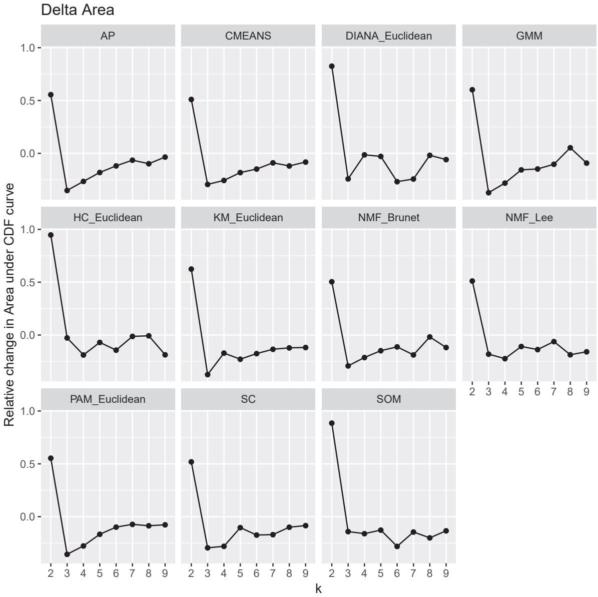


**Fig. S41** Relative change in the area under the cumulative distribution function (CDF) curve for cluster sizes of $k = 2, \ldots, 9$ for different clustering algorithms (affinity propagation [AP], fuzzy $C$-means clustering [CMEANS], divisive analysis clustering [DIANA], Gaussian mixture model [GMM], hierarchical clustering [HC Euclidean], $k$-means [KM Euclidean], non-negative matrix factorization [NMF Brunet and NMF Lee], partitioning around medoids [PAM Euclidean], spectral clustering using radial-basis kernel function [SC], and self-organizing map with hierarchical clustering [SOM]) of the "collapsed" COPUS codes.

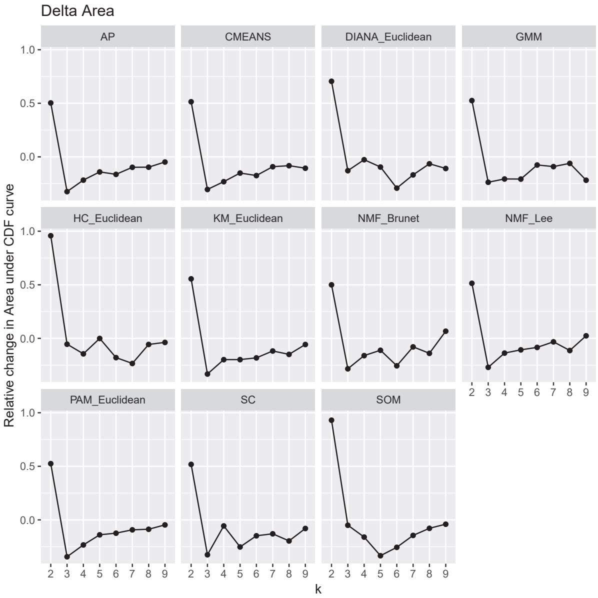


**Fig. S42** Relative change in the area under the cumulative distribution function (CDF) curve for cluster sizes of $k = 2, \ldots, 9$ for different clustering algorithms (affinity propagation [AP], fuzzy $C$-means clustering [CMEANS], divisive analysis clustering [DIANA], Gaussian mixture model [GMM], hierarchical clustering [HC Euclidean], $k$-means [KM Euclidean], non-negative matrix factorization [NMF Brunet and NMF Lee], partitioning around medoids [PAM Euclidean], spectral clustering using radial-basis kernel function [SC], and self-organizing map with hierarchical clustering [SOM]) of the "novel" COPUS codes.

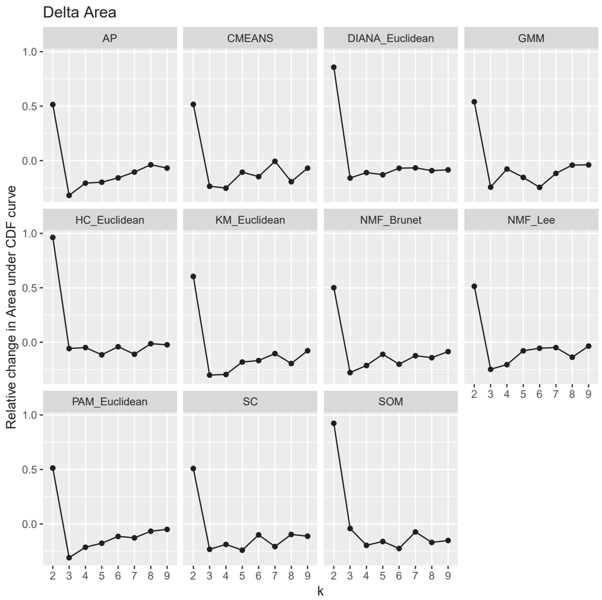


**Fig. S43** Relative change in the area under the cumulative distribution function (CDF) curve for cluster sizes of $k = 2, \ldots, 9$ for different clustering algorithms (affinity propagation [AP], fuzzy $C$-means clustering [CMEANS], divisive analysis clustering [DIANA], Gaussian mixture model [GMM], hierarchical clustering [HC Euclidean], $k$-means [KM Euclidean], non-negative matrix factorization [NMF Brunet and NMF Lee], partitioning around medoids [PAM Euclidean], spectral clustering using radial-basis kernel function [SC], and self-organizing map with hierarchical clustering [SOM]) of the "unique" COPUS codes.

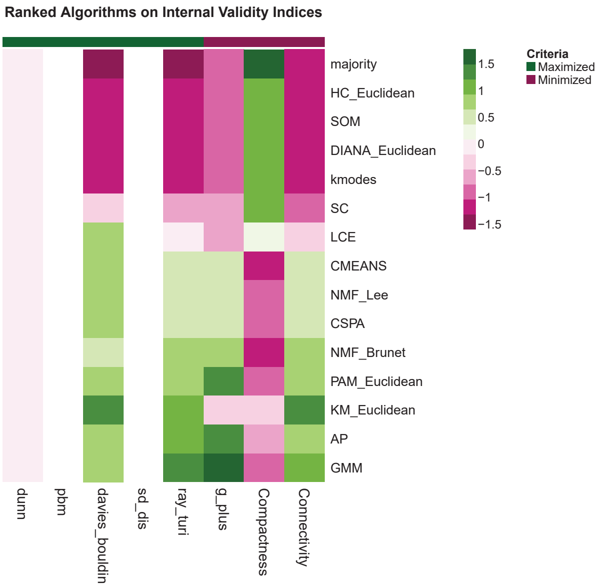


**Fig. S44** Ranked algorithms on internal validity indices for different clustering algorithms (affinity propagation [AP], fuzzy $C$-means clustering [CMEANS], divisive analysis clustering [DIANA], Gaussian mixture model [GMM], hierarchical clustering [HC Euclidean], $k$-means [KM Euclidean], non-negative matrix factorization [NMF Brunet and NMF Lee], partitioning around medoids [PAM Euclidean], spectral clustering using radial-basis kernel function [SC], and self-organizing map with hierarchical clustering [SOM]) and different cluster ensembles (cluster-based similarity partitioning algorithm [$CSPA$], $k$-modes [kmodes], majority voting [majority], linkage clustering ensemble [LCE]) of the "original" COPUS codes.

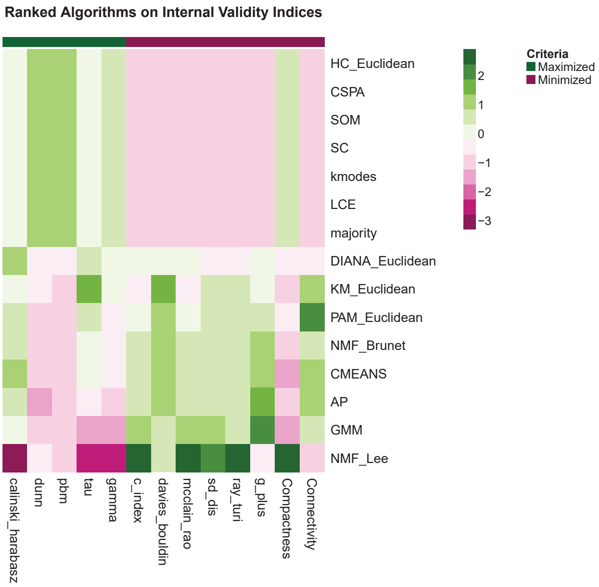


**Fig. S45** Ranked algorithms on internal validity indices for different clustering algorithms (affinity propagation [AP], fuzzy $C$-means clustering [CMEANS], divisive analysis clustering [DIANA], Gaussian mixture model [GMM], hierarchical clustering [HC Euclidean], $k$-means [KM Euclidean], non-negative matrix factorization [NMF Brunet and NMF Lee], partitioning around medoids [PAM Euclidean], spectral clustering using radial-basis kernel function [SC], and self-organizing map with hierarchical clustering [SOM]) and different cluster ensembles (cluster-based similarity partitioning algorithm [$CSPA$], $k$-modes [kmodes], majority voting [majority], linkage clustering ensemble [LCE]) of the "analyzer" COPUS codes.

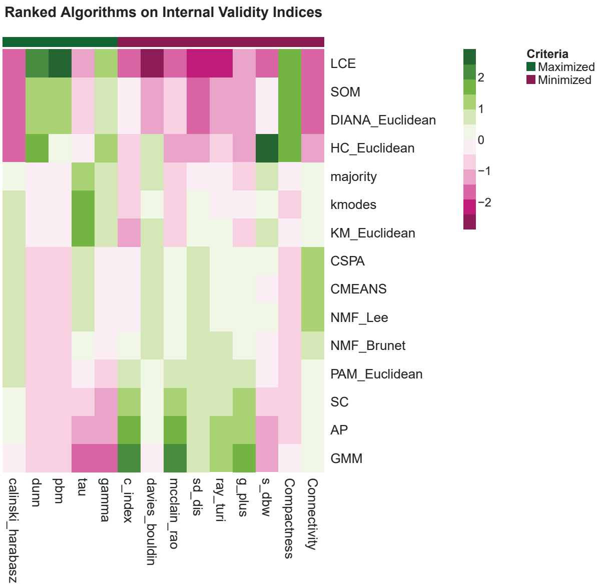


**Fig. S46** Ranked algorithms on internal validity indices for different clustering algorithms (affinity propagation [AP], fuzzy $C$-means clustering [CMEANS], divisive analysis clustering [DIANA], Gaussian mixture model [GMM], hierarchical clustering [HC Euclidean], $k$-means [KM Euclidean], non-negative matrix factorization [NMF Brunet and NMF Lee], partitioning around medoids [PAM Euclidean], spectral clustering using radial-basis kernel function [SC], and self-organizing map with hierarchical clustering [SOM]) and different cluster ensembles (cluster-based similarity partitioning algorithm [$CSPA$], $k$-modes [kmodes], majority voting [majority], linkage clustering ensemble [LCE]) of the "collapsed" COPUS codes.

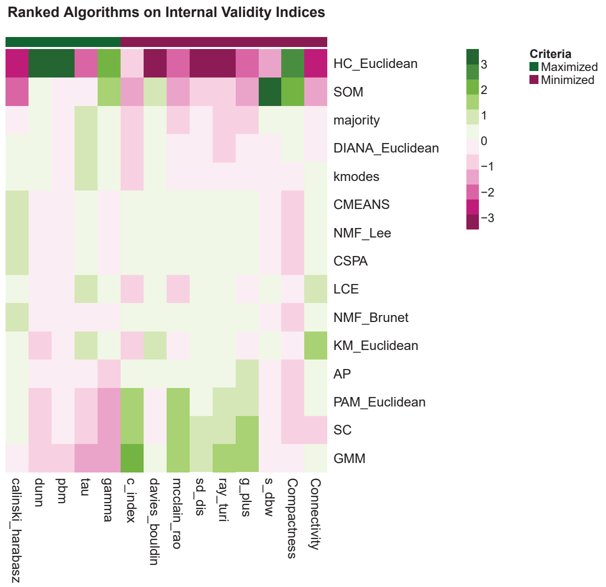


**Fig. S47** Ranked algorithms on internal validity indices for different clustering algorithms (affinity propagation [AP], fuzzy $C$-means clustering [CMEANS], divisive analysis clustering [DIANA], Gaussian mixture model [GMM], hierarchical clustering [HC Euclidean], $k$-means [KM Euclidean], non-negative matrix factorization [NMF Brunet and NMF Lee], partitioning around medoids [PAM Euclidean], spectral clustering using radial-basis kernel function [SC], and self-organizing map with hierarchical clustering [SOM]) and different cluster ensembles (cluster-based similarity partitioning algorithm [*CSPA*], $k$-modes [kmodes], majority voting [majority], linkage clustering ensemble [LCE]) of the "novel" COPUS codes.

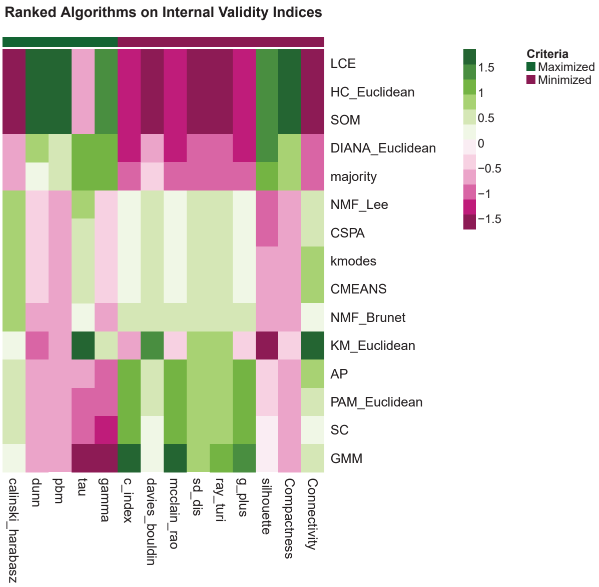


**Fig. S48** Ranked algorithms on internal validity indices for different clustering algorithms (affinity propagation [AP], fuzzy $C$-means clustering [CMEANS], divisive analysis clustering [DIANA], Gaussian mixture model [GMM], hierarchical clustering [HC Euclidean], $k$-means [KM Euclidean], non-negative matrix factorization [NMF Brunet and NMF Lee], partitioning around medoids [PAM Euclidean], spectral clustering using radial-basis kernel function [SC], and self-organizing map with hierarchical clustering [SOM]) and different cluster ensembles (cluster-based similarity partitioning algorithm [$CSPA$], $k$-modes [kmodes], majority voting [majority], linkage clustering ensemble [LCE]) of the “unique” COPUS codes.

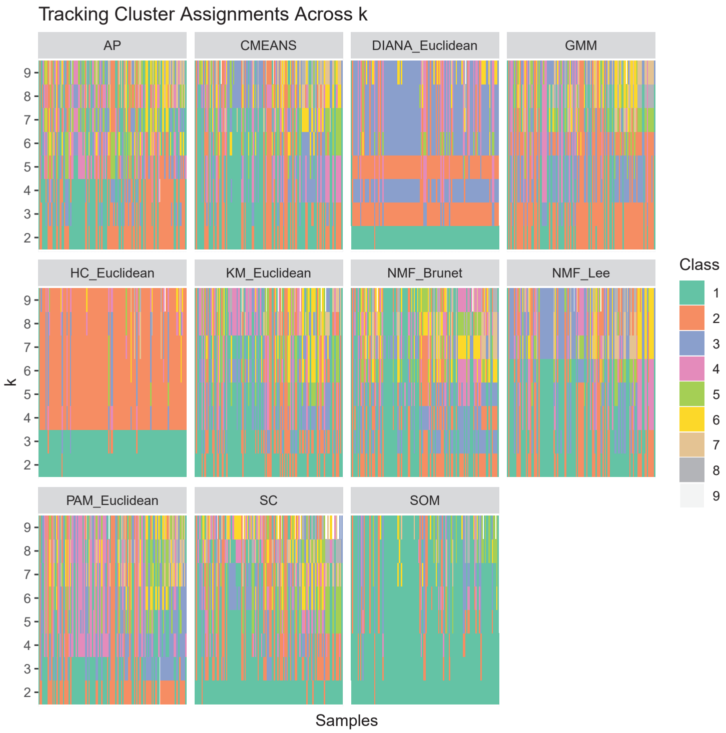


**Fig. S49** Tracking cluster assignments across $k$ ($k = 2, \ldots, 9$) for different clustering algorithms (affinity propagation [AP], fuzzy $C$-means clustering [CMEANS], divisive analysis clustering [DIANA], Gaussian mixture model [GMM], hierarchical clustering [HC Euclidean], $k$-means [KM Euclidean], non-negative matrix factorization [NMF Brunet and NMF Lee], partitioning around medoids [PAM Euclidean], spectral clustering using radial-basis kernel function [SC], and self-organizing map with hierarchical clustering [SOM]) of the "original" COPUS codes.

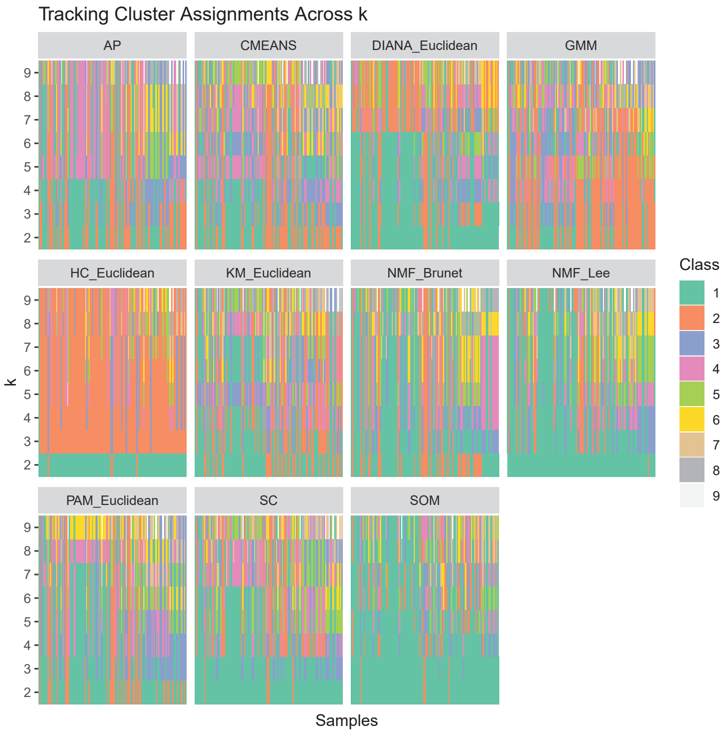


**Fig. S50** Tracking cluster assignments across $k$ ($k = 2, \ldots, 9$) for different clustering algorithms (affinity propagation [AP], fuzzy $C$-means clustering [CMEANS], divisive analysis clustering [DIANA], Gaussian mixture model [GMM], hierarchical clustering [HC Euclidean], $k$-means [KM Euclidean], non-negative matrix factorization [NMF Brunet and NMF Lee], partitioning around medoids [PAM Euclidean], spectral clustering using radial-basis kernel function [SC], and self-organizing map with hierarchical clustering [SOM]) of the "analyzer" COPUS codes.

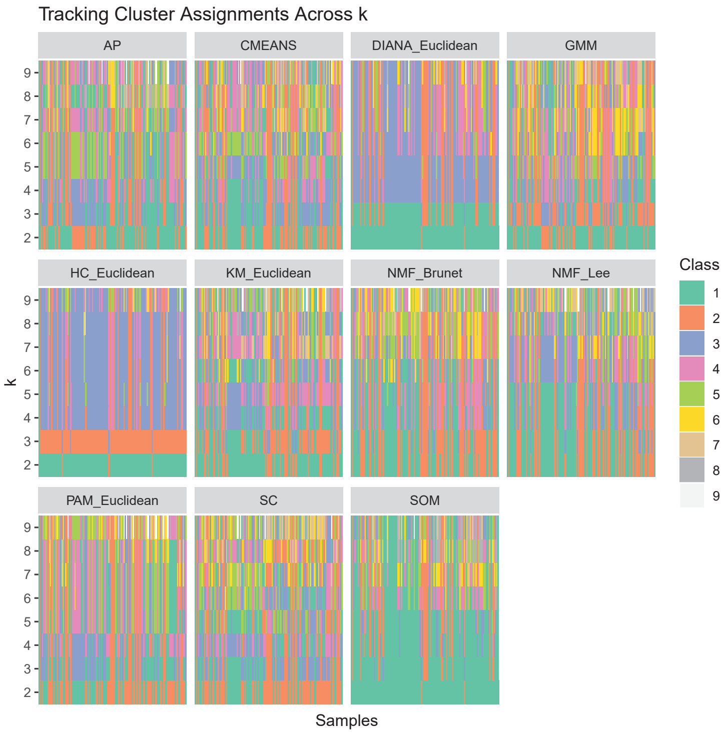


**Fig. S51** Tracking cluster assignments across $k$ ($k = 2, \ldots, 9$) for different clustering algorithms (affinity propagation [AP], fuzzy $C$-means clustering [CMEANS], divisive analysis clustering [DIANA], Gaussian mixture model [GMM], hierarchical clustering [HC Euclidean], $k$-means [KM Euclidean], non-negative matrix factorization [NMF Brunet and NMF Lee], partitioning around medoids [PAM Euclidean], spectral clustering using radial-basis kernel function [SC], and self-organizing map with hierarchical clustering [SOM]) of the "collapsed" COPUS codes.

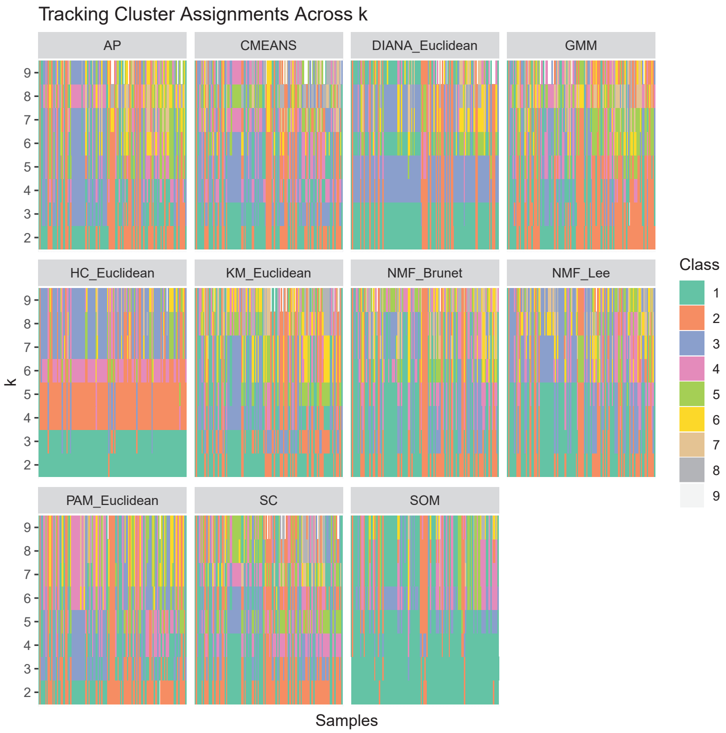


**Fig. S52** Tracking cluster assignments across $k$ ($k = 2, \ldots, 9$) for different clustering algorithms (affinity propagation [AP], fuzzy $C$-means clustering [CMEANS], divisive analysis clustering [DIANA], Gaussian mixture model [GMM], hierarchical clustering [HC Euclidean], $k$-means [KM Euclidean], non-negative matrix factorization [NMF Brunet and NMF Lee], partitioning around medoids [PAM Euclidean], spectral clustering using radial-basis kernel function [SC], and self-organizing map with hierarchical clustering [SOM]) of the "novel" COPUS codes.

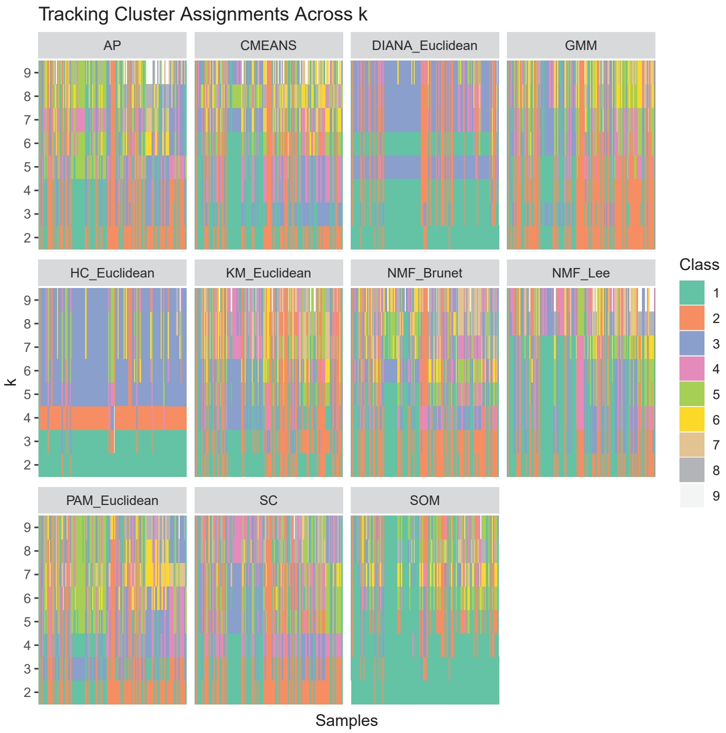


**Fig. S53** Tracking cluster assignments across $k$ ($k = 2, \ldots, 9$) for different clustering algorithms (affinity propagation [AP], fuzzy $C$-means clustering [CMEANS], divisive analysis clustering [DIANA], Gaussian mixture model [GMM], hierarchical clustering [HC Euclidean], $k$-means [KM Euclidean], non-negative matrix factorization [NMF Brunet and NMF Lee], partitioning around medoids [PAM Euclidean], spectral clustering using radial-basis kernel function [SC], and self-organizing map with hierarchical clustering [SOM]) of the “unique” COPUS codes.

# 4 Supplemental Tables

**Table S1** Internal Indices. A list of internal indices used to evaluate the clustering algorithms, the best number of clusters within each algorithm, and the fit of the ensemble clusters. These indices were used to find the optimal cluster size ($k = 2, \ldots, 9$) for the 11 different clustering algorithms ($k$-means, partitioning around medoids, non-negative matrix factorization using euclidean distance, hierarchical clustering, divisive analysis clustering, affinity propagation, spectral clustering using radial-basis kernel function, Gaussian mixture model, self-organizing map with hierarchical clustering, fuzzy $C$-means clustering, and hierarchical density-based spatial clustering of applications with noise) and the best choice of the ensemble algorithms (cluster-based similarity partitioning algorithm, $k$-modes, majority voting, and linkage clustering ensemble).

| Index | diceR Notation | Reference | Minimized/Maximized |
|---|---|---|---|
| 1. CH index | calinski_harabasz | (Caliński & Harabasz, 1974) | Maximized |
| 2. Dunn index | dunn | (Dunn, 1974) | Maximized |
| 3. PBM index | pbm | (Pakhira et al., 2004) | Maximized |
| 4. Tau index | tau | (Milligan, 1981; Rohlf, 1974) | Maximized |
| 5. Gamma index | gamma | (Baker & Hubert, 1975) | Maximized |
| 6. C-index | c_index | (L. Hubert & Schultz, 1976; L.J. Hubert & Levin, 1976) | Minimized |
| 7. DB index | davies_boudlin | (Davies & Bouldin, 1979) | Minimized |
| 8. McClain index | mcclain_rao | (McClain & Rao, 1975) | Minimized |
| 9. SD_Dis index | sd_dis | (Halkidi et al., 2001) | Minimized |
| 10. Ray-Turi index | ray_turi | (Ray & Turi, 1999) | Minimized |
| 11. Gplus index | g_plus | (Rohlf, 1974) | Minimized |
| 12. Silhouette index | silhouette | (Rousseeuw, 1987) | Minimized |
| 13. SDbw index | s_dbw | (Halkidi & Vazirgiannis, 2001) | Minimized |
| 14. Compactness | compactness | (Iam-on & Garrett, 2010) | Minimized |
| 15. Connectivity | connectivity | (Handl et al., 2005) | Minimized |

**Table S2** Summary statistics of each of the standardized percentage of time spent on each COPUS code by faculty type. For each of the standardized codes we scaled the variables to have mean 0 and standard deviation 1. The mean and standard deviation (in parentheses) are given as well as the F statistic and p-value for testing if there is a difference in the amount of time spent on a code across the faculty types. Significance is denoted for codes using a Bonferroni correction of $\alpha^* = 0.05/38 = 0.0013$.

| | | Tenure-track | | | | | |
|---|---|---|---|---|---|---|---|
| | | Yes | Yes | No | | | |
| Dataset | Code | Teaching Faculty | Research Faculty | Lecturers | F | p-value | |
| 1,3,4,5 | Student.L/ S.Receiving/ S.Minimal | -0.43 (1.12) | 0.56 (0.45) | 0.43 (0.77) | 5.04 | 0.01 | |
| 1,5 | Student.AnQ | 0.17 (1.15) | -0.58 (0.80) | -0.09 (1.40) | 2.14 | 0.12 | |
| 1,5 | Student.WC | -0.38 (0.23) | -0.38 (0.12) | -0.38 (0.25) | 1.00 | 0.37 | |
| 1,5 | Student.SP | -0.17 (0.00) | -0.17 (0.00) | -0.17 (0.00) | 0.50 | 0.61 | |
| 1,5 | Student.Ind | 0.38 (1.53) | -0.63 (0.58) | -0.44 (1.12) | 9.79 | <0.001 | * |
| 1,5 | Student.Prd | -0.21 (0.00) | -0.21 (0.00) | -0.21 (0.00) | 1.55 | 0.22 | |
| 1,5 | Student.TQ | -0.28 (0.00) | -0.28 (0.00) | -0.28 (0.00) | 1.18 | 0.31 | |
| 1,5 | Student.W | -0.77 (0.62) | -0.01 (1.23) | -0.52 (1.23) | 2.20 | 0.12 | |
| 1,5 | Student.O | -0.41 (0.37) | -0.41 (0.39) | -0.41 (0.25) | 1.00 | 0.37 | |
| 1,2,5 | Student.SQ | -0.02 (1.22) | -0.38 (0.86) | -0.43 (0.82) | 1.55 | 0.22 | |
| 1,2,5 | Student.CG | -0.35 (1.26) | -0.57 (0.21) | -0.57 (1.10) | 1.75 | 0.18 | |
| 1,2,5 | Student.WG | -0.33 (0.85) | -0.48 (0.18) | -0.48 (0.00) | 1.96 | 0.14 | |
| 1,2,5 | Student.OG | -0.12 (0.90) | -0.54 (0.07) | -0.54 (0.46) | 5.87 | <0.001 | * |
| 3,5 | S.Working | 0.77 (1.13) | -0.74 (1.05) | -0.12 (1.12) | 14.50 | <0.001 | * |
| 3,5 | S.Talking | 0.10 (1.32) | -0.51 (1.14) | -0.11 (1.04) | 2.47 | 0.09 | |
| 3,4,5 | S.Other | -0.35 (0.76) | -0.14 (0.63) | -0.35 (0.90) | 0.17 | 0.85 | |
| 4,5 | S.Interactive | 0.49 (1.51) | -0.80 (0.91) | -0.17 (1.16) | 10.92 | <0.001 | * |
| 4,5 | S.Thinking | 0.50 (1.62) | -0.60 (0.74) | -0.49 (1.07) | 9.46 | <0.001 | * |
| 4,5 | S.Few | 0.12 (1.18) | -0.48 (1.18) | -0.14 (1.06) | 2.07 | 0.13 | |
| 1,5 | Instructor.RtW | -0.50 (0.86) | -0.54 (1.83) | -0.08 (1.12) | 1.30 | 0.28 | |
| 1,5 | Instructor.DV | -0.51 (0.76) | -0.51 (1.00) | -0.34 (1.00) | 0.15 | 0.86 | |
| 1,5 | Instructor.FUp | 0.12 (1.22) | -0.51 (0.83) | -0.08 (1.19) | 5.89 | <0.001 | * |
| 1,5 | Instructor.AnQ | 0.01 (1.25) | -0.39 (0.90) | -0.36 (0.80) | 1.66 | 0.20 | |
| 1,5 | Instructor.MG | 0.08 (1.50) | -0.65 (0.35) | -0.65 (0.84) | 11.32 | <0.001 | * |
| 1,3,5 | Instructor.Adm/ I.Administration | -0.03 (1.01) | -0.31 (1.07) | -0.18 (0.95) | 1.92 | 0.15 | |
| 1,5 | Instructor.W | -0.47 (0.53) | -0.48 (0.50) | -0.47 (0.52) | 0.02 | 0.98 | |
| 1,5 | Instructor.O | -0.38 (0.73) | -0.51 (0.49) | -0.51 (0.31) | 0.58 | 0.56 | |
| 1,2,5 | Instructor.Lec | -0.61 (1.18) | 0.62 (0.87) | 0.05 (1.07) | 7.96 | <0.001 | * |
| 1,2,5 | Instructor.PQ | -0.01 (0.98) | -0.69 (0.96) | -0.12 (1.13) | 1.83 | 0.16 | |
| 1,2,5 | Instructor.CQ | -0.20 (1.23) | -0.66 (0.58) | -0.66 (0.99) | 2.33 | 0.10 | |
| 1,2,5 | Instructor.1o1 | -0.13 (0.67) | -0.4 (0.00) | -0.40 (0.35) | 5.78 | <0.001 | * |
| 3,4,5 | I.Presenting/ I.Minimal | -0.35 (1.10) | 0.56 (0.64) | 0.25 (0.80) | 6.24 | <0.001 | * |
| 3,5 | I.Guiding | 0.82 (0.92) | -0.51 (1.44) | 0.04 (0.92) | 14.26 | <0.001 | * |
| 3,5 | I.Other | -0.31 (0.87) | -0.49 (0.70) | -0.35 (0.72) | 0.17 | 0.84 | |
| 4,5 | I.Interactive | 0.11 (1.44) | -0.68 (0.40) | -0.64 (0.84) | 11.68 | <0.001 | * |
| 4,5 | I.Thinking | 0.42 (1.15) | -0.6 (1.25) | 0.14 (1.22) | 4.27 | 0.02 | |
| 4,5 | I.Few | 0.39 (1.09) | -0.37 (1.18) | -0.20 (1.18) | 7.46 | <0.001 | * |
| 4,5 | I.Miscellaneous | -0.02 (1.20) | -0.48 (0.94) | -0.19 (1.09) | 0.81 | 0.45 | |

**Table S3** CH Index. The CH index for the 11 clustering algorithms and 4 ensembles across the 5 datasets with cluster size $k = 2$. This internal index should be maximized.

| Algorithm | Original | Analyzer | Collapsed | Novel | Unique |
|---|---|---|---|---|---|
| 1. NMF (Brunet) | 20.98 | 28.98 | 58.26 | 54.78 | 36.53 |
| 2. NMF (Lee) | 21.43 | 0.37 | 58.47 | 55.08 | 37.02 |
| 3. $k$-means | 13.33 | 27.25 | 50.68 | 46.16 | 29.66 |
| 4. PAM | 18.17 | 28.30 | 53.71 | 48.82 | 33.21 |
| 5. HC | 5.95 | 24.49 | 9.10 | 5.54 | 7.31 |
| 6. DIANA | 5.95 | 31.91 | 8.66 | 46.28 | 19.04 |
| 7. AP | 16.29 | 31.18 | 43.55 | 51.61 | 33.58 |
| 8. SC | 6.49 | 24.49 | 49.44 | 46.78 | 32.96 |
| 9. GMM | 14.42 | 25.72 | 34.31 | 43.08 | 29.62 |
| 10. SOM | 5.95 | 24.49 | 8.66 | 13.68 | 7.31 |
| 11. $C$-means | 21.72 | 33.17 | 58.79 | 55.27 | 37.19 |
| **Ensemble** | | | | | |
| 1. $k$-modes | 5.95 | 24.49 | 55.62 | 52.13 | 37.19 |
| 2. Majority Voting | – | 24.49 | 43.52 | 45.29 | 19.93 |
| 3. CSPA | 21.60 | 24.49 | 58.95 | 55.27 | 37.12 |
| 4. LCE | 12.34 | 24.49 | 6.87 | 48.40 | 7.31 |

**Table S4** Dunn Index. The Dunn index for the 11 clustering algorithms and 4 ensembles across the 5 datasets with cluster size $k = 2$. This internal index should be maximized.

| Algorithm | Original | Analyzer | Collapsed | Novel | Unique |
|---|---|---|---|---|---|
| 1. NMF (Brunet) | 0.12 | 0.08 | 0.09 | 0.14 | 0.12 |
| 2. NMF (Lee) | 0.17 | 0.15 | 0.08 | 0.14 | 0.19 |
| 3. $k$-means | 0.21 | 0.14 | 0.11 | 0.06 | 0.09 |
| 4. PAM | 0.08 | 0.10 | 0.07 | 0.06 | 0.12 |
| 5. HC | 0.64 | 0.40 | 0.42 | 0.49 | 0.47 |
| 6. DIANA | 0.64 | 0.19 | 0.40 | 0.19 | 0.34 |
| 7. AP | 0.08 | 0.05 | 0.07 | 0.11 | 0.12 |
| 8. SC | 0.21 | 0.40 | 0.07 | 0.09 | 0.12 |
| 9. GMM | 0.15 | 0.06 | 0.04 | 0.09 | 0.13 |
| 10. SOM | 0.64 | 0.40 | 0.40 | 0.18 | 0.47 |
| 11. $C$-means | 0.15 | 0.10 | 0.08 | 0.14 | 0.19 |
| **Ensemble** | | | | | |
| 1. $k$-modes | 0.64 | 0.40 | 0.15 | 0.17 | 0.19 |
| 2. Majority Voting | Inf | 0.40 | 0.11 | 0.21 | 0.26 |
| 3. CSPA | 0.21 | 0.40 | 0.08 | 0.14 | 0.19 |
| 4. LCE | 0.27 | 0.40 | 0.50 | 0.13 | 0.47 |

**Table S5** PBM Index. The PBM index for the 11 clustering algorithms and 4 ensembles across the 5 datasets with cluster size $k = 2$. This internal index should be maximized.

| Algorithm | Original | Analyzer | Collapsed | Novel | Unique |
|---|---|---|---|---|---|
| 1. NMF (Brunet) | 4.30 | 2.12 | 3.86 | 4.56 | 14.47 |
| 2. NMF (Lee) | 4.59 | 0.76 | 3.91 | 4.61 | 14.87 |
| 3. $k$-means | 3.40 | 2.24 | 4.33 | 4.20 | 12.96 |
| 4. PAM | 3.81 | 2.25 | 3.64 | 4.29 | 13.64 |
| 5. HC | 37.59 | 12.32 | 6.09 | 14.03 | 44.18 |
| 6. DIANA | 37.59 | 5.34 | 8.69 | 5.17 | 29.91 |
| 7. AP | 3.47 | 2.05 | 3.47 | 4.34 | 13.71 |
| 8. SC | 10.54 | 12.32 | 3.60 | 4.19 | 13.80 |
| 9. GMM | 3.35 | 1.88 | 3.20 | 3.82 | 13.05 |
| 10. SOM | 37.59 | 12.32 | 8.69 | 5.11 | 44.18 |
| 11. $C$-means | 4.56 | 2.26 | 3.91 | 4.63 | 14.75 |
| Ensemble | | | | | |
| 1. $k$-modes | 37.59 | 12.32 | 4.64 | 4.89 | 14.75 |
| 2. Majority Voting | Inf | 12.32 | 4.73 | 5.18 | 28.10 |
| 3. CSPA | 4.58 | 12.32 | 3.93 | 4.63 | 14.76 |
| 4. LCE | 6.18 | 12.32 | 13.85 | 4.47 | 44.18 |

**Table S6** Tau Index. The Tau index for the 11 clustering algorithms and 4 ensembles across the 5 datasets with cluster size $k = 2$. This internal index should be maximized.

| Algorithm | Original | Analyzer | Collapsed | Novel | Unique |
|---|---|---|---|---|---|
| 1. NMF (Brunet) | 0.24 | 0.34 | 0.38 | 0.38 | 0.31 |
| 2. NMF (Lee) | 0.31 | -0.03 | 0.39 | 0.40 | 0.37 |
| 3. $k$-means | 0.51 | 0.53 | 0.53 | 0.50 | 0.49 |
| 4. PAM | 0.13 | 0.41 | 0.30 | 0.29 | 0.22 |
| 5. HC | 0.17 | 0.32 | 0.26 | 0.17 | 0.23 |
| 6. DIANA | 0.17 | 0.38 | 0.21 | 0.48 | 0.41 |
| 7. AP | 0.11 | 0.25 | 0.19 | 0.36 | 0.23 |
| 8. SC | 0.19 | 0.32 | 0.25 | 0.28 | 0.21 |
| 9. GMM | -0.01 | 0.14 | 0.11 | 0.25 | 0.14 |
| 10. SOM | 0.17 | 0.32 | 0.21 | 0.37 | 0.23 |
| 11. $C$-means | 0.31 | 0.31 | 0.40 | 0.40 | 0.36 |
| Ensemble | | | | | |
| 1. $k$-modes | 0.17 | 0.32 | 0.52 | 0.48 | 0.36 |
| 2. Majority Voting | – | 0.32 | 0.48 | 0.48 | 0.41 |
| 3. CSPA | 0.32 | 0.32 | 0.40 | 0.40 | 0.36 |
| 4. LCE | 0.44 | 0.32 | 0.17 | 0.50 | 0.23 |

**Table S7** Gamma Index. The Gamma index for the 11 clustering algorithms and 4 ensembles across the 5 datasets with cluster size $k = 2$. This internal index should be maximized.

| Algorithm | Original | Analyzer | Collapsed | Novel | Unique |
|---|---|---|---|---|---|
| 1. NMF (Brunet) | 0.33 | 0.49 | 0.53 | 0.54 | 0.44 |
| 2. NMF (Lee) | 0.44 | -0.16 | 0.56 | 0.57 | 0.53 |
| 3. $k$-means | 0.74 | 0.76 | 0.77 | 0.70 | 0.69 |
| 4. PAM | 0.19 | 0.58 | 0.42 | 0.41 | 0.31 |
| 5. HC | 0.99 | 0.95 | 0.88 | 0.94 | 0.93 |
| 6. DIANA | 0.99 | 0.69 | 0.84 | 0.70 | 0.90 |
| 7. AP | 0.16 | 0.36 | 0.27 | 0.51 | 0.32 |
| 8. SC | 0.57 | 0.95 | 0.36 | 0.40 | 0.30 |
| 9. GMM | -0.02 | 0.19 | 0.16 | 0.35 | 0.20 |
| 10. SOM | 0.99 | 0.95 | 0.84 | 0.85 | 0.93 |
| 11. $C$-means | 0.44 | 0.44 | 0.56 | 0.57 | 0.51 |
| Ensemble | | | | | |
| 1. $k$-modes | 0.99 | 0.95 | 0.75 | 0.69 | 0.51 |
| 2. Majority Voting | – | 0.95 | 0.75 | 0.71 | 0.85 |
| 3. CSPA | 0.46 | 0.95 | 0.57 | 0.57 | 0.51 |
| 4. LCE | 0.78 | 0.95 | 0.96 | 0.72 | 0.93 |

**Table S8** $C$ Index. The $C$ index for the 11 clustering algorithms and 4 ensembles across the 5 datasets with cluster size $k = 2$. This internal index should be minimized.

| Algorithm | Original | Analyzer | Collapsed | Novel | Unique |
|---|---|---|---|---|---|
| 1. NMF (Brunet) | 0.33 | 0.26 | 0.22 | 0.22 | 0.27 |
| 2. NMF (Lee) | 0.28 | 0.65 | 0.21 | 0.21 | 0.23 |
| 3. $k$-means | 0.15 | 0.14 | 0.11 | 0.15 | 0.16 |
| 4. PAM | 0.40 | 0.22 | 0.28 | 0.29 | 0.34 |
| 5. HC | 0.03 | 0.05 | 0.14 | 0.13 | 0.10 |
| 6. DIANA | 0.03 | 0.20 | 0.19 | 0.15 | 0.08 |
| 7. AP | 0.42 | 0.31 | 0.37 | 0.24 | 0.33 |
| 8. SC | 0.33 | 0.05 | 0.32 | 0.30 | 0.35 |
| 9. GMM | 0.51 | 0.41 | 0.44 | 0.32 | 0.40 |
| 10. SOM | 0.03 | 0.05 | 0.19 | 0.12 | 0.10 |
| 11. $C$-means | 0.28 | 0.28 | 0.21 | 0.21 | 0.24 |
| Ensemble | | | | | |
| 1. $k$-modes | 0.03 | 0.05 | 0.12 | 0.16 | 0.24 |
| 2. Majority Voting | – | 0.05 | 0.13 | 0.15 | 0.10 |
| 3. CSPA | 0.27 | 0.05 | 0.21 | 0.21 | 0.24 |
| 4. LCE | 0.13 | 0.05 | 0.06 | 0.14 | 0.10 |

**Table S9** DB Index. The DB index for the 11 clustering algorithms and 4 ensembles across the 5 datasets with cluster size $k = 2$. This internal index should be minimized.

| Algorithm | Original | Analyzer | Collapsed | Novel | Unique |
|---|---|---|---|---|---|
| 1. NMF (Brunet) | 2.25 | 1.87 | 1.34 | 1.39 | 1.70 |
| 2. NMF (Lee) | 2.27 | 1.47 | 1.34 | 1.40 | 1.74 |
| 3. $k$-means | 2.92 | 2.03 | 1.39 | 1.55 | 1.97 |
| 4. PAM | 2.31 | 1.90 | 1.32 | 1.35 | 1.68 |
| 5. HC | 0.38 | 0.61 | 1.36 | 0.39 | 1.03 |
| 6. DIANA | 0.38 | 1.17 | 0.83 | 1.39 | 1.37 |
| 7. AP | 2.41 | 1.81 | 1.22 | 1.40 | 1.69 |
| 8. SC | 1.32 | 0.61 | 1.26 | 1.35 | 1.65 |
| 9. GMM | 2.27 | 1.70 | 1.17 | 1.41 | 1.63 |
| 10. SOM | 0.38 | 0.61 | 0.83 | 1.66 | 1.03 |
| 11. $C$-means | 2.26 | 1.74 | 1.34 | 1.39 | 1.73 |
| Ensemble | | | | | |
| 1. $k$-modes | 0.38 | 0.61 | 1.32 | 1.41 | 1.73 |
| 2. Majority Voting | 0.00 | 0.61 | 1.33 | 1.39 | 1.39 |
| 3. CSPA | 2.27 | 0.61 | 1.34 | 1.39 | 1.73 |
| 4. LCE | 2.28 | 0.61 | 0.35 | 1.50 | 1.03 |

**Table S10** McClain Index. The McClain index for the 11 clustering algorithms and 4 ensembles across the 5 datasets with cluster size $k = 2$. This internal index should be minimized.

| Algorithm | Original | Analyzer | Collapsed | Novel | Unique |
|---|---|---|---|---|---|
| 1. NMF (Brunet) | 0.83 | 0.69 | 0.67 | 0.69 | 0.77 |
| 2. NMF (Lee) | 0.78 | 1.15 | 0.66 | 0.67 | 0.73 |
| 3. $k$-means | 0.68 | 0.57 | 0.57 | 0.62 | 0.67 |
| 4. PAM | 0.90 | 0.65 | 0.73 | 0.76 | 0.83 |
| 5. HC | 0.51 | 0.47 | 0.51 | 0.51 | 0.56 |
| 6. DIANA | 0.51 | 0.60 | 0.54 | 0.62 | 0.58 |
| 7. AP | 0.91 | 0.75 | 0.82 | 0.71 | 0.82 |
| 8. SC | 0.75 | 0.47 | 0.77 | 0.77 | 0.84 |
| 9. GMM | 1.01 | 0.87 | 0.88 | 0.79 | 0.89 |
| 10. SOM | 0.51 | 0.47 | 0.54 | 0.55 | 0.56 |
| 11. $C$-means | 0.78 | 0.72 | 0.66 | 0.67 | 0.74 |
| Ensemble | | | | | |
| 1. $k$-modes | 0.51 | 0.47 | 0.58 | 0.63 | 0.74 |
| 2. Majority Voting | – | 0.47 | 0.57 | 0.62 | 0.59 |
| 3. CSPA | 0.77 | 0.47 | 0.66 | 0.67 | 0.74 |
| 4. LCE | 0.65 | 0.47 | 0.46 | 0.62 | 0.56 |

**Table S11** SD_Dis Index. The SD_Dis index for the 11 clustering algorithms and 4 ensembles across the 5 datasets with cluster size $k = 2$. This internal index should be minimized.

| Algorithm | Original | Analyzer | Collapsed | Novel | Unique |
|---|---|---|---|---|---|
| 1. NMF (Brunet) | 0.53 | 0.77 | 0.63 | 0.57 | 0.30 |
| 2. NMF (Lee) | 0.51 | 1.16 | 0.62 | 0.57 | 0.30 |
| 3. $k$-means | 0.57 | 0.74 | 0.57 | 0.58 | 0.31 |
| 4. PAM | 0.56 | 0.75 | 0.64 | 0.59 | 0.31 |
| 5. HC | 0.17 | 0.31 | 0.41 | 0.27 | 0.15 |
| 6. DIANA | 0.17 | 0.48 | 0.35 | 0.51 | 0.19 |
| 7. AP | 0.58 | 0.79 | 0.65 | 0.59 | 0.31 |
| 8. SC | 0.32 | 0.31 | 0.65 | 0.60 | 0.31 |
| 9. GMM | 0.59 | 0.84 | 0.66 | 0.62 | 0.32 |
| 10. SOM | 0.17 | 0.31 | 0.35 | 0.46 | 0.15 |
| 11. $C$-means | 0.51 | 0.77 | 0.62 | 0.57 | 0.30 |
| Ensemble | | | | | |
| 1. $k$-modes | 0.17 | 0.31 | 0.56 | 0.54 | 0.30 |
| 2. Majority Voting | – | 0.31 | 0.53 | 0.51 | 0.20 |
| 3. CSPA | 0.51 | 0.31 | 0.62 | 0.57 | 0.30 |
| 4. LCE | 0.42 | 0.31 | 0.27 | 0.56 | 0.15 |

**Table S12** Ray-Turi Index. The Ray-Turi index for the 11 clustering algorithms and 4 ensembles across the 5 datasets with cluster size $k = 2$. This internal index should be minimized.

| Algorithm | Original | Analyzer | Collapsed | Novel | Unique |
|---|---|---|---|---|---|
| 1. NMF (Brunet) | 1.49 | 0.95 | 0.53 | 0.57 | 0.86 |
| 2. NMF (Lee) | 1.37 | 2.64 | 0.52 | 0.55 | 0.82 |
| 3. $k$-means | 1.84 | 0.90 | 0.46 | 0.61 | 0.93 |
| 4. PAM | 1.69 | 0.91 | 0.58 | 0.63 | 0.92 |
| 5. HC | 0.16 | 0.16 | 0.32 | 0.18 | 0.27 |
| 6. DIANA | 0.16 | 0.36 | 0.22 | 0.48 | 0.39 |
| 7. AP | 1.87 | 0.99 | 0.63 | 0.61 | 0.91 |
| 8. SC | 0.59 | 0.16 | 0.60 | 0.65 | 0.92 |
| 9. GMM | 1.94 | 1.17 | 0.69 | 0.70 | 0.97 |
| 10. SOM | 0.16 | 0.16 | 0.22 | 0.48 | 0.27 |
| 11. $C$-means | 1.39 | 0.93 | 0.52 | 0.56 | 0.83 |
| Ensemble | | | | | |
| 1. $k$-modes | 0.16 | 0.16 | 0.42 | 0.51 | 0.83 |
| 2. Majority Voting | 0.00 | 0.16 | 0.41 | 0.47 | 0.41 |
| 3. CSPA | 1.38 | 0.16 | 0.52 | 0.56 | 0.83 |
| 4. LCE | 0.99 | 0.16 | 0.14 | 0.56 | 0.27 |

**Table S13** Gplus Index. The Gplus index for the 11 clustering algorithms and 4 ensembles across the 5 datasets with cluster size $k = 2$. This internal index should be minimized.

| Algorithm | Original | Analyzer | Collapsed | Novel | Unique |
|---|---|---|---|---|---|
| 1. NMF (Brunet) | 0.17 | 0.13 | 0.12 | 0.11 | 0.14 |
| 2. NMF (Lee) | 0.14 | 0.02 | 0.11 | 0.11 | 0.12 |
| 3. $k$-means | 0.06 | 0.06 | 0.05 | 0.07 | 0.08 |
| 4. PAM | 0.20 | 0.10 | 0.14 | 0.15 | 0.17 |
| 5. HC | 0.00 | 0.00 | 0.01 | 0.00 | 0.00 |
| 6. DIANA | 0.00 | 0.05 | 0.00 | 0.07 | 0.01 |
| 7. AP | 0.21 | 0.16 | 0.18 | 0.12 | 0.17 |
| 8. SC | 0.02 | 0.00 | 0.16 | 0.15 | 0.18 |
| 9. GMM | 0.25 | 0.20 | 0.20 | 0.16 | 0.20 |
| 10. SOM | 0.00 | 0.00 | 0.00 | 0.01 | 0.00 |
| 11. $C$-means | 0.14 | 0.14 | 0.11 | 0.11 | 0.12 |
| Ensemble | | | | | |
| 1. $k$-modes | 0.00 | 0.00 | 0.06 | 0.08 | 0.12 |
| 2. Majority Voting | 0.00 | 0.00 | 0.05 | 0.07 | 0.02 |
| 3. CSPA | 0.14 | 0.00 | 0.11 | 0.11 | 0.12 |
| 4. LCE | 0.03 | 0.00 | 0.00 | 0.07 | 0.00 |

**Table S14** Silhouette Index. The Silhouette index for the 11 clustering algorithms and 4 ensembles across the 5 datasets with cluster size $k = 2$. This internal index should be minimized.

| Algorithm | Original | Analyzer | Collapsed | Novel | Unique |
|---|---|---|---|---|---|
| 1. NMF (Brunet) | 0.16 | 0.21 | 0.28 | 0.28 | 0.22 |
| 2. NMF (Lee) | 0.15 | – | 0.28 | 0.27 | 0.21 |
| 3. $k$-means | 0.13 | 0.21 | 0.28 | 0.25 | 0.19 |
| 4. PAM | 0.15 | 0.20 | 0.29 | 0.29 | 0.22 |
| 5. HC | – | 0.57 | 0.23 | – | 0.28 |
| 6. DIANA | – | 0.33 | 0.38 | 0.28 | 0.28 |
| 7. AP | 0.15 | 0.20 | 0.32 | 0.27 | 0.22 |
| 8. SC | 0.26 | 0.57 | 0.31 | 0.29 | 0.23 |
| 9. GMM | 0.17 | 0.25 | 0.34 | 0.27 | 0.24 |
| 10. SOM | – | 0.57 | 0.38 | 0.23 | 0.28 |
| 11. $C$-means | 0.15 | 0.22 | 0.28 | 0.27 | 0.21 |
| Ensemble | | | | | |
| 1. $k$-modes | – | 0.57 | 0.30 | 0.27 | 0.21 |
| 2. Majority Voting | 1.00 | 0.57 | 0.29 | 0.27 | 0.27 |
| 3. CSPA | 0.15 | 0.57 | 0.28 | 0.27 | 0.21 |
| 4. LCE | 0.15 | 0.57 | – | 0.26 | 0.28 |

**Table S15** SDbw Index. The SDbw index for the 11 clustering algorithms and 4 ensembles across the 5 datasets with cluster size $k = 2$. This internal index should be minimized.

| Algorithm | Original | Analyzer | Collapsed | Novel | Unique |
|---|---|---|---|---|---|
| 1. NMF (Brunet) | – | 1.18 | 1.03 | 0.81 | – |
| 2. NMF (Lee) | – | 0.50 | 1.21 | 0.82 | – |
| 3. *k*-means | – | 1.28 | 1.44 | 0.91 | – |
| 4. PAM | 0.96 | 1.08 | 1.00 | 0.71 | – |
| 5. HC | 0.49 | – | 2.19 | 0.49 | – |
| 6. DIANA | 0.49 | 1.23 | 0.99 | 0.91 | – |
| 7. AP | – | 1.18 | 0.69 | 0.77 | – |
| 8. SC | – | – | 0.81 | 0.76 | – |
| 9. GMM | – | 0.95 | 0.64 | 0.72 | – |
| 10. SOM | 0.49 | – | 0.99 | 2.14 | – |
| 11. *C*-means | – | 1.05 | 1.04 | 0.81 | – |
| Ensemble | | | | | |
| 1. *k*-modes | 0.49 | – | 1.28 | 0.87 | – |
| 2. Majority Voting | 1.00 | – | 1.39 | 0.92 | – |
| 3. CSPA | – | – | 1.13 | 0.81 | – |
| 4. LCE | – | – | 0.48 | 0.92 | – |

**Table S16** Compactness. The measure of compactness for the 11 clustering algorithms and 4 ensembles across the 5 datasets with cluster size $k = 2$. This internal index should be minimized.

| Algorithm | Original | Analyzer | Collapsed | Novel | Unique |
|---|---|---|---|---|---|
| 1. NMF (Brunet) | 6.10 | 3.20 | 2.99 | 3.41 | 8.01 |
| 2. NMF (Lee) | 6.14 | 3.60 | 3.00 | 3.42 | 8.07 |
| 3. *k*-means | 6.29 | 3.21 | 3.09 | 3.50 | 8.23 |
| 4. PAM | 6.15 | 3.23 | 3.00 | 3.41 | 8.05 |
| 5. HC | 6.53 | 3.36 | 3.57 | 4.03 | 9.06 |
| 6. DIANA | 6.53 | 3.27 | 3.56 | 3.56 | 8.71 |
| 7. AP | 6.18 | 3.18 | 3.04 | 3.41 | 8.04 |
| 8. SC | 6.54 | 3.36 | 3.00 | 3.41 | 8.01 |
| 9. GMM | 6.17 | 3.13 | 3.11 | 3.46 | 8.07 |
| 10. SOM | 6.53 | 3.36 | 3.56 | 3.95 | 9.06 |
| 11. *C*-means | 6.11 | 3.12 | 2.99 | 3.41 | 8.03 |
| Ensemble | | | | | |
| 1. *k*-modes | 6.53 | 3.36 | 3.06 | 3.48 | 8.03 |
| 2. Majority Voting | 6.68 | 3.36 | 3.18 | 3.57 | 8.68 |
| 3. CSPA | 6.12 | 3.36 | 2.99 | 3.41 | 8.05 |
| 4. LCE | 6.40 | 3.36 | 3.55 | 3.49 | 9.06 |

**Table S17** Connectivity. The measure of connectivity for the 11 clustering algorithms and 4 ensembles across the 5 datasets with cluster size $k = 2$. This internal index should be minimized.

| Algorithm | Original | Analyzer | Collapsed | Novel | Unique |
|---|---|---|---|---|---|
| 1. NMF (Brunet) | 57.48 | 31.19 | 39.94 | 35.53 | 40.99 |
| 2. NMF (Lee) | 50.80 | 4.96 | 44.30 | 39.46 | 44.79 |
| 3. $k$-means | 74.03 | 37.69 | 33.85 | 49.79 | 63.88 |
| 4. PAM | 57.34 | 53.00 | 28.33 | 40.21 | 45.43 |
| 5. HC | 2.93 | 4.38 | 7.79 | 2.93 | 5.61 |
| 6. DIANA | 2.93 | 13.57 | 4.86 | 31.41 | 14.69 |
| 7. AP | 60.34 | 38.17 | 27.85 | 40.26 | 47.14 |
| 8. SC | 8.62 | 4.38 | 29.41 | 25.85 | 38.30 |
| 9. GMM | 66.25 | 31.38 | 30.70 | 39.55 | 41.47 |
| 10. SOM | 2.93 | 4.38 | 4.86 | 17.92 | 5.61 |
| 11. $C$-means | 52.71 | 36.56 | 43.34 | 37.10 | 47.54 |
| Ensemble | | | | | |
| 1. $k$-modes | 2.93 | 4.38 | 30.93 | 41.71 | 47.54 |
| 2. Majority Voting | 0.00 | 4.38 | 33.81 | 30.56 | 15.79 |
| 3. CSPA | 52.11 | 4.38 | 45.57 | 37.10 | 46.43 |
| 4. LCE | 26.69 | 4.38 | 2.93 | 46.78 | 5.61 |

**Table S18** Best Ensemble. The best ensemble (cluster-based similarity partitioning algorithm [CSPA], $k$-modes, majority voting, or linkage clustering ensemble [LCE]) for each dataset was found based on the internal validity indices and having balanced cluster sizes.

| Dataset | Number of Codes | Best Ensemble |
|---|---|---|
| 1. Original Codes | 25 | CSPA |
| 2. Analyzer Codes | 8 | None |
| 3. Collapsed Codes | 8 | $k$-modes |
| 4. Novel Codes | 10 | Majority Voting |
| 5. Unique Codes | 38 | CSPA |

**Table S19** Internal Indices for the Best Choice of Ensemble Algorithm. The internal validity indices for the best algorithm for each of the COPUS datasets. The bold indicates when the internal index optimal. Since the analyzer codes did not produce a best choice of ensemble algorithm, we exclude the internal indices of the analyzer code ensemble.

| | Dataset | | | | |
|---|---|---|---|---|---|
| Index | Original Codes | Collapsed Codes | Novel Codes | Unique Codes | Minimized/Maximized |
| 1. CH index | 21.60 | **55.62** | 45.29 | 37.12 | Maximized |
| 2. Dunn index | **0.21** | 0.15 | 0.21 | 0.19 | Maximized |
| 3. PBM index | 4.58 | 4.64 | 5.18 | **14.76** | Maximized |
| 4. Tau index | 0.32 | **0.52** | 0.48 | 0.36 | Maximized |
| 5. Gamma index | 0.46 | **0.75** | 0.71 | 0.51 | Maximized |
| 6. C-index | 0.27 | **0.12** | 0.15 | 0.24 | Minimized |
| 7. DB index | 2.27 | **1.32** | 1.39 | 1.73 | Minimized |
| 8. McClain index | 0.77 | **0.58** | 0.62 | 0.74 | Minimized |
| 9. SD_Dis index | 0.51 | 0.56 | 0.51 | **0.30** | Minimized |
| 10. Ray-Turi index | 1.38 | **0.42** | 0.47 | 0.83 | Minimized |
| 11. Gplus index | 0.14 | **0.06** | 0.07 | 0.12 | Minimized |
| 12. Silhouette index | **0.15** | 0.30 | 0.27 | 0.21 | Minimized |
| 13. SDbw index | – | 1.28 | **0.92** | – | Minimized |
| 14. Compactness | 6.12 | **3.06** | 3.57 | 8.05 | Minimized |
| 15. Connectivity | 52.11 | 30.93 | **30.56** | 46.43 | Minimized |

**Table S20** Summary Statistics for Faculty Type across the 5 Different Cluster Ensembles and the Final Clusters. Summary of faculty type for the traditional-lecture and active-learning clusters. The number and the conditional percent (given cluster) are presented.

| Dataset | Variable | Traditional Cluster | Active Cluster | All Classes |
|---|---|---|---|---|
| Original | Faculty Type | | | |
| | Teaching Faculty | 18 (22%) | 21 (48%) | 39 (31%) |
| | Research Faculty | 39 (48%) | 13 (30%) | 52 (42%) |
| | Lecturers | 24 (30%) | 10 (23%) | 34 (27%) |
| | | $n_0 = 81$ | $n_1 = 44$ | $n = 125$ |
| Analyzer | Faculty Type | | | |
| | Teaching Faculty | 29 (27%) | 10 (59%) | 39 (31%) |
| | Research Faculty | 49 (45%) | 3 (18%) | 52 (42%) |
| | Lecturers | 30 (28%) | 4 (24%) | 34 (27%) |
| | | $n_0 = 108$ | $n_1 = 17$ | $n = 125$ |
| Collapsed | Faculty Type | | | |
| | Teaching Faculty | 20 (21%) | 19 (61%) | 39 (31%) |
| | Research Faculty | 46 (49%) | 6 (19%) | 52 (42%) |
| | Lecturers | 28 (30%) | 6 (19%) | 34 (27%) |
| | | $n_0 = 94$ | $n_1 = 31$ | $n = 125$ |
| Novel | Faculty Type | | | |
| | Teaching Faculty | 8 (14%) | 31 (46%) | 39 (31%) |
| | Research Faculty | 34 (60%) | 18 (26%) | 52 (42%) |
| | Lecturers | 15 (26%) | 19 (28%) | 34 (27%) |
| | | $n_0 = 57$ | $n_1 = 68$ | $n = 125$ |
| Unique | Faculty Type | | | |
| | Teaching Faculty | 14 (19%) | 25 (47%) | 39 (31%) |
| | Research Faculty | 37 (51%) | 15 (28%) | 52 (42%) |
| | Lecturers | 21 (29%) | 13 (25%) | 34 (27%) |
| | | $n_0 = 72$ | $n_1 = 53$ | $n = 125$ |
| Final Clusters | Faculty Type | | | |
| | Teaching Faculty | 17 (22%) | 22 (47%) | 39 (31%) |
| | Research Faculty | 39 (50%) | 13 (28%) | 52 (42%) |
| | Lecturers | 22 (28%) | 12 (26%) | 34 (27%) |
| | | $n_0 = 78$ | $n_1 = 47$ | $n = 125$ |

**Table S21** Summary Statistics for Faculty Rank across the 5 Different Cluster Ensembles and the Final Clusters. Summary of faculty rank for the traditional-lecture and active-learning clusters. The number and the conditional percent (given cluster) are presented.

| Dataset | Variable | Traditional Cluster | Active Cluster | All Classes |
|---|---|---|---|---|
| Original | Faculty Rank | | | |
| | Assistant | 34 (42%) | 26 (59%) | 60 (48%) |
| | Associate | 18 (22%) | 9 (20%) | 27 (22%) |
| | Full | 29 (36%) | 9 (20%) | 38 (30%) |
| | | $n_0 = 81$ | $n_1 = 44$ | $n = 125$ |
| Analyzer | Faculty Rank | | | |
| | Assistant | 49 (45%) | 11 (65%) | 60 (48%) |
| | Associate | 23 (21%) | 4 (24%) | 27 (22%) |
| | Full | 36 (33%) | 2 (12%) | 38 (30%) |
| | | $n_0 = 108$ | $n_1 = 17$ | $n = 125$ |
| Collapsed | Faculty Rank | | | |
| | Assistant | 38 (40%) | 22 (71%) | 60 (48%) |
| | Associate | 21 (22%) | 6 (19%) | 27 (22%) |
| | Full | 35 (37%) | 3 (10%) | 38 (30%) |
| | | $n_0 = 94$ | $n_1 = 31$ | $n = 125$ |
| Novel | Faculty Rank | | | |
| | Assistant | 21 (37%) | 39 (57%) | 60 (48%) |
| | Associate | 10 (18%) | 17 (25%) | 27 (22%) |
| | Full | 26 (46%) | 12 (18%) | 38 (30%) |
| | | $n_0 = 57$ | $n_1 = 68$ | $n = 125$ |
| Unique | Faculty Rank | | | |
| | Assistant | 29 (40%) | 31 (58%) | 60 (48%) |
| | Associate | 15 (21%) | 12 (23%) | 27 (22%) |
| | Full | 28 (39%) | 10 (19%) | 38 (30%) |
| | | $n_0 = 72$ | $n_1 = 53$ | $n = 125$ |
| Final Clusters | Faculty Rank | | | |
| | Assistant | 32 (41%) | 28 (60%) | 60 (48%) |
| | Associate | 17 (22%) | 10 (21%) | 27 (22%) |
| | Full | 29 (37%) | 9 (19%) | 38 (30%) |
| | | $n_0 = 78$ | $n_1 = 47$ | $n = 125$ |

**Table S22** Summary Statistics for Years of Teaching across the 5 Different Cluster Ensembles and the Final Clusters. Summary of faculty rank for the traditional-lecture and active-learning clusters. The mean and standard deviation (in parentheses) are presented.

| Dataset | Variable | Traditional Cluster | Active Cluster | All Classes |
|---|---|---|---|---|
| Original | Years of Teaching | 9 (6)<br>$n_0 = 81$ | 9 (7)<br>$n_1 = 44$ | 9 (6)<br>$n = 125$ |
| Analyzer | Years of Teaching | 9 (6)<br>$n_0 = 108$ | 10 (7)<br>$n_1 = 17$ | 9 (6)<br>$n = 125$ |
| Collapsed | Years of Teaching | 10 (6)<br>$n_0 = 94$ | 7 (7)<br>$n_1 = 31$ | 9 (6)<br>$n = 125$ |
| Novel | Years of Teaching | 10 (7)<br>$n_0 = 57$ | 8 (6)<br>$n_1 = 68$ | 9 (6)<br>$n = 125$ |
| Unique | Years of Teaching | 9 (6)<br>$n_0 = 72$ | 9 (6)<br>$n_1 = 53$ | 9 (6)<br>$n = 125$ |
| Final Clusters | Years of Teaching | 9 (6)<br>$n_0 = 78$ | 9 (7)<br>$n_1 = 47$ | 9 (6)<br>$n = 125$ |

**Table S23** Summary Statistics for Gender across the 5 Different Cluster Ensembles and the Final Clusters. Summary of gender for the traditional-lecture and active-learning clusters. The number and the conditional percent (given cluster) are presented.

| Dataset | Variable | Traditional Cluster | Active Cluster | All Classes |
|---|---|---|---|---|
| Original | Gender | | | |
| | Female | 36 (44%) | 23 (52%) | 59 (47%) |
| | Non-female | 45 (56%) | 21 (48%) | 66 (53%) |
| | | $n_0 = 81$ | $n_1 = 44$ | $n = 125$ |
| Analyzer | Gender | | | |
| | Female | 50 (46%) | 9 (53%) | 59 (47%) |
| | Non-female | 58 (54%) | 8 (47%) | 66 (53%) |
| | | $n_0 = 108$ | $n_1 = 17$ | $n = 125$ |
| Collapsed | Gender | | | |
| | Female | 41 (44%) | 18 (58%) | 59 (47%) |
| | Non-female | 53 (56%) | 13 (42%) | 66 (53%) |
| | | $n_0 = 94$ | $n_1 = 31$ | $n = 125$ |
| Novel | Gender | | | |
| | Female | 22 (39%) | 37 (54%) | 59 (47%) |
| | Non-female | 35 (61%) | 31 (46%) | 66 (53%) |
| | | $n_0 = 57$ | $n_1 = 68$ | $n = 125$ |
| Unique | Gender | | | |
| | Female | 28 (39%) | 31 (58%) | 59 (47%) |
| | Non-female | 44 (61%) | 22 (42%) | 66 (53%) |
| | | $n_0 = 72$ | $n_1 = 53$ | $n = 125$ |
| Final Clusters | Gender | | | |
| | Female | 33 (42%) | 26 (55%) | 59 (47%) |
| | Non-female | 45 (58%) | 21 (45%) | 66 (53%) |
| | | $n_0 = 78$ | $n_1 = 47$ | $n = 125$ |

**Table S24** Summary Statistics for Campus across the 5 Different Cluster Ensembles and the Final Clusters. Summary of campus for the traditional-lecture and active-learning clusters. The number and the conditional percent (given cluster) are presented.

| Dataset | Variable | Traditional Cluster | Active Cluster | All Classes |
|---|---|---|---|---|
| Original | Campus | | | |
| | 1 | 4 (5%) | 11 (25%) | 15 (12%) |
| | 2 | 19 (23%) | 2 (5%) | 21 (17%) |
| | 3 | 58 (72%) | 31 (70%) | 89 (71%) |
| | | $n_0 = 81$ | $n_1 = 44$ | $n = 125$ |
| Analyzer | Campus | | | |
| | 1 | 15 (14%) | 0 (0%) | 15 (12%) |
| | 2 | 21 (19%) | 0 (0%) | 21 (17%) |
| | 3 | 58 (72%) | 31 (70%) | 89 (71%) |
| | | $n_0 = 108$ | $n_1 = 17$ | $n = 125$ |
| Collapsed | Campus | | | |
| | 1 | 11 (12%) | 4 (13%) | 15 (12%) |
| | 2 | 19 (23%) | 2 (5%) | 21 (17%) |
| | 3 | 64 (68%) | 25 (81%) | 89 (71%) |
| | | $n_0 = 94$ | $n_1 = 31$ | $n = 125$ |
| Novel | Campus | | | |
| | 1 | 4 (5%) | 11 (25%) | 15 (12%) |
| | 2 | 5 (9%) | 16 (24%) | 21 (17%) |
| | 3 | 48 (84%) | 41 (60%) | 89 (71%) |
| | | $n_0 = 57$ | $n_1 = 68$ | $n = 125$ |
| Unique | Campus | | | |
| | 1 | 4 (6%) | 11 (21%) | 15 (12%) |
| | 2 | 14 (19%) | 7 (13%) | 21 (17%) |
| | 3 | 54 (75%) | 35 (66%) | 89 (71%) |
| | | $n_0 = 72$ | $n_1 = 53$ | $n = 125$ |
| Final Clusters | Campus | | | |
| | 1 | 4 (5%) | 11 (23%) | 15 (12%) |
| | 2 | 18 (23%) | 3 (6%) | 21 (17%) |
| | 3 | 56 (72%) | 33 (70%) | 89 (71%) |
| | | $n_0 = 78$ | $n_1 = 47$ | $n = 125$ |

**Table S25** Summary Statistics for Discipline across the 5 Different Cluster Ensembles and the Final Clusters. Summary of discipline for the traditional-lecture and active-learning clusters. The number and the conditional percent (given cluster) are presented.

| Dataset | Variable | Traditional Cluster | Active Cluster | All Classes |
|---|---|---|---|---|
| Original | Discipline | | | |
| | Biological Sciences | 15 (18%) | 24 (54%) | 39 (31%) |
| | Physical Sciences | 32 (40%) | 4 (9%) | 36 (29%) |
| | I&C Sciences | 16 (20%) | 10 (23%) | 26 (21%) |
| | Engineering | 18 (22%) | 6 (14%) | 24 (19%) |
| | | $n_0 = 81$ | $n_1 = 44$ | $n = 125$ |
| Analyzer | Discipline | | | |
| | Biological Sciences | 33 (31%) | 6 (35%) | 39 (31%) |
| | Physical Sciences | 32 (30%) | 4 (24%) | 36 (29%) |
| | I&C Sciences | 21 (19%) | 5 (29%) | 26 (21%) |
| | Engineering | 22 (20%) | 2 (12%) | 24 (19%) |
| | | $n_0 = 108$ | $n_1 = 17$ | $n = 125$ |
| Collapsed | Discipline | | | |
| | Biological Sciences | 25 (27%) | 14 (45%) | 39 (31%) |
| | Physical Sciences | 33 (35%) | 3 (10%) | 36 (29%) |
| | I&C Sciences | 17 (18%) | 9 (29%) | 26 (21%) |
| | Engineering | 19 (20%) | 5 (16%) | 24 (19%) |
| | | $n_0 = 94$ | $n_1 = 31$ | $n = 125$ |
| Novel | Discipline | | | |
| | Biological Sciences | 11 (19%) | 28 (41%) | 39 (31%) |
| | Physical Sciences | 21 (37%) | 15 (22%) | 36 (29%) |
| | I&C Sciences | 11 (19%) | 15 (22%) | 26 (21%) |
| | Engineering | 14 (25%) | 10 (15%) | 24 (19%) |
| | | $n_0 = 57$ | $n_1 = 68$ | $n = 125$ |
| Unique | Discipline | | | |
| | Biological Sciences | 14 (19%) | 25 (47%) | 39 (31%) |
| | Physical Sciences | 25 (35%) | 11 (21%) | 36 (29%) |
| | I&C Sciences | 15 (21%) | 11 (21%) | 26 (21%) |
| | Engineering | 18 (25%) | 6 (11%) | 24 (19%) |
| | | $n_0 = 72$ | $n_1 = 53$ | $n = 125$ |
| Final Clusters | Discipline | | | |
| | Biological Sciences | 15 (19%) | 24 (51%) | 39 (31%) |
| | Physical Sciences | 30 (38%) | 6 (13%) | 36 (29%) |
| | I&C Sciences | 15 (19%) | 11 (23%) | 26 (21%) |
| | Engineering | 18 (23%) | 6 (13%) | 24 (19%) |
| | | $n_0 = 78$ | $n_1 = 47$ | $n = 125$ |

**Table S26** Summary Statistics for Class Size across the 5 Different Cluster Ensembles and the Final Clusters. Summary of class size for the traditional-lecture and active-learning clusters. The number and the conditional percent (given cluster) are presented.

| Dataset | Variable | Traditional Cluster | Active Cluster | All Classes |
|---|---|---|---|---|
| Original | Class Size | | | |
| | Small (0-99) | 17 (21%) | 16 (36%) | 33 (26%) |
| | Medium (100-199) | 35 (43%) | 8 (18%) | 43 (34%) |
| | Large (200+) | 29 (36%) | 20 (45%) | 49 (39%) |
| | | $n_0 = 81$ | $n_1 = 44$ | $n = 125$ |
| Analyzer | Class Size | | | |
| | Small (0-99) | 21 (19%) | 12 (71%) | 33 (26%) |
| | Medium (100-199) | 41 (38%) | 2 (12%) | 43 (34%) |
| | Large (200+) | 46 (43%) | 3 (18%) | 49 (39%) |
| | | $n_0 = 108$ | $n_1 = 17$ | $n = 125$ |
| Collapsed | Class Size | | | |
| | Small (0-99) | 18 (19%) | 15 (48%) | 33 (26%) |
| | Medium (100-199) | 38 (40%) | 5 (16%) | 43 (34%) |
| | Large (200+) | 38 (40%) | 11 (35%) | 49 (39%) |
| | | $n_0 = 94$ | $n_1 = 31$ | $n = 125$ |
| Novel | Class Size | | | |
| | Small (0-99) | 11 (19%) | 22 (32%) | 33 (26%) |
| | Medium (100-199) | 24 (42%) | 19 (28%) | 43 (34%) |
| | Large (200+) | 22 (39%) | 27 (40%) | 49 (39%) |
| | | $n_0 = 57$ | $n_1 = 68$ | $n = 125$ |
| Unique | Class Size | | | |
| | Small (0-99) | 13 (18%) | 20 (38%) | 33 (26%) |
| | Medium (100-199) | 32 (44%) | 11 (21%) | 43 (34%) |
| | Large (200+) | 27 (38%) | 22 (42%) | 49 (39%) |
| | | $n_0 = 72$ | $n_1 = 53$ | $n = 125$ |
| Final Clusters | Class Size | | | |
| | Small (0-99) | 16 (21%) | 17 (36%) | 33 (26%) |
| | Medium (100-199) | 34 (44%) | 9 (19%) | 43 (34%) |
| | Large (200+) | 28 (36%) | 21 (45%) | 49 (39%) |
| | | $n_0 = 78$ | $n_1 = 47$ | $n = 125$ |

**Table S27** Original COPUS Codes Logistic Regression Model for Active-Learning Cluster. The coefficients represent the increase/decrease in the odds of being in the active-learning cluster (based on the original codes) for each of the variables of interest (while holding the other variables in the model constant). The reference group (RG) are labeled for each of the categorical variables.

| | Estimated Odds | 95% Confidence Interval | Test Statistic | p-value | |
|---|---|---|---|---|---|
| Intercept | 10.29 | (1.46, 72.55) | 2.34 | 0.02 | * |
| Faculty Type | | | | | |
| RG: Teaching Faculty | | | | | |
| Research Faculty | 0.29 | (0.08, 1.03) | -1.92 | 0.06 | |
| Lecturers | 0.32 | (0.08, 1.22) | -1.67 | 0.10 | |
| Faculty Rank | | | | | |
| RG: Assistant | | | | | |
| Associate | 0.47 | (0.11, 2.12) | -0.98 | 0.33 | |
| Full | 0.61 | (0.11, 3.56) | -0.54 | 0.59 | |
| Years of teaching | 1.05 | (0.96, 1.16) | 1.14 | 0.25 | |
| Gender | | | | | |
| RG: non-female | | | | | |
| Female | 1.17 | (0.40, 3.42) | 0.29 | 0.77 | |
| Campus | | | | | |
| RG: Campus 3 | | | | | |
| Campus 2 | 0.13 | (0.02, 0.86) | -2.12 | 0.03 | * |
| Campus 1 | 2.25 | (0.37, 13.80) | 0.88 | 0.38 | |
| Discipline | | | | | |
| RG: Biological Sciences | | | | | |
| Engineering | 0.28 | (0.06, 1.35) | -1.59 | 0.11 | |
| I&C Sciences | 0.47 | (0.10, 2.18) | -0.96 | 0.34 | |
| Physical Sciences | 0.06 | (0.01, 0.31) | -3.35 | $< 0.001$ | * |
| Class Size | | | | | |
| RG: Small (0-99) | | | | | |
| Medium (100-199) | 0.12 | (0.03, 0.50) | -2.91 | $< 0.001$ | * |
| Large (200+) | 0.22 | (0.06, 0.76) | -2.40 | 0.02 | * |
| AIC = 139.20 | | | | | |

**Table S28** Analyzer COPUS Codes Logistic Regression Model for Active-Learning Cluster. The coefficients represent the increase/decrease in the odds of being in the active-learning cluster (based on the analyzer codes) for each of the variables of interest (while holding the other variables in the model constant). The reference group (RG) are labeled for each of the categorical variables.

| | Estimated Odds | 95% Confidence Interval | Test Statistic | p-value | |
|---|---|---|---|---|---|
| Intercept | 5.73 | (0.48, 68.63) | 1.38 | 0.17 | |
| Faculty Type | | | | | |
| RG: Teaching Faculty | | | | | |
| Research Faculty | 0.49 | (0.06, 3.89) | -0.67 | 0.50 | |
| Lecturers | 0.19 | (0.02, 1.52) | -1.57 | 0.12 | |
| Faculty Rank | | | | | |
| RG: Assistant | | | | | |
| Associate | 0.17 | (0.02, 1.40) | -1.65 | 0.10 | |
| Full | 0.02 | (0.00, 0.35) | -2.61 | 0.01 | * |
| Years of teaching | 1.16 | (0.99, 1.36) | 1.81 | 0.07 | |
| Gender | | | | | |
| RG: non-female | | | | | |
| Female | 0.76 | (0.14, 4.25) | -0.31 | 0.76 | |
| Campus | | | | | |
| RG: Campus 3 | | | | | |
| Campus 2 | 0.00 | $(0.00, \infty)$ | – | – | |
| Campus 1 | 0.00 | $(0.00, \infty)$ | – | – | |
| Discipline | | | | | |
| RG: Biological Sciences | | | | | |
| Engineering | 0.14 | (0.01, 2.07) | -1.43 | 0.15 | |
| I&C Sciences | 0.48 | (0.04, 5.09) | -0.61 | 0.54 | |
| Physical Sciences | 0.51 | (0.07, 3.78) | -0.66 | 0.51 | |
| Class Size | | | | | |
| RG: Small (0-99) | | | | | |
| Medium (100-199) | 0.05 | (0.01, 0.47) | -2.66 | 0.01 | * |
| Large (200+) | 0.03 | (0.00, 0.25) | -3.26 | $< 0.001$ | * |
| AIC = 77.85 | | | | | |

**Table S29** Collapsed COPUS Codes Logistic Regression Model for Active-Learning Cluster. The coefficients represent the increase/decrease in the odds of being in the active-learning cluster (based on the collapsed codes) for each of the variables of interest (while holding the other variables in the model constant). The reference group (RG) are labeled for each of the categorical variables.

| | Estimated Odds | 95% Confidence Interval | Test Statistic | p-value | |
|---|---|---|---|---|---|
| Intercept | 44.54 | (3.95, 502.40) | 3.07 | < 0.001 | * |
| Faculty Type | | | | | |
| RG: Teaching Faculty | | | | | |
| Research Faculty | 0.12 | (0.03, 0.54) | -2.78 | 0.01 | * |
| Lecturers | 0.09 | (0.02, 0.43) | -3.00 | < 0.001 | * |
| Faculty Rank | | | | | |
| RG: Assistant | | | | | |
| Associate | 0.18 | (0.03, 0.95) | -2.03 | 0.04 | * |
| Full | 0.13 | (0.02, 0.98) | -1.98 | 0.05 | * |
| Years of teaching | 0.97 | (0.88, 1.08) | -0.50 | 0.62 | |
| Gender | | | | | |
| RG: non-female | | | | | |
| Female | 2.29 | (0.62, 8.46) | 1.24 | 0.22 | |
| Campus | | | | | |
| RG: Campus 3 | | | | | |
| Campus 2 | 0.10 | (0.01, 0.80) | -2.17 | 0.03 | * |
| Campus 1 | 0.24 | (0.03, 1.90) | -1.35 | 0.18 | |
| Discipline | | | | | |
| RG: Biological Sciences | | | | | |
| Engineering | 0.39 | (0.05, 3.01) | -0.90 | 0.37 | |
| I&C Sciences | 0.65 | (0.10, 4.07) | -0.46 | 0.64 | |
| Physical Sciences | 0.08 | (0.01, 0.55) | -2.56 | 0.01 | * |
| Class Size | | | | | |
| RG: Small (0-99) | | | | | |
| Medium (100-199) | 0.08 | (0.02, 0.43) | -2.96 | < 0.001 | * |
| Large (200+) | 0.09 | (0.02, 0.41) | -3.10 | < 0.001 | * |
| AIC = 111.33 | | | | | |

**Table S30** Novel COPUS Codes Logistic Regression Model for Active-Learning Cluster. The coefficients represent the increase/decrease in the odds of being in the active-learning cluster (based on the novel codes) for each of the variables of interest (while holding the other variables in the model constant). The reference group (RG) are labeled for each of the categorical variables.

| | Estimated Odds | 95% Confidence Interval | Test Statistic | p-value | |
|---|---|---|---|---|---|
| Intercept | 11.06 | (1.64, 74.54) | 2.47 | 0.01 | * |
| Faculty Type | | | | | |
| RG: Teaching Faculty | | | | | |
| Research Faculty | 0.13 | (0.04, 0.44) | -3.30 | $< 0.001$ | * |
| Lecturers | 0.19 | (0.05, 0.70) | -2.50 | 0.01 | * |
| Faculty Rank | | | | | |
| RG: Assistant | | | | | |
| Associate | 0.80 | (0.19, 3.26) | -0.32 | 0.75 | |
| Full | 0.62 | (0.12, 3.15) | -0.58 | 0.57 | |
| Years of teaching | 1.00 | (0.91, 1.09) | -0.01 | 0.99 | |
| Gender | | | | | |
| RG: non-female | | | | | |
| Female | 1.29 | (0.50, 3.34) | 0.52 | 0.60 | |
| Campus | | | | | |
| RG: Campus 3 | | | | | |
| Campus 2 | 7.91 | (1.82, 34.36) | 2.76 | 0.01 | * |
| Campus 1 | 1.66 | (0.27, 10.35) | 0.54 | 0.59 | |
| Discipline | | | | | |
| RG: Biological Sciences | | | | | |
| Engineering | 0.50 | (0.10, 2.37) | -0.88 | 0.38 | |
| I&C Sciences | 0.94 | (0.21, 4.23) | -0.08 | 0.93 | |
| Physical Sciences | 0.20 | (0.05, 0.82) | -2.23 | 0.03 | * |
| Class Size | | | | | |
| RG: Small (0-99) | | | | | |
| Medium (100-199) | 0.46 | (0.14, 1.50) | -1.29 | 0.20 | |
| Large (200+) | 0.50 | (0.16, 1.63) | -1.15 | 0.25 | |
| AIC = 158.99 | | | | | |

**Table S31** Unique COPUS Codes Logistic Regression Model for Active-Learning Cluster. The coefficients represent the increase/decrease in the odds of being in the active-learning cluster (based on the unique codes) for each of the variables of interest (while holding the other variables in the model constant). The reference group (RG) are labeled for each of the categorical variables.

| | Estimated Odds | 95% Confidence Interval | Test Statistic | p-value | |
|---|---|---|---|---|---|
| Intercept | 7.37 | (1.26, 43.12) | 2.22 | 0.03 | * |
| Faculty Type | | | | | |
| RG: Teaching Faculty | | | | | |
| Research Faculty | 0.28 | (0.09, 0.90) | -2.14 | 0.03 | * |
| Lecturers | 0.34 | (0.10, 1.11) | -1.78 | 0.07 | |
| Faculty Rank | | | | | |
| RG: Assistant | | | | | |
| Associate | 0.53 | (0.14, 1.96) | -0.95 | 0.34 | |
| Full | 0.63 | (0.13, 3.10) | -0.57 | 0.57 | |
| Years of teaching | 1.02 | (0.93, 1.11) | 0.37 | 0.71 | |
| Gender | | | | | |
| RG: non-female | | | | | |
| Female | 1.90 | (0.73, 4.96) | 1.31 | 0.19 | |
| Campus | | | | | |
| RG: Campus 3 | | | | | |
| Campus 2 | 0.63 | (0.17, 2.30) | -0.71 | 0.48 | |
| Campus 1 | 2.28 | (0.42, 12.52) | 0.95 | 0.34 | |
| Discipline | | | | | |
| RG: Biological Sciences | | | | | |
| Engineering | 0.32 | (0.07, 1.41) | -1.51 | 0.13 | |
| I&C Sciences | 0.66 | (0.16, 2.65) | -0.59 | 0.55 | |
| Physical Sciences | 0.30 | (0.08, 1.06) | -1.86 | 0.06 | |
| Class Size | | | | | |
| RG: Small (0-99) | | | | | |
| Medium (100-199) | 0.19 | (0.06, 0.61) | -2.79 | 0.01 | * |
| Large (200+) | 0.25 | (0.08, 0.75) | -2.46 | 0.01 | * |
| AIC = 161.96 | | | | | |

# References

Baker, F.B., & Hubert, L.J. (1975). Measuring the power of hierarchical cluster analysis. *Journal of the American Statistical Association*, *70*(349), 31-38. Retrieved from https://www.tandfonline.com/doi/abs/10.1080/01621459.1975.10480256 https://arxiv.org/abs/https://www.tandfonline.com/doi/pdf/10.1080/01621459.1975.10480256 10.1080/01621459.1975.10480256

Caliński, T., & Harabasz, J. (1974). A dendrite method for cluster analysis. *Communications in Statistics Simulation and Computation*, *3*(1), 1–27.

Davies, D.L., & Bouldin, D.W. (1979). A cluster separation measure. *IEEE Trans. Pattern Anal. Mach. Intell.*, *1*(2), 224-227. Retrieved from $http://dblp.uni-trier.de/db/journals/pami/pami1.html\#DaviesB79$

Dunn, J.C. (1974). Well-separated clusters and optimal fuzzy partitions. *Journal of Cybernetics*, *4*(1), 95-104. Retrieved from https://doi.org/10.1080/01969727408546059 https://arxiv.org/abs/https://doi.org/10.1080/01969727408546059 10.1080/01969727408546059

Halkidi, M., Batistakis, Y., Vazirgiannis, M. (2001). On clustering validation techniques. *Journal of Intelligent Information Systems*, *17*, 107–145.

Halkidi, M., & Vazirgiannis, M. (2001). Clustering validity assessment: Finding the optimal partitioning of a data set. N. Cercone, T.Y. Lin, & X. Wu (Eds.), *Icdm* (p. 187-194). IEEE Computer Society.

Handl, J., Knowles, J.D., Kell, D.B. (2005). Computational cluster validation in post-genomic data analysis. *Bioinform.*, *21*(15), 3201-3212. Retrieved from $http://dblp.uni-trier.de/db/journals/bioinformatics/bioinformatics21.html\#HandlKK05$

Hubert, L., & Schultz, J. (1976). Quadratic assignment as a general data analysis strategy. *British Journal of Mathematical and Statistical Psychology*, *29*(2), 190–241. Retrieved from $http://scholar.google.de/scholar.bib?q=info:eTTf7MNZSOsJ:scholar.google.com/\&output=citation\&hl=de\&as_sdt=0,5\&ct=citation\&cd=0$

Hubert, L.J., & Levin, J.R. (1976, 10). A general statistical framework for assessing categorical clustering in free recall. *Psychological Bulletin*, *83*(6), 1072–1080.

10.1037/0033-2909.83.6.1072

Iam-on, N., & Garrett, S. (2010). LinkCluE: A MATLAB Package for Link-Based Cluster Ensembles. *Journal of Statistical Software*, *36*(i09). Retrieved from https://ideas.repec.org/a/jss/jstsof/v036i09.html

http://hdl.handle.net/10.

McClain, J.O., & Rao, V.R. (1975). CLUSTISZ: A program to test for the quality of clustering of a set of objects. *Journal of Marketing Research*, *12*(4), 456–460. Retrieved from http://www.jstor.org/stable/3151097

Milligan, G.W. (1981, 6). A monte carlo study of thirty internal criterion measures for cluster analysis. *Psychometrika*, *46*(2), 187-199.

10.1007/BF02293899

Pakhira, M.K., Bandyopadhyay, S., Maulik, U. (2004). Validity index for crisp and fuzzy clusters. *Pattern Recognit.*, *37*(3), 487-501. Retrieved from $http : //dblp.uni - trier.de/db/journals/pr/pr37.html\#PakhiraBM04$

Ray, S., & Turi, R.H. (1999). Determination of number of clusters in k-means clustering and application in colour segmentation. *The 4th international conference on advances in pattern recognition and digital techniques* (pp. 137–143).

Rohlf, F.J. (1974). Methods of comparing classifications. *Annual Review of Ecology and Systematics*, *5*, 101-113.

Rousseeuw, P. (1987). Silhouettes: a graphical aid to the interpretation and validation of cluster analysis. *J. Comput. Appl. Math.*, *20*(1), 53–65. Retrieved from http://portal.acm.org/citation.cfm?id=38772

http://dx.doi.org/10.1016/0377-0427(87)90125-7
